# Epromoters bind key stress-related transcription factors to regulate clusters of stress response genes

**DOI:** 10.1038/s44318-025-00670-3

**Published:** 2026-01-03

**Authors:** Juliette Malfait, Jing Wan, Himanshu Narayan Singh, Charbel Souaid, Gaëlle Farah, Junhua Su, Magali Torres, Iris Manosalva, Nathalie Sakakini, Cyril Esnault, Sandrine Sarrazin, Michael Sieweke, Salvatore Spicuglia

**Affiliations:** 1 Aix-Marseille University, INSERM, TAGC, UMR1090, Marseille, France; 2 Equipe Labellisée Ligue Contre le Cancer, Marseille, France; 3 University of Montpellier, CNRS, IGMM, UMR5535, Montpellier, France; 4 Aix Marseille University, CNRS, INSERM, CIML, Marseille, France; 5 Center for Regenerative Therapies Dresden (CRTD), Technical University Dresden, Dresden, Germany

**Keywords:** Epromoter, Stress Response, Gene Cluster, Gene Regulation, Chromatin, Transcription & Genomics, Computational Biology

## Abstract

Cellular and environmental stress triggers the rapid and global reprogramming of gene transcription by coordinated recruitment of a limited number of key inducible transcription factors to *cis*-regulatory elements. Here, we performed a comprehensive analysis of different stress models and observed that co-induced genes are generally located in close genomic proximity. By integrating gene expression and transcription factor binding resources across different stress models, we identify an enrichment for clusters in which only one of the clusters’ promoters recruits the key transcription factors, reminiscent of Epromoters—a type of *cis*-regulatory element that displays both promoter and enhancer function. Epromoter-regulated clusters were frequently found regardless of the stress or inflammatory response. Predicted Epromoters displayed enhancer activity and regulated clusters of stress-response genes independently of their genomic location. These findings imply that Epromoters are central regulatory elements that control gene clusters in response to acute perturbations.

## Synopsis

Stress responses rewire transcription through specialized regulatory elements. This study reveals that Epromoters—dual elements with promoter-enhancer functions—orchestrate coordinated transcriptional responses of clustered genes to diverse stress signals.

- Co-induced stress-response genes are often located in close genomic proximity.
- Only one promoter per cluster typically recruits key stress-activated transcription factors.
- These “hub” promoters show enhancer-like activity, consistent with Epromoter function.
- Epromoter-regulated clusters occur across different stress and inflammatory models.
- Epromoters enable coordinated regulation of gene clusters independently of genomic context.

## Abstract (web summary)

Epromoters bind key stress-related transcription factors to regulate clusters of stress response genes.

## Graphical abstract

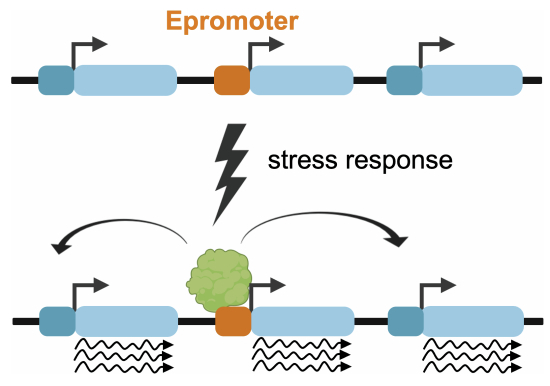

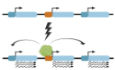

## Introduction

Cells and organisms are constantly exposed to extrinsic and intrinsic stressors that endanger homeostasis and fitness (Plosky, 2024). Such stresses can be induced by hypoxia, toxins, mechanical stimuli, elevated temperature or pathogen infections, which all compromise cell structure and function by causing macromolecular damage. To survive such stress insults, cells launch survival programs that are characterized by the activation of rapid and transient transcriptional reprogramming (Pessa et al, 2024). In contrast to cell differentiation programs, stress programs are mediated by a limited number of inducible transcription factors (Galluzzi et al, 2018; Vihervaara et al, 2018). For instance, heat-shock (HS) response is mediated by the HSF1 and HSF2 TFs, Hypoxia is regulated by the HIF1 complex and TNFα response by the NF-κB complex. Moreover, previous observations have suggested that co-induced stress-response genes are frequently organized in clusters, suggesting the sharing of *cis*-regulatory elements (Ebisuya et al, 2008; Feuerborn and Cook, 2015; Ghanbarian and Hurst, 2015; M Ribeiro et al, 2022; Seufert et al, 2024; Siwek et al, 2020).

Gene transcription in higher eukaryotes relies on a diverse network of regulatory elements, including promoters and enhancers, which traditionally have been seen as distinct types of regulatory elements. Promoters, located near the transcription start site (TSS), initiate local gene transcription, whereas enhancers, positioned farther from the TSS, activate gene expression over larger distances. However, their distinction has become more blurred in recent years notably by the discovery of Epromoters, *cis*-regulatory sequences that combine architectural and functional characteristics of both promoters and enhancers (Arnold et al, 2013; Corrales et al, 2017; Dao et al, 2017; Diao et al, 2017; Engreitz et al, 2016; Malfait et al, 2023; Nguyen et al, 2016; Rajagopal et al, 2016; Santiago-Algarra et al, 2021; Zabidi et al, 2015). Epromoters can regulate distal promoters when assessed in episomal reporter systems or high-throughput reporter assays in different cellular contexts from drosophila to humans (Arnold et al, 2013; Dao et al, 2017; Muerdter et al, 2018; Nguyen et al, 2016; Santiago-Algarra et al, 2021; Zabidi et al, 2015), while their deletion or epigenetic silencing in their natural context results in the loss of expression of distal genes (Dao et al, 2017; Diao et al, 2017; Engreitz et al, 2016; Ng et al, 2024; Rajagopal et al, 2016; Santiago-Algarra et al, 2021). Moreover, genetic variation within Epromoters can potentially influence the expression of distal genes (Dao et al, 2017; Jung et al, 2019; Mitchelmore et al, 2020; Saint Just Ribeiro et al, 2022; Wang et al, 2018).

Previous studies have suggested a link between the Epromoter function and stress responses, particularly the regulation of interferon-response genes (Dao et al, 2017; Dao and Spicuglia, 2018; Muerdter et al, 2018; Santiago-Algarra et al, 2021). Using high-throughput reporter assays, we previously showed that interferon alpha (IFNα)-induced genes are frequently organized in clusters regulated by a single Epromoter (Santiago-Algarra et al, 2021). Remarkably, within these clusters, Epromoters function as regulatory hubs that selectively recruit transcription factors (TFs) essential for IFNα-driven responses, coordinating gene expression across the cluster. It is likely that this type of regulatory element could be more generally involved in the rapid response of genes to cellular stress. This idea fits well with the notion that co-regulated genes are often located in close genomic proximity of defining inducible transcription factories containing a high concentration of RNA polymerase and key TFs, and where efficient transcription can be triggered (Cook and Marenduzzo, 2018; Corrales et al, 2017; Dejosez et al, 2023; Feuerborn and Cook, 2015; Rippe and Papantonis, 2025; Santiago-Algarra et al, 2021). Noteworthy, many of the regulatory elements of inducible genes, such as metallothioneins, histones of early cleavage stages, viral immediate-early genes (from some papovaviruses, cytomegaloviruses, and retroviruses), heat-shock genes and the antiviral interferon genes are located close to, or overlapping with, the promoter region of these genes (Medina-Rivera et al, 2018; Schaffner, 2015). A common characteristic of most of the aforementioned promoters is that they are associated with inducible genes that have to quickly respond to environmental stress.

By analyzing a comprehensive dataset of stress and inflammatory responses from different cellular and environmental cues in mice and humans, we found that induced genes are preferentially found in close genomic proximity. We hypothesized that stress-induced genes are globally organized into clusters, with Epromoters regulating the clustered genes by recruiting key inducible transcription factors to obtain a fast and coordinated response to stress insults. To explore Epromoter function under different stress conditions, we developed a pipeline to identify Epromoters and their potential target genes across various stress conditions, bypassing the need for high-throughput enhancer assays. By retrieving transcriptomic and ChIP-seq data from 56 studies, we identified Epromoter-regulated clusters across 20 distinct stress conditions. Reporter assays and CRISPR-Cas9 genomic deletions confirmed Epromoter activity and demonstrated their regulatory influence on neighboring genes within these clusters at their endogenous loci. Additionally, repositioning an Epromoter within its locus demonstrated its intrinsic enhancer and promoter functions, underscoring the dual role of Epromoters in gene regulation. Thus, Epromoters have a broad role in the induction of co-regulated genes during the mammalian stress response.

## Results

### Stress-response genes are preferentially found in genome proximity

To better understand the regulation of stress-response genes, we selected a series of published stress-response datasets from human and mouse, including gene expression data (RNA-seq, Pro-seq or microarrays) and ChIP-seq for key transcription factors involved in the same stress responses. We compiled data from 32 studies, comprising 36 stress or stimulation conditions for a total of 56 datasets (Biddie et al, 2011; Brown et al, 2014; Camps et al, 2014; Cardamone et al, 2018; Ebisuya et al, 2008; Esnault et al, 2014; Ferrari et al, 2020; Franco et al, 2015; Gualdrini et al, 2016; Hancock et al, 2019; Hogan et al, 2017; Jin et al, 2013; Jubb et al, 2016; Jurida et al, 2015; Kusnadi et al, 2019; Langlais et al, 2016; Lo et al, 2013; Lyu et al, 2018; Mahat et al, 2016; Mancino et al, 2015; Park et al, 2017; Phanstiel et al, 2017; Piccolo et al, 2017; Porter et al, 2017; Purbey et al, 2024; Ramos Pittol et al, 2018; Santiago-Algarra et al, 2021; Schmidt et al, 2015; Vierbuchen et al, 2017; Vihervaara et al, 2017) (Table EV1A). The datasets cover 20 different types of stress or stimulation conditions, such as heat shock, serum starvation/response, hypoxia, TNFα, IFNα, IFNγ, and LPS. For example, the “Vihervaara” dataset (Vihervaara et al, 2017) consisted of Pro-seq data and ChIP-seq of transcription factors HSF1 and HSF2 before and after K562 cells were exposed to HS at 42 °C for 30 min.

**Figure 1:**
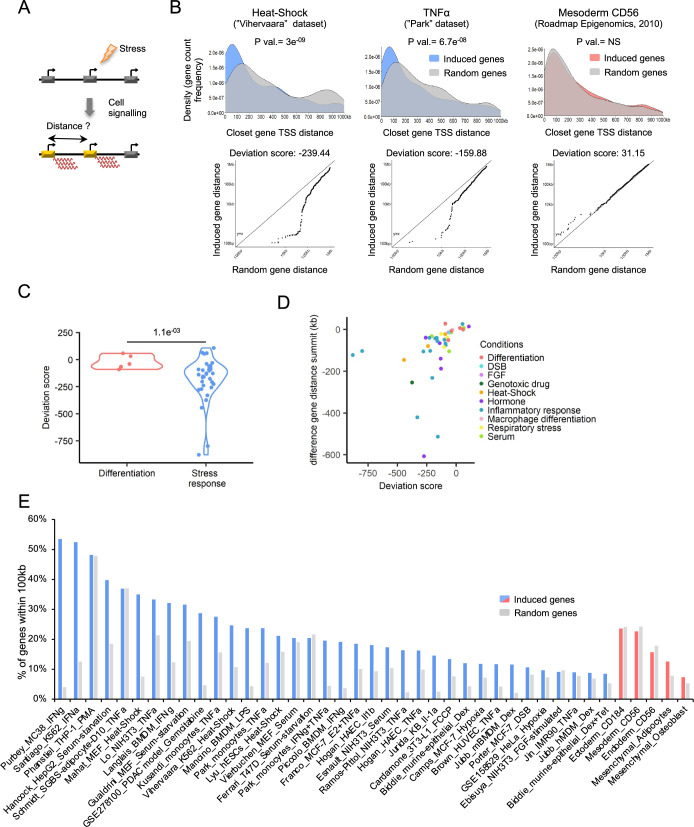
Genomic distance between stress responses induced genes. (**A**) Approach to assess the distance between two induced genes upon stress. (**B**) (top panel) Distance distribution of the induced genes (colors) compared to the same numbers of random genes (gray) by dataset (blue: heat shock, and TNFα, salmon: Mesoderm). *P* values were calculated by Kolmogorov–Smirnov (KS) test. (bottom panel) Deviation scores were calculated between the distance distribution of induced and random genes. (**C**) Violin plot of the deviation score between datasets from embryonic stem (ES) and mesenchymal differentiation (pink) or stress responses datasets (blue). Each point represents a dataset (5 for the differentiation and 36 for stress responses). *P* value was calculated by a two-sided Student’s t-test. (**D**) Distribution of each dataset deviation score (x-axis) compared to their difference between the induced and random gene distance summit (y-axis). The dataset is classified into 10 conditions highlighted by different colors (salmon: ES and Mesenchymal differentiation, light blue: double-strand breaks (DSB), light purple: fibroblast growth factors (FGF), dark green: genotoxic drug, orange: heat shock, purple: hormone, blue: inflammatory response, pink: macrophage differentiation, yellow: respiratory stress, light green: serum. (**E**) Barplot of the percentage of genes within 100 kb between the induced (blue: stress responses, salmon: ES and Mesenchymal differentiation) and the random (gray) genes for each dataset. Source data are available online for this figure.

**Figure EV1:**
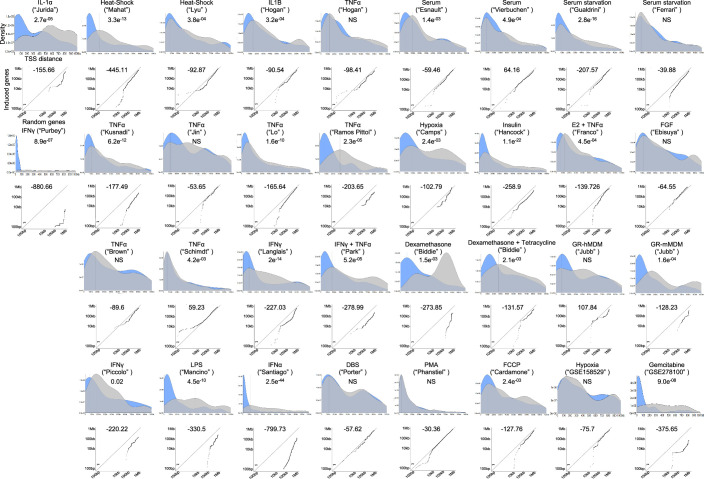
Genomic distance between stress responses induced genes for all stress-related datasets, related to Fig. 1. The top panels display the distance distribution of the induced genes (blue) compared to the same numbers of random genes (gray) by dataset. The bottom panels display the deviation score calculated between induced gene distance and random gene distance for each dataset. The *P* values for the distance distribution were calculated by Kolmogorov–Smirnov (KS) test.

**Figure EV2:**
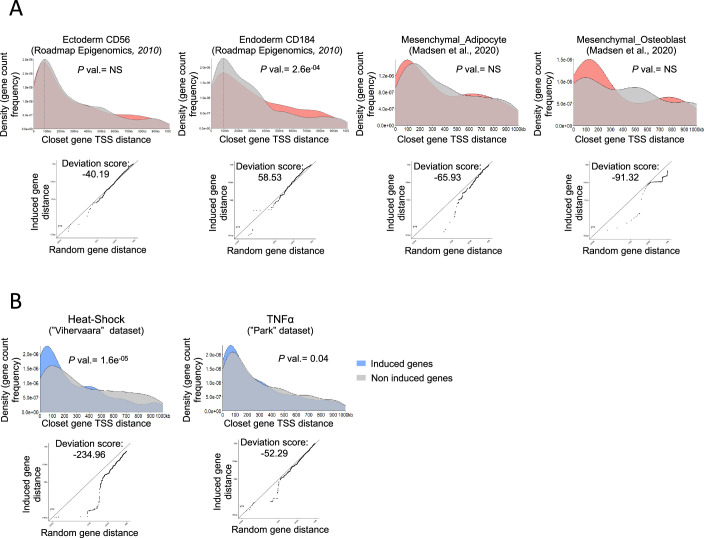
Genomic distance between induced genes for control datasets, related to Fig. 1. (**A**) The top panels display the distance distribution of the induced genes (salmon) compared to the same numbers of random genes (gray) for each dataset of differentiation. The bottom panels display the deviation score calculated between the induced and random gene distances for each dataset. *P* values were calculated by Kolmogorov–Smirnov (KS) test. (**B**) The top panels display the distance distribution of the induced genes (blue) compared to the same numbers of non-induced genes for the same dataset (gray). The bottom panels display the deviation score calculated between induced gene distance and non-induced gene distance for each dataset. *P* values were calculated by Kolmogorov–Smirnov (KS) test.

Previous studies have suggested that Epromoters might regulate co-induced genes located in genome proximity (Santiago-Algarra et al, 2021). Therefore, we first aimed to determine whether there was a bias in the genomic distribution of stress-induced genes. We calculated the distance between the transcription start sites (TSS) of the closest gene pairs for each set of stress-induced genes (Fig. 1A) and compared their distance distribution to the same number of randomly selected genes (Fig. 1B upper panels; Fig. EV1). As a control, we analyzed data from human embryonic stem cell (ESC) differentiation into three germ layers (mesoderm, ectoderm, and endoderm) (Bernstein et al, 2010), as well as in vitro differentiation of human mesenchymal stromal cells into either adipocytes or osteoblasts (Madsen et al, 2020). We observed that the majority of stress-induced genes were located closer to each other compared to a random distance distribution. For example, HS (“Vihervaara” dataset) and TNFα (“Park” dataset) induced genes have a distribution summit of 57.6 kb and 63.8 kb as compared with 136 kb and 119 kb for random genes, respectively (*P* value: 3e^−09^ and 6.7e^−08^; respectively, K-S test). However, no significant differences were observed for the distance distribution of induced genes after ESC or mesenchymal cells differentiation (Fig. 1B upper panels; Fig. EV2A).

To assess the distribution biases more precisely, we also calculated a deviation score based on the difference in TSS distances between the random and induced genes (Fig. 1B, bottom panels; Figs. EV1–EV2A). The deviation score could be positive or negative depending on whether induced genes are more distant or closer than random genes, respectively. For instance, the HS- and TNFα-induced genes have a negative deviation score of −239.44 and −159.88, respectively, while the mesoderm-induced genes have a positive deviation score of 31.15. Globally, stress response genes have a significantly lower deviation score than genes induced after ESC or mesenchymal differentiation processes, indicating a closer distance between stress-induced genes (Fig. 1C,D). For the majority of the stress models, we also found more genes located within 100 kb from each other than for random selected genes, but this was not the case for ESC or mesenchymal differentiation (Fig. 1E). We noted, however, that 9 out of 36 stress conditions displayed higher or similar distant distribution than differentiation-induced genes, highlighting variability in gene organization depending on the type of stress (Figs. 1C–E and EV1–EV2A). Finally, the HS- and TNFα-induced genes displayed a closer distance distribution than a set of genes with similar expression, but not induced by the stress treatment (Fig. EV2B). Taken together, these results suggest that genes induced by the stress response tend to be located in proximity, suggesting they might be organized into stress-response clusters of co-regulated genes.

### Identification of HS clusters regulated by Epromoters

**Figure 2:**
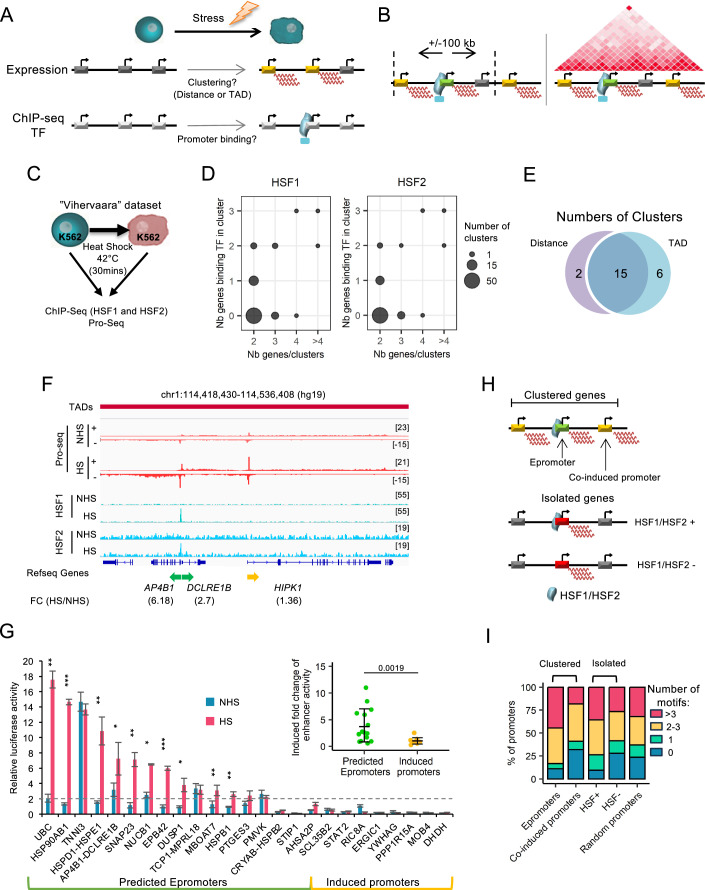
Identification and validation of Epromoter-regulated clusters in the HS stress response. (**A**) Schematic representation of the pipeline steps to identify Epromoter-regulated gene clusters. (**B**) Schematic overview of the two clustering methods used in the pipeline. (Left) Clustering of stress-induced genes based on the proximity of their TSS within a 100 kb distance. (Right) Clustering of stress-induced genes based on their localization within the same TAD. (**C**) Schematic representation of the “Vihervaara” dataset to induce the HS response. (**D**) (left) Bubble plot showing the number of all the clusters identified by their number of genes (x-axis) compared to their number of genes recruiting HSF1 (y-axis) determined by the pipeline. (right) Bubble plot showing the number of all the clusters identified by their number of genes (x-axis) compared to their number of genes recruiting HSF2 (y-axis) determined by the pipeline. (**E**) Venn diagram displaying the overlap of Epromoter-regulated clusters identified after clustering either by the distance between the TSS or their localization within the same TAD in the HS response. (**F**) Example of the *AP4B1-DCLRE1B* Epromoter-regulated cluster identified by the pipeline with the two clustering methods in the HS stress response. The genomic tracks show the PRO-seq signal (red) and HSF1 and HSF2 ChIP-seq signal (blue) before or after HS from the “Vihervaara” dataset. The topological associating domains (TAD) from K562 is displayed on the top. The fold-change of induction is indicated below the name and orientation of the genes (Epromoter: green, co-induced genes: yellow). (**G**) Luciferase assays to quantify the enhancer activity of predicted Epromoter (on the left) and induced promoters clustered with Epromoters (on the right) before (blue) and after (red) HS in the K562 cells. The results were normalized on the pGL4-SV40 promoter plasmid. All data represent mean values ± SD of three biological replicates. *P* values were calculated by a paired one-sided Student’s t-test, ***P < 0.001, **P < 0.01, *P < 0.1. Significant *P* values are as follows: *UBC* (0.0018), *HSP90AB1* (5.41e^−05^), *HSPD1-HSPE1* (0.0054), *AP4B1-DCLRE1B* (0.016), *SNAP23* (0.0022), *NUCB1* (0.028), *EPB42* (0.00072), *DUSP1* (0.013), *MBOAT7* (0.0091), *HSPB1* (0.0035). (insert) Luciferase assay-induced enhancer activity is represented by the fold change before/after HS for the predicted Epromoter and the induced promoters. *P* values were calculated by a two-sided Wilcoxon’s test. (**H**,** I**) Representation of the classification of the HS-induced genes in 4 categories (**H**). Percent stacked barplot of the numbers of motifs (HSE) per promoter in each category (**I**). The number of motifs is divided into 0 motif (blue), 1 motif (cyan), 2 to 3 motifs (yellow), and more than 3 motifs (red). Source data are available online for this figure.

We previously observed that within IFNα-induced clusters, Epromoters, functionally identified by STARR-seq, preferentially recruited the key TFs STAT1/2 and IRF1/9 (Santiago-Algarra et al, 2021). We wondered whether we could take advantage of this observation to predict Epromoters in different stress or stimulatory conditions. We reasoned that within a cluster of induced genes in response to extracellular or intracellular signaling, the promoter that preferentially recruits the key TFs can be predicted to function as an Epromoter. We developed a pipeline to predict promoters with Epromoter activity upon a stress response (Fig. 2A). First, we identified clusters of induced genes in two ways: either the TSS of induced genes were located less than 100 kb from each other, or they were within the same topologically associating domain (TAD) (Rao et al, 2014) (Fig. 2A,B). The threshold of 100 kb was defined based on the distance distribution summit between induced genes (range between 50 kb and 90 kb; Figs. 1 and EV1) as well as previous findings about *cis*-regulatory networks (Esnault et al, 2014; Gasperini et al, 2019; Ribeiro et al, 2021; Santiago-Algarra et al, 2021). Secondly, we assessed whether the key stress TF binding occurred within a [−1; +1] kb window centered on the TSS. We then counted how many promoters within each cluster bound the TF, considering a cluster potentially regulated by an Epromoter if the number of induced genes was higher than the number of genes displaying TF binding at their promoters (Fig. 2A; see Methods section for details). We used this prediction to explore the function of Epromoters in the different stress responses mentioned above.

**Figure EV3:**
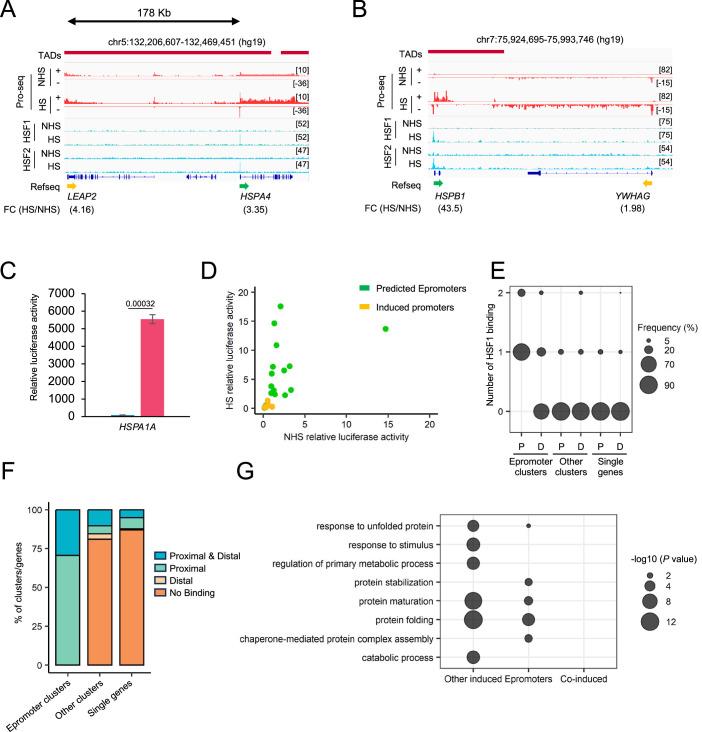
Heat shock response model from the “Vihervaara” dataset, related to Fig. 2. (**A**) Example of the *HSPA4* Epromoter-regulated cluster identified specifically by the pipeline using the TAD clustering method in the HS stress response. The genomic tracks show the PRO-seq signal (in red) and HSF1 and HSF2 ChIP-seq signal (in blue) before or after HS from the “Vihervaara” dataset. The topological associated domains (TAD) from K562 are at the top. The fold-change of induction is indicated below the name and orientation of the genes (Epromoter: green, co-induced genes: yellow). (**B**) Example of the *HSPB1* Epromoter-regulated cluster identified specifically by the pipeline using the distance clustering method in the HS stress response. The genomic tracks show the PRO-seq signal (in red) and HSF1 and HSF2 ChIP-seq signal (in blue) before or after HS from the “Vihervaara” dataset. The topological associated domains (TAD) from K562 are at the top. The fold-change of induction is indicated below the name and orientation of the genes (Epromoter: green, co-induced genes: yellow). (**C**) Luciferase assay to quantify the promoter activity of the control *HSPA1A* promoter before (blue) and after (red) HS in the K562 cells. The results were normalized on the Renilla activity. All data represent mean values ± SD of three biological replicates. *P* value was calculated by a paired one-sided Student’s t-test. (**D**) Scatterplot of the luciferase signal to quantify the enhancer activity of predicted Epromoter before (x-axis) and after (y-axis) HS. The predicted Epromoter (green) and the induced promoters (yellow) are highlighted. (**E**) Number of distal and proximal HSF1 binding associated with HS-induced genes for the indicated categories. (**F**) Percentage of clusters or single HS-induced genes associated with HSF1 binding at either proximal, distal or both genomic regions. (**G**) Significantly enriched GO biological processes for the HS-induced genes in K562, depending on their associated promoter. *P* value was calculated by an hypergeometric test using g:Profiler.

Initially, we compared the effectiveness of the two clustering methods using the “Vihervaara” dataset (Vihervaara et al, 2017) involving HS treatment in K562 cells (Fig. 2C). Analysis of 675 HS-induced genes using the distance-based method resulted in 75 clusters, harboring 168 genes. We distributed those clusters by the number of genes per cluster in function of the number of genes recruiting the HS TFs (here HSF1 and HSF2). We found 17 predicted Epromoter-regulated clusters (Fig. 2D, Table EV2). Using the TAD-based method resulted in 105 clusters, harboring 261 genes, and predicted 21 Epromoter-containing clusters. Comparison between the two methods resulted in 15 common clusters (Fig. 2E). The TAD-based method identified six additional clusters likely due to the lack of threshold between the TSS distances, while the two clusters uniquely identified by the distance-based method might be explained by the overlap of the cluster with the TAD borders (Fig. EV3A,B). Nevertheless, the two methods provided consistent identification of gene clusters potentially regulated by Epromoters. An example of an HS cluster potentially regulated by an Epromoter is provided by the *AP4B1-DCLREB1* Epromoter-regulated cluster, including two promoters for three induced genes (the predicted Epromoter is bidirectional) within the same TAD. Among the two promoters, only the *AP4B1-DCLREB1* promoter recruited HSF1 and HSF2, suggesting that the HS-induction of *HIPK1* is mediated by the *AP4B1-DCLREB1* Epromoter (Fig. 2F). For this analysis, we used TAD data from K562 cells without HS. Given that TAD data were not always available for the relevant cell lines and genome annotations and considering the potential need for stress-specific topological information, we focused, for the rest of the study, on clusters identified by the distance-based method.

To validate the efficiency of the pipeline in identifying Epromoter-regulated clusters, we tested the enhancer activity of 16 HS-induced Epromoters from the “Vihervaara” dataset by luciferase reporter assay in K562. As controls, we also tested 9 co-induced promoters. HS induction was confirmed by measuring the promoter activity of the HSPA1A promoter (Fig. EV3C). After transfection, cells were incubated for 1 h at 42 °C followed by 2 h of recovery at 37 °C. Out of the 16 predicted Epromoters, 14 exhibited enhancer activity (at least 2-fold induction), among them 10 Epromoters displayed significant induction of enhancer activity upon HS (Fig. 2G). In contrast, none of the 9 co-induced promoters showed enhancer activity in these assays. Overall, compared to the induced promoters, the Epromoters displayed significant HS-induced enhancer activity (Fig. 2G, insert, Fig. EV2D). The high proportion of experimentally validated HS-response Epromoters supports the efficiency of our strategy in identifying stress-response Epromoters.

Previous studies by Santiago et al, have shown that Epromoters in the IFNα response possess a higher density of TF binding sites (TFBS) specific to this stress (Santiago-Algarra et al, 2021). We sought to verify whether a high density of TFBS is also an intrinsic feature of Epromoters identified in the HS stress response. Using the JASPAR database, we calculated the number of HSF1 or HSF2 binding sites (also referred to as Heat shock Elements or HSEs) *per* HS-induced promoter (Fig. 2H). To compare the number of motifs between promoters, we divided the induced gene promoters into four categories: the predicted Epromoters, the predicted co-induced promoters (clustered with Epromoters), and all the other induced gene promoters, whether they recruit the TFs (HSF+) or not (HSF-). Additionally, we included a random set of promoters as a control for the background presence of HSE. We observed that Epromoters and HSF+ promoters displayed a higher density of HSE motifs compared to co-induced and HSF- promoters, respectively (Fig. 2I). For instance, 44% of Epromoters harbor 3 or more HSE motifs, while this is the case for only 18% of co-induced promoters. Strikingly, we also observed that co-induced promoters harbored fewer HSEs than other HS-induced promoters or random promoters, suggesting their depletion of HSEs. These observations support a model whereby the co-induced promoters are dependent on the binding of HSF1/HSF2 at Epromoters within the same cluster. On the same line, we investigated whether HS-induced genes might be regulated by distal *cis*-regulatory elements. For each cluster of induced genes, we calculated the number of proximal and distal HSF1 binding (Fig. EV3E). We found that 71% of Epromoter’s clusters were associated with proximal HSF1 binding, while the remaining 29% were associated with both proximal and distal HSF1 binding (Fig. EV3F), suggesting that some clusters might be regulated by both Epromoter and distal enhancer elements. Furthermore, we assessed the functional relevance of HS-induced genes (Fig. EV3G). As expected, we found that Epromoter-genes were enriched in Biological Process GO terms associated with heat shock, such as response to unfolded protein and protein stabilization and folding, similar to other induced genes, while co-induced genes displayed no significant enrichment.

### Systematic identification of stress-response clusters regulated by Epromoters

**Figure 3:**
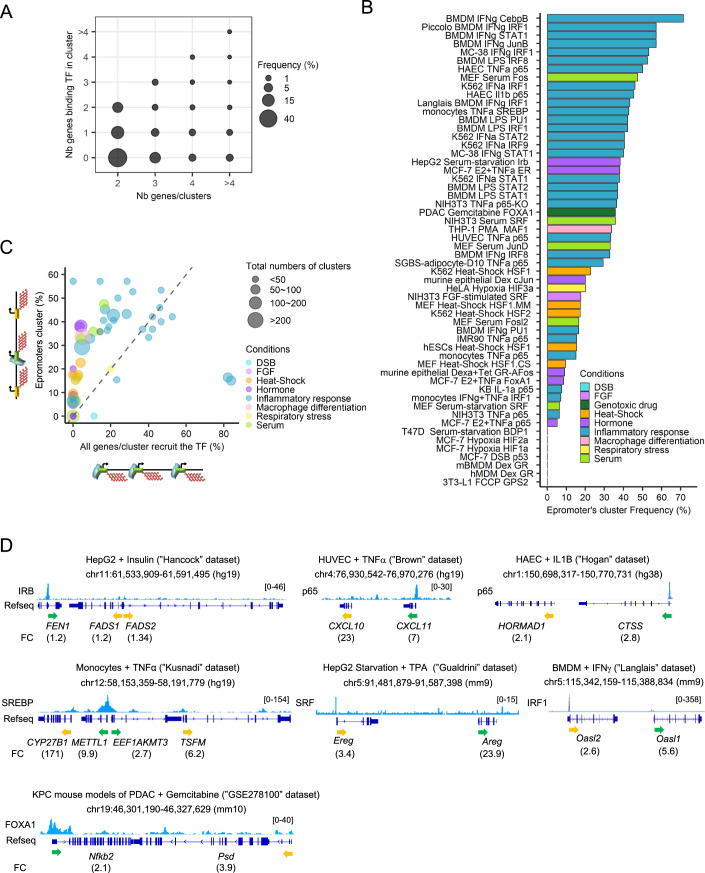
Frequency of Epromoter clusters in the different stress models. (**A**) Bubble plot showing the frequency of all the clusters identified in all the datasets by their number of genes (x-axis) compared to their number of genes recruiting their specific TFs (y-axis) determined by the pipeline. (**B**) Epromoter clusters frequency. Distribution of the Epromoter cluster frequency identified by the pipeline based on the distance for all the datasets. The colors indicated the different stimulation conditions of each dataset (light blue: double-strand breaks (DSB), light purple: fibroblast growth factors (FGF), dark green: genotoxic drug, orange: heat shock, purple: hormone, blue: inflammatory response, pink: macrophage differentiation, yellow: respiratory stress, light green: serum). (**C**) Distribution of the proportion of clusters where all clustered genes recruit the key TF (x-axis) compared to the proportion of Epromoters-regulated clusters (y-axis). The size of the bubble corresponds to the number of total identified clusters by the pipeline. The colors indicated the different stimulation conditions of each dataset (light blue: double-strand breaks (DSB), light purple: fibroblast growth factors (FGF), dark green: genotoxic drug, orange: heat shock, purple: hormone, blue: inflammatory response, pink: macrophage differentiation, yellow: respiratory stress, light green: serum). (**D**) Examples of Epromoter-regulated clusters identified by the pipeline in different stress conditions. The stress dataset is indicated at the top of each panel. The genomic tracks show the ChIP-seq signal of the TF used in each data after stimulation. The fold-change of induction is indicated below the name and orientation of the genes (Epromoter: green, co-induced genes: yellow). Source data are available online for this figure.

We applied our pipeline to systematically identify stress-response clusters regulated by Epromoters, using the 56 datasets described above (Table EV2; Dataset EV1). We identified a total of 728 non-redundant clusters potentially regulated by Epromoters. We distributed those clusters by the number of genes per cluster in function of the number of genes recruiting the associated TF (Fig. 3A). We found that amongst clusters harboring TF-bound genes, 62% had only one promoter bound by the TF and 72% had fewer promoter bounds by the TF than the total number of promoters within the cluster (Fig. 3A). On average, we found 18 Epromoter-regulated clusters per dataset (Table EV2). Out of 56 datasets, 49 contain at least one Epromoter cluster, ranging from 1 (“Biddie” (Biddie et al, 2011)) to 247 (“Phanstiel” dataset (Phanstiel et al, 2017)). To better assess the contribution of Epromoters to the regulation of stress-response clusters, we calculated the frequency of Epromoter-regulated clusters compared to all the clusters of induced genes found in each dataset (Fig. 3B). We found that Epromoter-predicted clusters represent between 0% and 71% of the identified clusters in each dataset, centered around 33% across all datasets. We then compared the proportion of Epromoter clusters to clusters where all genes recruit the identified key TFs to the stress (Fig. 3C). We observed that very few clusters contained all their genes recruiting the specific TFs, with a distribution centered at 10% of all clusters compared to 33% for Epromoter-like clusters. This points to a strong bias towards enrichment in stress-response clusters potentially regulated by Epromoters. Several examples of stress-response clusters potentially regulated by Epromoters are shown in Fig. 3D. Overall, our analyses suggested that regulation by Epromoters is a general mechanism in stress response rather than a specific or isolated phenomenon.

### Epromoters regulate gene clusters in different stress response models

**Figure 4:**
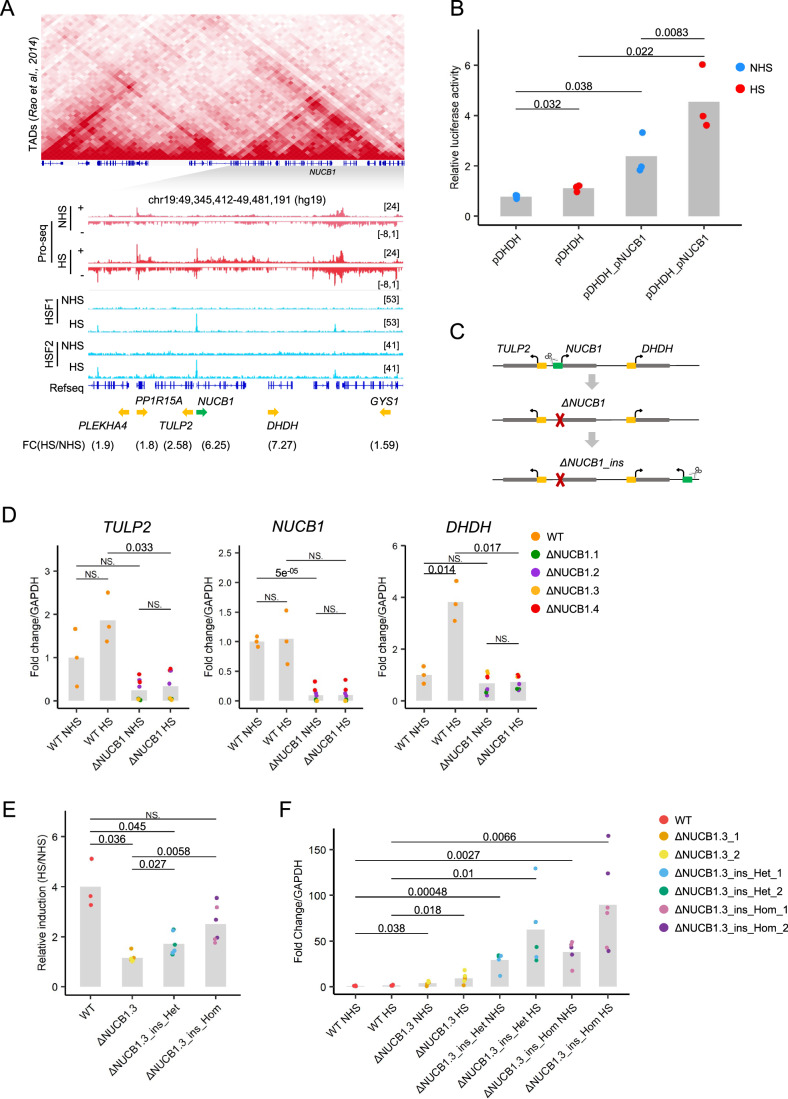
Epromoter-regulated cluster in the HS stress response. (**A**) The NUCB1 Epromoter-regulated cluster. (top) Hi-C triangular matrix (resolution 5 kb) of the locus from K562 cells. The genomic tracks show the PRO-seq signal (in red) and HSF1 and HSF2 ChIP-seq signal (in blue) before or after HS from the “Vihervaara” dataset. The fold-change of induction is indicated below the name and orientation of the genes (Epromoter: green, co-induced genes: yellow). (**B**) Luciferase assays to quantify the promoter activity of the induced *DHDH* promoter with or without the *NUCB1* Epromoter acting as an enhancer before and after HS in K562 cells. All data represent mean values of three biological replicates. *P* values were calculated by a paired one-sided Student’s t-test. (**C**) Schematic of the CRISPR-Cas9 genome deletion and re-insertion of the promoter region of the *NUCB1* Epromoter in K562 cells. (**D**) qPCR analysis of *TULP2*, *NUCB1*, and *DHDH* expression in wild-type and 4 ΔNUCB1 mutants in K562 cells before and after HS. Values represent the relative expression of the samples normalized to the housekeeping gene *GAPDH* and compared to the unstressed wild-type cells. The value for each biological replicate is shown. *P* values were calculated by a two-sided Student’s t-test. (**E**) qPCR analysis of the *DHDH* expression in wild-type K562 (WT), 2 ΔNUCB1 mutants, 2 heterozygote ΔNUCB1-inserted mutants, and 2 homozygote ΔNUCB1-inserted mutants in K562 cells. Values represent the relative expression of the samples normalized to the housekeeping gene *GAPDH*, then compared before and after HS. The value for each biological replicate is shown. *P* values were calculated by a two-sided Student’s t-test. (**F**) qPCR analysis of the promoter activity in wild-type K562 (WT), 2 ΔNUCB1 mutants, 2 heterozygote ΔNUCB1-inserted mutants, and 2 homozygote ΔNUCB1-inserted mutants in K562 cells before and after HS. Values represent the relative expression of the samples normalized to the housekeeping gene *GAPDH* and compared to the unstressed wild-type cells. The value for each biological replicate is shown. *P* values were calculated by a two-sided Student’s t-test. Source data are available online for this figure.

**Figure EV4:**
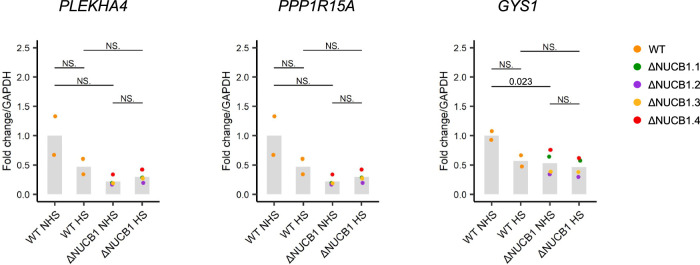
Gene expression of neighbor genes after deletion of the *NUCB1* Epromoter, related to Fig. 4. qPCR analysis of *PLEKHA4*, *PPP1R15A*, and *GYS1* expression in wild-type and 4 ΔNUCB1 mutants in K562 cells before and after HS. Values represent the relative expression of the samples normalized to the housekeeping gene *GAPDH* and compared to the unstressed wild-type cells. The value for each biological replicate is shown. *P* values were calculated by a two-sided Student’s t-test.

We aimed to assess the role of Epromoters predicted by our pipeline on the expression of distal genes from the same cluster in their endogenous locus using CRISPR/Cas9 genomic deletion. We started by studying the *NUCB1* Epromoter cluster, which consists of six genes induced by HS in K562 cells (“Vihervaara” dataset) (Fig. 4A). The *NUCB1* Epromoter is the only promoter of the cluster recruiting HSF1 and HSF2 TFs (Fig. 4A). Consistently, the *NUCB1* Epromoter displayed enhancer activity in luciferase assays, while the *DHDH* promoter, which is the highest induced gene of the cluster, did not (Fig. 2G). Moreover, the *NUCB1* Epromoter significantly induced *DHDH* promoter activity after HS treatment of K562 cells as assessed by luciferase assay (Fig. 4B). Next, we tested the Epromoter regulation in a cellular context by using CRISPR-Cas9 mediated genomic deletion of the *NUCB1* Epromoter in K562 cells (Fig. 4C). The knockout of the *NUCB1* Epromoter resulted in the loss of *NUCB1* expression and a lack of induction of neighboring genes *TULP2* and *DHDH* in four homozygous ΔNUCB1 clones (Fig. 4D), demonstrating the enhancer activity of this regulatory element in the endogenous context. Note that the expression of the other three genes of the cluster (*PPP15RA*, *PLEKHA4*, and *GYS1*) were not induced in our conditions, although their basal expression was slightly decreased in the knock-out cells (Fig. EV4). Therefore, the promoter sequence of *NUCB1* is crucial for the regulation of the HS-induced gene *DHDH*.

To test whether the *NUCB1* Epromoter can work as a distal regulatory element independently of its original genomic location, we re-inserted the *NUCB1* Epromoter sequence downstream of the *DHDH* gene in one of the ΔNUCB1 clones (Fig. 4C). We selected the site of the insertion downstream of *DHDH* to rule out any influence from transcripts coming from the *NUCB1* Epromoter. Moreover, the site of the insertion was located in a genome region deprived of active histone marks (H3K4me3 and H3K27ac) in K562, but inside the same TAD as *NUCB1* and *DHDH* to ensure the Epromoter remains within the same chromatin context. We obtained two homozygous and two heterozygous cell lines with the re-inserted Epromoter. Upon comparing the induction of *DHDH* after HS between these modified cells and non-inserted cells (Fig. 4E), we observed that *DHDH* induction was restored in cells with the re-inserted *NUCB1*-Epromoter and reached a similar fold change induction as observed in wild-type cells. We also measured the transcription downstream of the inserted sequence and observed HS-dependent transcriptional activity specifically in *NUCB1* re-inserted cells (Fig. 4F). This demonstrates that the *NUCB1* Epromoter sequence possesses intrinsic and location-independent enhancer and promoter activities.

**Figure 5:**
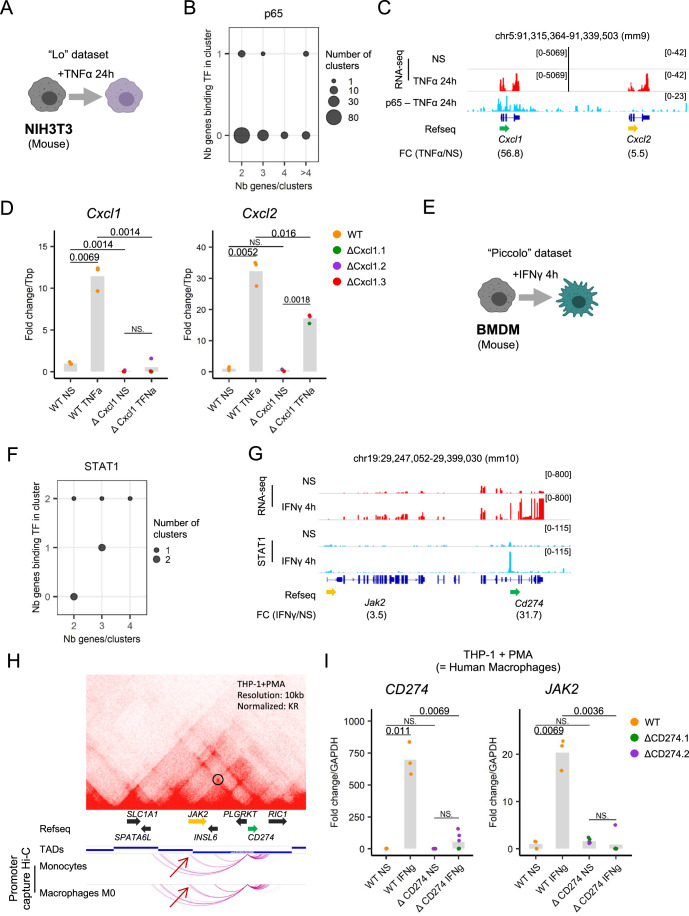
Epromoter-regulated cluster in the TNFα and the IFNγ stress response. (**A**) Schematic of the “Lo” dataset to induce TNFα response. (**B**) Bubble plot showing the number of all the clusters identified by their number of genes (x-axis) compared to their number of genes recruiting p65 (y-axis) determined by the pipeline. (**C**) Example of the *Cxcl1* Epromoter-regulated clusters identified by the pipeline in the TNFα stress response. The genomic tracks show the RNA-seq signal (in red) before or after 24 h of TNFα stimulation and the p65 ChIP-seq signal (in blue) after 24 h of TNFα stimulation from the “Lo” dataset. The fold-change of induction is indicated below the name and orientation of the genes (Epromoter: green, co-induced genes: yellow). (**D**) qPCR analysis of *Cxcl1*, *Cxcl2* expression in wild-type (orange) and 3 ΔCxcl1 mutants (green, purple, and red) in the mouse NIH3T3 cells before and after stimulation TNFα for 4 h. Values represent the relative expression of the samples normalized to the housekeeping gene *Tbp* and compared to the unstressed wild-type cells. The value for each biological replicate is shown. *P* values were calculated by a two-sided Student’s t-test. (**E**) Schematic of the experimental protocol from the “Piccolo” dataset to induce IFNγ response. (**F**) Bubble plot showing the number of all the clusters identified by their number of genes (x-axis) compared to their number of genes recruiting STAT1 (y-axis) determined by the pipeline. (**G**) Example of the *Cd274* Epromoter-regulated clusters identified by the pipeline in the IFNγ stress response. The genomic tracks show the RNA-seq signal (in red) and STAT1 ChIP-seq signal (in blue) before or after 4 h of TNFα stimulation from the “Piccolo” dataset. The fold-change of induction is indicated below the name and orientation of the genes (Epromoter: green, co-induced genes: yellow). (**H**) (top panel) Hi-C triangular matrix track (resolution at 10 kb, Knight-Ruiz (KR) normalized) and the TAD from THP-1 cells. (bottom panel) Genomic interaction detected by CHiCAGO at the *Cd274* promoter by Promoter Capture Hi-C in monocytes, in vitro differentiated macrophages (M0). (**I**) qPCR analysis of *PDL1*, *JAK2* expression in wild-type (orange) and 2 ΔPDL1 mutants (green and purple) in the human macrophages THP-1 cells with PMA before and after stimulation by IFNγ for 4 h. Values represent the relative expression of the samples normalized to the housekeeping gene *GAPDH* and compared to the unstressed wild-type cells. The value for each biological replicate is shown. *P* values were calculated by a two-sided Student’s t-test. Source data are available online for this figure.

In order to generalize our findings, we assessed the functional role of two additional predicted Epromoters induced by either TNFα or IFNγ cytokines. On the one hand, we used the “Lo” dataset (Lo et al, 2013), which includes RNA-seq and NF-kB (p65) ChIP-seq data, both before and after 24 h of TNFα stimulation in mouse NIH3T3 cells (Fig. 5A). Our pipeline identified 143 induced clusters, of which 9 were classified as Epromoter clusters, while none of the clusters harbor binding of p65 to all of the cluster-promoters (Fig. 5B, Table EV2). Among these, we selected the *Cxcl1* Epromoter cluster for further investigation. This cluster comprises the potential Epromoter *Cxcl1* and its potentially regulated gene, *Cxcl2* (Fig. 5C). Both genes code for proteins of the CXC chemokine family and are strongly induced during the TNFα response. However, only the *Cxcl1* Epromoter is bound by the p65 TF. To explore the functional activity of this predicted Epromoter, we employed CRISPR-Cas9 genome editing to delete the *Cxcl1* Epromoter, generating three knock-out clones in NIH3T3 cells. The gene expression of *Cxcl1* and *Cxcl2* was then assessed following 4 h of TNFα stimulation (Fig. 5D). The deletion of the *Cxcl1* promoter resulted in the complete loss of the *Cxcl1* expression but also in a significant reduction of the *Cxcl2* induction upon TNFα stimulation. These results indicate that the mouse *Cxcl1* promoter exhibits enhancer activity on its neighboring gene *Cxcl2* during the TNFα response. We note, however, that this regulation might be specific to the mouse, as we did not identify the *CXCL1-CXCL2* locus as a potential Epromoter-regulated cluster in human models of TNFα stimulation (Dataset EV1), which might be due to differences in locus configuration between the two species. Moreover, p65 binds the *CXCL2* promoter in human epithelial KB cells stimulated with IL-1a (Jurida et al, 2015), and is partially regulated by distal enhancers (Weiterer et al, 2020).

On the other hand, we took advantage of the pipeline to test the model based on data from primary cells. We chose the “Piccolo” dataset (Piccolo et al, 2017), which consisted of RNA-seq and STAT1 ChIP-seq data before and after 4 h of IFNγ stimulation in mouse Bone Marrow-Derived Macrophages (BMDM) primary cells (Fig. 5E). For instance, out of 110 distinct clusters, we identified 7 clusters potentially regulated by Epromoters (Fig. 5F, Table EV2). From the identified clusters, we chose to focus on the *Cd274*-Epromoter cluster. This cluster includes the *Cd274* gene, coding for the ligand of the PD1 inhibitory receptor (PD-L1), and the *Jak2* gene, coding for the tyrosine kinase JAK2. Strikingly, both genes play an essential role in the inflammatory response and are often co-regulated (Mineo et al, 2020; Sun et al, 2018), however, only the *CD274* Epromoter binds the STAT1 TF (Fig. 5G). To experimentally validate the role of the *CD274*-Epromoter in the IFNγ response, we used PMA-induced in vitro differentiated macrophages from the human THP-1 monocyte cell line, a classical model to study macrophage function. As expected, we observed that both *CD274* and *JAK2* genes were induced after 4 h of IFNγ stimulation of in vitro differentiated THP-1 macrophages. Hi-C data from macrophage-differentiated THP-1 cells (Phanstiel et al, 2017) revealed that both genes are in the same TAD. Consistently, analysis of Promoter Capture Hi-C interactions (Javierre et al, 2016) centered on the *CD274* promoter showed an interaction with the *JAK2* promoter which increased from monocytes to macrophage differentiation (Fig. 5H). Deletion of the *CD274* promoter in THP-1 cells resulted in significant impairment of both *CD274* and *JAK2* induction after 4 h of IFNγ stimulation in in vitro differentiated THP-1 macrophages (Fig. 5I). These findings indicate that the *CD274* promoter functions as an enhancer for *JAK2* in THP-1 differentiated cells. Overall, these results demonstrated the robustness of the pipeline in predicting bona fide Epromoters across various stress responses, and predictions from primary cells.

**Figure EV5:**
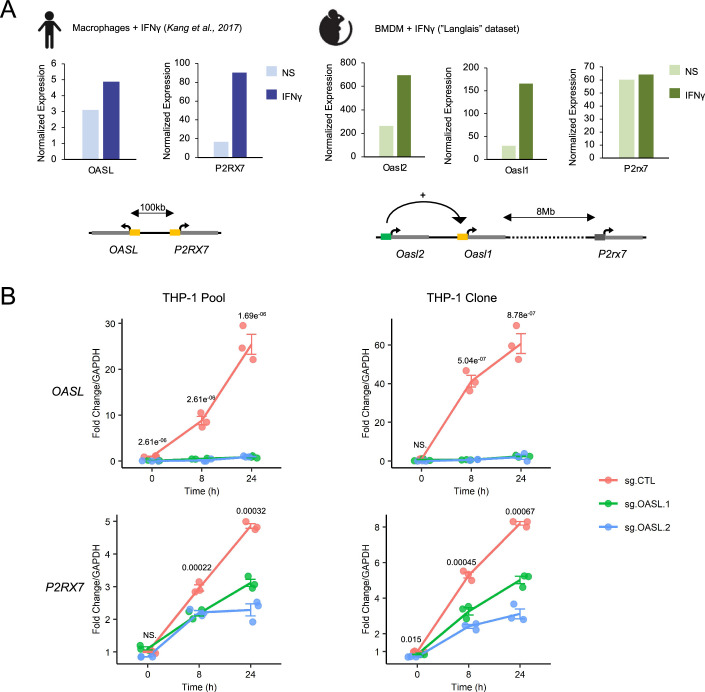
Regulation of *OASL/P2RX7* in mouse and human macrophages, related to Figs. 3 and 5. (**A**) (top panel) Mean normalized counts for *P2RX7/P2rx7* and *OASL/Oasl1/Oasl2* in unstimulated and IFNγ-stimulated human (blue) or mouse (green) macrophages. Data were retrieved from the “Langlais” dataset and expression data from (Kang et al, 2017). (bottom panel) Schematic representation of the locus in the human (left) and mouse (right). (**B**) qPCR analysis of *OASL* and *P2RX7* expression in dCas9-KRAB-MeCP2 THP-1, as a pool or as a clonal cell line, infected with either a negative control (sg.CTL) or two different single guide RNAs against *OASL* (sg.OASL.1 and sg.OASL.2). Values represent the relative expression of the samples normalized to the housekeeping gene *GAPDH*. All data represent mean values ± SD of three technical replicates. *P*-values for group differences (sg.CTL vs. sg.OASL) at each time point were calculated by one-way ANOVA with Benjamini–Hochberg correction.

Finally, we made use of large-scale structural variations across species to infer potential Epromoter regulation. Gilbertson et al (Gilbertson et al, 2022) described a chromosomal inversion of approximately 8 Mb on chromosome 5 between mice and humans. In mice, the genes *Oasl1* and *Oasl2* are separated by this 8 Mb region from the *P2rx7* gene, whereas in humans, the inversion brings the *OASL* gene (the ortholog of *Oasl1* and *Oasl2*) within 100 kb of *P2RX7*. This genomic rearrangement appears to have regulatory consequences as both *OASL* and *P2RX7* are co-induced by LPS stimulation in humans, while in mice, *Oasl1* and *Oasl2* are induced by LPS, but not *P2rx7* (Gilbertson et al, 2022). Interestingly, we identified *Oasl2* as an Epromoter for *Oasl1* in mouse macrophages in response to IFNγ (Langlais et al, 2016) (Fig. 3D). We noted that *P2rx7* is not induced in mice by IFNγ, while in human macrophages, both *OASL* and *P2RX7* are induced by IFNγ (Fig. EV5A; expression data from (Kang et al, 2017), Table EV1B). To assess the role of *OASL* promoter in the induction of *P2RX7*, we used a CRISPR inactivation system in the THP1 cell line (a model of human monocytes). We first observed consistent induction of both genes at 8 h and 24 h of IFNγ treatment in ThP1 cells (Fig. EV5B). We next generated THP1 cells stably expressing the dCas9-KRAB-MeCP2 cassette, either as a pool or a clonal cell line (see Methods section). Lentiviral expression of 2 different sgRNA targeting the *OASL* promoter completely abolished *OASL* expression as compared to a control sgRNA (Fig. EV5B). Strikingly, the two *OASL* sgRNAs also significantly reduced the induction of *P2RX7* after IFNγ treatment, suggesting that the *OASL*-Epromoter contributes to the *cis*-regulation of *P2RX7* in human cells. These results likely reflect the co-optation of *P2RX7* into the inflammatory response by hijacking the *OASL* Epromoter in humans.

### Disruption of Epromoter-bound SRF TF impact on the expression of the whole clusters

**Figure 6:**
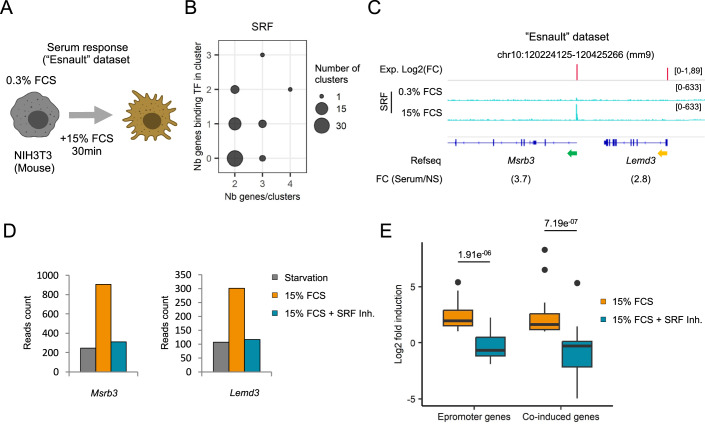
TF disruption at Epromoter-regulated clusters in the serum response. (**A**) Schematic of the “Esnault” dataset to induce serum response (15% FCS) after serum starvation (0.3% FCS). (**B**) Bubble plot showing the number of all the clusters identified by their number of genes (x-axis) compared to their number of genes recruiting SRF (y-axis) determined by the pipeline. (**C**) Example of the *Msrb3* Epromoter-regulated clusters identified by the pipeline in the serum stress response. The genomic tracks show the log2 fold-change of the RNA-seq signal (in red) and SRF ChIP-seq signal (in blue) before or after serum stimulation from the “Esnault” dataset. The fold-change of induction is indicated below the name and orientation of the genes (Epromoter: green, co-induced genes: yellow). (**D**) RNA-seq reads count of the genes *Msrb3* and *Lemd3* after starvation (gray, FCS Starvation), stimulated by serum (orange, FCS) or stimulated by serum with inhibited SRF (blue, FCS + SRF inh). (**E**) Boxplot of the log2-transformed fold of induction of the Epromoters (*n* = 20) and co-induced genes (*n* = 25) identified by the pipeline after FCS stimulation (orange) or stimulation with inhibited SRF (blue). *P* value was calculated by a paired two-sided Wilcoxon test. Central values represent the median of the signal, the interquartile range (IQR) corresponds to the 75th to 25th percentile, and whiskers extend to the maximum and minimum values excluding outliers. Source data are available online for this figure.

We observed that for Epromoter-regulated clusters, only one of the promoters is bound by the key TF that is required for the stress response. This implies that disruption of this TF should impact the expression of all the genes in the cluster, therefore reflecting the regulatory role of the Epromoter. To address this question, we used the “Esnault” dataset (Esnault et al, 2014) of NIH3T3 mouse fibroblasts upon 30 min of serum stimulation after starvation (Fig. 6A). Serum response induced a rapid gene induction, which is mainly dependent on the binding of the Serum Response Factor (SRF) (Esnault et al, 2014; Gualdrini et al, 2016; Onuh and Qiu, 2021). The pipeline identified 56 clusters, of which 20 were classified as Epromoter clusters (Fig. 6B; Table EV2). Additionally, the study included RNA-seq data in conditions of SRF inhibition. Examination of the *Msrb3* Epromoter-like cluster (Fig. 6C) revealed that SRF inhibition abolished the induction of the *Msrb3* gene and the *Lemd3* co-induced gene located in the same cluster (Fig. 6D), suggesting that SRF binding on the *Msrb3* Epromoter is also required for the induction of the *Lemd3* gene. To generalize this observation, we quantified the effect of SRF inhibition on the serum induction of all Epromoter-genes and co-induced genes. As shown in Fig. 6E, the induction of both sets of genes was similarly affected by SRF inhibition. This finding supports a dependence of co-induced genes on Epromoters for TF recruitment and regulatory function.

## Discussion

**Figure EV6:**
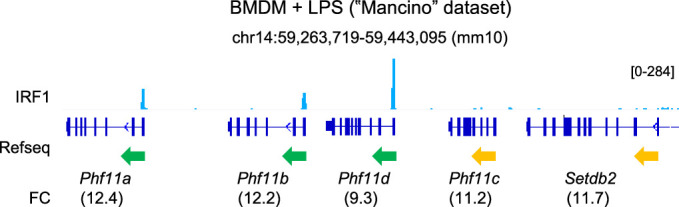
Screenshot of the *Phf11* cluster by LPS stimulation from the “Mancino” dataset, related to the discussion. The genomic tracks show the ChIP-seq signal of IRF1 after stimulation. The fold-change of induction is indicated below the name and orientation of the genes (Epromoter: green, co-induced genes: yellow). Note that all four *Phf11* and *Setdb2* genes are induced, but all except *Phf11c* and *Setdb2* bind IRF1.

To better understand the regulation of stress-response genes, we analyzed a series of published datasets from different proteotoxic, environmental and inflammatory stresses, including gene expression data and ChIP-seq for key transcription factors involved in the same stress responses. We initially found that stress-response genes are frequently located in close genome proximity, pointing to shared regulatory networks. We then aimed to assess the implications of Epromoters in the regulation of stress-response clusters. We previously showed that IFNα-induced Epromoters preferentially recruit the key interferon response complex (STAT1/STAT2, IRF1/IRF9) and regulate the interferon response of neighboring genes (Santiago-Algarra et al, 2021). Leveraging these findings, we developed a pipeline whereby stress-response clusters potentially regulated by Epromoters were some, but not all, the induced promoters present in the cluster recruited the key TF involved in the regulation of the stress response. We frequently found Epromoter-regulated clusters where only one promoter recruits the key TF (Dataset EV1). However, more complex situations also exist where only a limited number of promoters do not recruit the TF (e.g., *Phf11* cluster; Fig. EV6). For instance, we previously identified the IFIT cluster composed of a family of 4 IFNα-induced genes with 3 recruiting the STAT1/2 and IRF1/9 factors (Santiago-Algarra et al, 2021). The deletion of one of the promoters (*IFIT3*) inhibited the induction of the fourth gene (*IFIT2*).

We observed frequent stress-response clusters potentially regulated by Epromoters in the majority of the studied stress models. We systematically validated the predicted enhancer activity of HS-induced Epromoters and confirmed the regulatory role of Epromoters on stress-induced clusters within their endogenous loci, across three different stress conditions and cell types. Furthermore, using the HS-inducible *NUCB1-DHDH* loci we demonstrated that relocating an Epromoter within its genomic locus did not affect its function, proving that the Epromoter sequence is sufficient to function independently of its original genomic position. Overall, our findings indicate that Epromoters-regulated clusters are a general mechanism of stress response where Epromoters specifically recruit key stress-associated TFs, thereby facilitating the rapid and coordinated activation of genes within such clusters to have a more efficient response to intra- and extracellular stress signaling. These findings have significant implications for understanding complex gene regulation following the response to acute perturbations.

One limitation of our study regards the potential underestimation of stress-response clusters regulated by Epromoters. On the one hand, our current pipeline identified clusters with a threshold of 100 kb between the TSS of co-induced genes. More optimal approaches might use 3D interaction data or an adaptive threshold distance specific to each stress condition. On the other hand, our identification of Epromoter clusters was also limited by the availability of stress-related datasets. Implementation of our pipeline into a user-friendly web tool (https://epromoters.tagc.univ-amu.fr) will ease the systematic identification of new Epromoters clusters in the future.

The impact on neighbor gene expression after deletion of the Epromoter could, in theory, be explained by alternative mechanisms, including the regulatory function of the gene product and readthrough effects. In previous studies, we have shown that the re-expression of Epromoter-associated genes did not rescue the expression of neighbor genes (Dao et al, 2017; Santiago-Algarra et al, 2021), indicating direct regulation of neighboring gene expression. Various types of stress, such as high temperatures and viral infections, can induce Pol II transcription past the normal termination site into neighboring genes, a process termed readthrough transcription (Pessa et al, 2024). However, this mechanism will only work when the neighbor gene is located downstream of the stress-response gene in a tail-to-head configuration. Anyhow, the rescue of *DHDH* induction observed after repositioning of the HS-Epromoter *NUCB1* is not compatible with the two aforementioned mechanisms and rather supports a direct regulatory role of Epromoters.

Stress response is a cellular mechanism that allows cells to adapt to various environmental disturbances that disrupt their homeostasis. Numerous factors can be considered as stress factors. The most well-known stressors include DNA-damaging agents, nutrient deprivation, temperature fluctuations, chemical toxins, and infectious agents. Each type of stress triggers a specific response that includes rapid changes in gene transcription. Even though the transcription landscape can be greatly modified, such changes are mostly mediated by a few and very specific TFs. The implications of a small number of TFs upon stress allow the cells to quickly respond and adapt to it but also to swiftly return to normal function once the stress is alleviated. Previous studies suggested that Epromoters might be involved in stress response and in particular, the interferon response (Dao et al, 2017; Dao and Spicuglia, 2018; Muerdter et al, 2018; Santiago-Algarra et al, 2021). For instance, active Epromoters in HeLa cells, which have a constitutive type 1 interferon response, are significantly associated with interferon response (Dao et al, 2017). Indeed, inhibition of type I interferon in HeLa cells preferentially affects the activity of Epromoters as compared with distal enhancers (Dao and Spicuglia, 2018; Muerdter et al, 2018; Santiago-Algarra et al, 2021). Moreover, using high-throughput reporter assays, we previously showed that IFNα-induced genes are frequently organized in clusters regulated by a single Epromoter (Santiago-Algarra et al, 2021). Within these clusters, Epromoters function as regulatory hubs that selectively recruit TFs essential for IFNα-driven responses, coordinating gene expression across the cluster. Our current study validates and extends these findings by demonstrating a predominant role of Epromoters in the regulation of stress-response genes in general.

Cell identity genes are known to be regulated by distal enhancers, often organized in clusters of enhancers in large intergenic regions (Field and Adelman, 2020). In contrast, housekeeping genes lack interactions with distal enhancers but are regulated by promoter-promoter interactions mediated by the binding of the Ronin TF (Dejosez et al, 2023). Here, we propose that stress-response genes are preferentially organized in clusters in which often one of the promoters plays a primordial role in the coordination of all genes of the cluster. Thus, our findings demonstrate that clustering of gene regulatory elements, whether enhancers or promoters, is a general principle of gene regulation that is not limited to cell identity or housekeeping genes but extends to stress and inflammatory response genes that are essential for cellular homeostasis.

It is clear that not all stress-response genes are found in clusters. While unclustered genes are more likely to be regulated by typical enhancers, our results suggest that clustered genes are preferentially regulated by Epromoters. This is supported by our previous study showing that unclustered interferon-induced genes are associated with distal regulatory elements binding the ISGF3 complex, while those found within clusters are more frequently associated with ISGF3-bound Epromoters (Santiago-Algarra et al, 2021). Moreover, it is likely that the propensity to be regulated by Epromoters might depend on the affinity of TFs to preferentially bind proximal or distal regulatory elements. For instance, while HSF1/2 factors preferentially bind promoter regions in response to heat shock (Vihervaara et al, 2017), in the proinflammatory response, regulation often finds p65 bound to both the enhancers and the promoters of responsive genes (Kolovos et al, 2016) or a given master enhancer controlling several genes in a cluster (Jurida et al, 2015; Weiterer et al, 2020).

Features defining the enhancer *versus* promoter activity of regulatory elements are a fundamental question in the gene regulation field and a focus of extensive research (Andersson and Sandelin, 2020; Catarino et al, 2017; Core et al, 2014; Field and Adelman, 2020; Henriques et al, 2018; Malfait et al, 2023; Mikhaylichenko et al, 2018; Nguyen et al, 2016; Rennie et al, 2018). In this context, what are the mechanistic bases leading to the enhancer activity of stress-response Epromoters? We previously showed that constitutive Epromoters bind a higher number of TFs and harbor a more complex combination of TF binding sites as compared with classical promoters (Dao et al, 2017), suggesting that one of the potential mechanisms mediating enhancer function might be the efficient recruitment of key TFs. Here, we observed that HS-induced Epromoters harbor a higher density of HSF1/2 motifs as compared with other induced promoters, which could explain the highly efficient recruitment of these TFs. Similarly, the density and quality of interferon-response elements are higher at interferon-induced Epromoters as compared to the promoters of other induced genes, but similar to the predicted distal enhancers (Santiago-Algarra et al, 2021). This is therefore likely that a high density of stress-response binding sites can lead to increased efficiency of TF recruitment and/or stabilization required for the enhancer function of Epromoters.

The regulation of stress-response clusters by Epromoter aligns well with the notion of inducible transcriptional condensates defining discrete membrane-less sub-nuclear compartments containing a high concentration of RNA polymerase and key TFs and where efficient transcription can be triggered (Cook and Marenduzzo, 2018; Feuerborn and Cook, 2015; Pessa et al, 2024; Rippe and Papantonis, 2025; Seufert et al, 2024). A striking example is provided by NF-κB-regulated genes in response to TNFα stimulation (Fanucchi et al, 2013). Experimental removal of a gene from the NF-κB-dependent multi-gene complex was shown to directly affect the transcription of its interacting genes, suggesting that co-association of co-regulated promoters might contribute to a hierarchy of gene expression control. Similarly, Epromoters might likely tend to generate small, localized hubs due to their higher recruitment of TFs. In this context, the highly efficient recruitment of stress-response factors observed at Epromoters could lead to the formation of condensates through the interaction of TF’s intrinsic disordered regions, as seen with HSF1 (Chowdhary et al, 2022) and RNA Pol II (Bhat et al, 2021). This, in turn, could either facilitate the assembly or maintenance of stress condensates by tightening promoter-promoter interactions or bringing specific transcriptional regulators required for the regulation of the neighbor genes. In support of this hypothesis, we found that the *CD274* and *JAK2* co-regulated genes are within the same interacting loop in unstimulated macrophages, but the interaction is increased upon IFNγ stimulation, in agreement with previous results suggesting that a pre-establish loop between *cis*-regulatory elements can facilitate rapid gene induction (Cuartero et al, 2018; Jin et al, 2013). This would be particularly relevant in the case of rapid and coordinated regulation of gene expression in response to environmental or intrinsic cellular stimuli (Pessa et al, 2024). Our work, thus, provides a framework for future studies aiming to address the contribution of Epromoters in the 3D organization of the genome and the formation or stabilization of transcriptional condensates in response to different stress signaling.

Our findings have significant implications for the understanding of the evolution of regulatory elements and stress-response clusters. On the one hand, many of the identified stress-response clusters are likely to arise through gene duplication events. Indeed, many of the identified clusters consist of genes from the same gene family, including, for example, *OAS, IFIT*, *CXCL*, *CXCR* and *GBP* clusters. For instance, gene expansion has been shown to significantly contribute to the evolution of the IFN system and suggested to confer a selective advantage to the host species (Shaw et al, 2017). It is possible that during the duplication of stress-response genes, ancestral promoters’ elements have acquired enhancer functions to coordinate the response of the new gene isoforms within the cluster. On the other hand, recent works suggested that repurposing of promoters and enhancers facilitated regulatory innovation and the origination of new genes during evolution (Andersson, 2015; Arenas-Mena, 2017; Carelli et al, 2018; Ghanbarian and Hurst, 2015; Majic and Payne, 2020; Mikhaylichenko et al, 2018; Wu and Sharp, 2013; Xie et al, 2012). Similarly, the proximity to an Epromoter-associated locus might provide a rapid co-option for neighbor genes to acquire new functions in stress response. Indeed, we observed that for HS clusters, Epromoter genes were directly associated with HS-related functions, while no significant enrichment was found for the distal Epromoter-regulated genes. These observations are reminiscent of previous findings that rapid induction of immediate-early genes in response to growth factor stimulation is accompanied by the co-upregulation of their neighboring genes (Ebisuya et al, 2008). These observations suggest that transcriptional activation has a ripple effect, which may be advantageous for coordinated expression. It is plausible that these genes, while initially unrelated to the stress responses, may have been co-opted into stress-related pathways by Epromoter hijacking, as suggested by the regulation of *P2RX7* induction by the *OASL* Epromoter after proximal genomic relocation in humans (Fig. EV5). Alternatively, it is equally possible that some of the distally co-regulated genes might be induced by the proximity to the Epromoters without any physiological relevance. Future work will need to be performed to more systematically assess the functional role of the distal-associated genes.

As dysregulation of stress response signaling pathways is involved in multiple pathologies, it is likely that genetic or epigenetic alterations of stress-Epromoters might have a pleiotropic role in the etiology of inflammatory- and stress-related diseases (Netea et al, 2017; Pessa et al, 2024). Several studies, including ours, have demonstrated that human genetic variation within Epromoters influences distal gene expression (Dao et al, 2017; Jung et al, 2019; Mitchelmore et al, 2020; Saint Just Ribeiro et al, 2022; Wang et al, 2018). Moreover, specific examples highlight the distal impact of disease-associated variants within Epromoters (Chandra et al, 2021; Gao et al, 2018; Hua et al, 2018; Malfait et al, 2023; Nisar et al, 2022; Rusu et al, 2017; Sergeeva et al, 2016; Yagihara et al, 2016). As Epromoters potentially control several genes at the same time and efficiently recruit key TFs, mutations in these regulatory elements are expected to have a stronger pathological impact, as compared to typical promoters. This might result from the regulation of multiple genes either involved in the same (additive or synergistic effects) or different (pleiotropy) pathways. Future work will require a systematic association of Epromoters with genetic traits, coupled with functional studies of the target genes in order to demonstrate their involvement in the context of stress-related disease.

### Conclusions

By leveraging extensive genomic and functional datasets, our study explores the relationship between Epromoter and the regulation of stress-response clusters, shedding light on the complex regulatory mechanisms leading to rapid gene induction during the cellular responses to intra- and extracellular stress signaling. Given the involvement of stress response in pathological conditions and the proposed pleiotropic role of Epromoters (Malfait et al, 2023), we foresee an important contribution of this mechanism to disease.

## Methods

**Table:** Reagents and tools table

Reagent/Resource	Reference or Source	Identifier or Catalog Number
**Experimental models**		
K562 (human chronic myelogenous leukemia cell line)	ATCC	CCL-243
NIH3T3 (mouse embryonic fibroblast cell line)	ATCC	CRL-1658
THP-1 (human acute monocytic leukemia cell line)	DSMZ	ACC 16
HEK293T cells	ATCC	CRL-3216
NEB® 5-alpha Competent E. coli	New England Biolabs	C2987
Endura E. coli cells	Lucigen	60241-2
**Antibodies**		
**Oligonucleotides and other sequence-based reagents**		
pGL4.12 vector	Promega	#E6671
dCas9–KRAB–MeCP2 lentiviral vector	Addgene	#122205
Crop-seq-derived lentivirus (sgRNA cloning vector)	Addgene	#86708
qPCR primers	This study	Table SV3
Crispr Guide RNA	This study	Table SV3
Luciferase assay plasmids	This study	Table SV3
Alt-R® CRISPR-Cas9 tracrRNA	Integrated DNA Technologies	1072533
**Chemicals, Enzymes and other reagents**		
RPMI 1640 media + Glutamax	Thermo Fisher Scientific	61870-044
DMEM media	Thermo Fisher Scientific	11960-085
0.05% Trypsin-EDTA (1X)	Thermo Fisher Scientific	25300-104
Fetal Bovine Serum (FBS)	Thermo Fisher Scientific	A5253701
DPBS (1X)	Thermo Fisher Scientific	14190-169
TNFα	Abcam	ab9642
IFNγ	Miltenyi Biotec	130-096-484
PMA (phorbol 12-myristate 13-acetate)	Sigma-Aldrich	P1585
Penicillin–Streptomycin–Glutamine	Thermo Fisher Scientific	10378016
Dual-Luciferase kit	Promega	E1980
BamHI-HF	New England Biolabs	R3136S
SalI-HF	New England Biolabs	R3138S
BglII	New England Biolabs	R0144S
HindIII	New England Biolabs	R0104S
Alt-R CRISPR-Cas9 System	Integrated DNA Technologies	075915
Alt-R HDR Enhancer V2	Integrated DNA Technologies	10007910
Alt-R™-s.p Cas9 Nuclease V3	Integrated DNA Technologies	1081059
CRISPRIMAX lipofectamine	Invitrogen	CMAX00001
RNeasy Plus Mini Kit	Qiagen	74106
Phire Tissue Direct PCR Master Mix	Thermo Fisher Scientific	F170L
SuperScript VILO	Thermo Fisher Scientific	1755250
SYBR Green Master Mix	Thermo Fisher Scientific	4367660
Guide-it Long ssDNA Production System v2	Takara	632666
Nucleo Spin Gel and PCR clean-up	Macherey-Nagel	740609.250
NucloBond Xtra Midi	Macherey-Nagel	740410.100
**Software**		
Epromoter-like-cluster pipeline	https://github.com/Spicuglia-Lab/Epromoter-like-cluster-pipeline	
RStudio	Posit	2022.02.0 + 443
R package ggplot2 v3.4.1	https://ggplot2.tidyverse.org/	
R package rrvgo	https://www.bioconductor.org/packages/release/bioc/html/rrvgo.html	
New WashU Epigenome Browser	https://epigenomegateway.wustl.edu/	
Custom Alt-R™ CRISPR-cas9 guide RNA	Integrated DNA technologies_ https://eu.idtdna.com/site/order/designtool/index/CRISPR_CUSTOM	
CrispRGold	https://crisprgold.mdc-berlin.de	
Primer3web version 4.1.0	https://primer3.ut.ee/	
SerialCloner 2-6-1	http://serialbasics.free.fr/Serial_Cloner.html	
IGV_2.8.10	https://igv.org/	
SnapGene Viewer 5.0.7	https://www.snapgene.com/	
**Other**		
QuantStudio 6 Flex Real-Time PCR System	Applied Biosystems	4484642
Neon Transfection System	Thermo Fisher Scientific	MPK10096B
Victore Nivo	PerkinElmer	
DS-11FX Spectrophotometer/Fluometer	DeNovix	

### Methods and protocols

#### Selection of stress-related datasets

Selection of stress-related datasets was achieved by manual inspection of the literature. We selected mouse and human datasets for which differential gene expression in any stress conditions and ChIP-seq for key TFs involved in the same stress conditions were available. All stress-related datasets used in this study are listed in Table EV1 and described in Table EV2.

### Distance distribution of induced genes

Induced genes were identified from gene expression data derived from stress response and cell differentiation datasets (Table EV1), considering only protein-coding genes. Reference gene annotation files were downloaded from the UCSC Genome Browser: human (hg19: https://hgdownload.soe.ucsc.edu/goldenPath/hg19/database/refGene.txt.gz, hg38: http://hgdownload.soe.ucsc.edu/goldenPath/hg38/database/refGene.txt.gz), and mouse (mm9: https://hgdownload.soe.ucsc.edu/goldenPath/mm9/database/refGene.txt.gz, mm10: https://hgdownload.soe.ucsc.edu/goldenPath/mm10/database/refGene.txt.gz). The distances between the transcription start sites (TSS) of induced genes were calculated using Bedtools with the following parameters: bedtools closest -a $file -b $file -d -io -t first. For comparison, random gene sets containing the same number of genes as the induced gene sets were generated by randomly selecting protein-coding genes from the annotation files. For the “Vihervaara” and “Park” datasets, we also analyzed a control list of non-induced genes (FC < 1). The distances between the TSS of these control genes were calculated in the same manner as for the induced genes. To assess statistical significance in the distance distributions between induced and control genes, a Kolmogorov–Smirnov (K-S) test was performed. To quantify the deviation between the distance distributions of induced and control genes, deviation scores were defined using a Q-Q plot-based method. Specifically, the Euclidean distances between the observed induced-control points (divided into 1000 quantiles) and the expected line of equality (y = x, representing no difference between distributions) were calculated. The sum of these Euclidean distances was defined as the deviation score.

### Identification of Epromoter-regulated clusters

We developed a pipeline to identify Epromoter-regulated clusters using expression data (RNA-seq, Pro-seq or microarrays) and ChIP-seq for key transcription factors involved in the same stress responses. The pipeline and its’ usage can be accessed from Github: https://github.com/Spicuglia-Lab/Epromoter-like-cluster-pipeline or directly implemented using the web-based tool: https://epromoters.tagc.univ-amu.fr. The pipeline identifies clusters of induced genes in stimulated conditions on the basis of (i) the distance between two induced gene’s transcription start sites (TSS) must be less than 100 kb, and (ii) genes per cluster should be more than 1. TF binding peaks overlapping with the TSS-proximal regions (±1 kb) are considered as a TF binding to the promoter of the gene, including alternative TSSs. We consider a cluster regulated by Epromoter(s) when the number of induced genes is higher than the number of genes where the promoter is bound by the TF. For each execution of the pipeline, it takes differentially expressed genes and TFs binding peaks as input. The input data requires three files: differentially expressed genes in stimulated condition with fold change, TF binding peaks in the same condition, and reference transcript annotation file. The output of the pipeline includes two files: (1) gene clusters with TF binding information and (2) Epromoter-regulated clusters. Additionally, the pipeline provided a TAD (Topologically associating domain) clustering method which considers induced genes working as clusters in a TAD. TAD clusters where only one promoter was bound by the TF were selected as Epromoter-regulated clusters. The pipeline was coded with Python 3.7.12 and implemented in a Linux environment. The dependent libraries include pandas, plotnine, numpy, subprocess, seaborn, itertools, matplotlib, scipy, pybedtools. The list of Epromoter-regulated clusters for all datasets is provided in the Dataset EV1.

### Cell culture

Cell line K562 (CCL-243), a chronic myelogenous leukemia cell line, was obtained from the ATCC (American Type Culture Collection) and maintained in RPMI 1640 media (Thermo Fischer Scientific, 61870-044) supplemented with 10% FBS (Thermo Fischer Scientific, A5253701) at 37 °C and 5% CO_2_. Cells were passed every 3 days at 2 × 10^5^ cells/mL and routinely tested for mycoplasma contamination. Cell line NIH3T3 (CRL-1658), an embryonic mouse fibroblast, and maintained in DMEM media (Thermo Fisher Scientific,11960-085) supplemented with 10% FBS (Thermo Fischer Scientific, A5253701) at 37 °C and 5% CO_2_. Cells were passaged every 2 days at 2 × 10^5^ cells/mL and routinely tested for mycoplasma contamination. THP-1, a human acute monocytic leukemia cell line, was obtained from DSMZ (ACC 16). Cells were grown in RPMI 1640 media (Thermo Fischer Scientific) supplemented with 10% FBS (Thermo Fischer Scientific) at 37 °C and 5% CO_2_. Cells were passaged every 3 days at 10^6^ cells/mL and routinely tested for mycoplasma contamination.

### Stress conditions

#### Heat-shock treatment

Exponentially growing K562 cells were placed in water baths at 37 °C (NHS) or at 42 °C (HS) for 1 h followed by a recovery step of 2 h in an incubator at 37 °C and 5% CO_2_.

#### TNFα treatment

Exponentially growing NIH3T3 cells were stimulated by adding one volume of fresh media containing 10 ng/mL TNFα (Abcam, ab9642), for a final concentration of 5 ng/mL, during 4 h.

#### IFNγ treatment

To induce macrophage differentiation, THP-1 cells were first plated on 6-well plates (2 × 10^6^ cells/well), in media containing 10 ng/mL phorbol 12-myristate 13-acetate (PMA; Sigma-Aldrich, P1585) for 48 h. After 48 h of incubation, the PMA-containing media was replaced with fresh media, and cells were incubated for an additional 24 h. THP-1 in vitro differentiated macrophages were then stimulated by replacing media with 2 mL of fresh media containing 100 ng/mL of IFNγ (Miltenyi Biotec, 130-096-484) for 4 h, upon which the extraction of RNA was performed. For each group, three independent simulations were made.

### Luciferase reporter assay

The pGL4-SV40 vector was created by cloning the promoter SV40 into the pGL4.12 vector (Promega, #E6671) at the BglII and HindIII restriction sites. The human promoters (500 bp) tested were cloned into the pGL4-SV40 vector downstream of the luciferase gene at the SalI and BamHI restriction sites (Table EV3A). The human promoters *HSPA1A* and *DHDH* were cloned into the pGL4.12 vector (Promega, #E6671) at the BglII and HindIII restriction sites. The human promoter *DHDH* was also cloned at the BglII and HindIII restriction sites instead of the SV40 promoter in the pGL4-SV40-NUCB1 vector (Table EV3A). For cell transfection, 1 × 10^6^ K562 cells were mixed with 1 μg of each construct and 125 ng of Renilla using the Neon™ Transfection System (Thermo Fisher Scientific; 100 µl tips; pulse voltage 1450 V, pulse width 10, pulse number 3) and cultured in 1.2 mL in 12-well plates. For heat shock treatment, 24 h after transfection cells were placed in water baths at 37 °C (NHS) or at 42 °C (HS) for 1 h followed by a recovery step of 2 h in an incubator at 37 °C and 5% CO_2_. The Dual-Luciferase kit (Promega, E1980) was used to measure luciferase and Renilla luminescence following the manufacturer’s recommendations. Data were normalized to Renilla values and represented as the fold-change of relative light units over the pSV40-luc vector. Experiments were performed in triplicate.

### Motifs enrichment analysis

The HSF1 and HSF2 binding sites in the human genome were recovered from the JASPAR database (MA0486.2 for HSF1 and MA0770.1 for HSF2) (Sandelin, 2004) and overlapped with the promoter of all the induced genes induced by the HS response according to the PRO-seq data from the “Vihervaara” dataset or random promoters. The promoter coordinates were extended from −1250 bp to +750 bp centered on the TSS. The overlap was accomplished by using the function intersect of bedtools with the overlaps count (-c) between the coordinates of the HSF1/HSF2 JASPAR motifs and the coordinates of all the induced genes.

### HSF1 binding in function of the genomic location

Analyses were performed using bedtools (v2.31.1). Clusters were extended by ±100 kb using pybedtools (slop function) and the TSS coordinates from the most upstream and downstream genes of the cluster. For single induced genes, the most upstream TSS was used to define a genomic region of ±100 kb. HSF1 ChIP-seq peaks were classified as proximal (within 1 kb of a TSS) or intergenic. Overlaps between peaks and promoter clusters or single induced genes were quantified with bedtools annotate -counts, which directly reports the number of overlaps for each category.

### Functional enrichment

GO enrichment in biological processes was assessed using g:Profiler (Reimand et al, 2016) and default options. For the statistical background, we used the list of all genes associated with the induced promoters. Enrichment scores were calculated using the g:GOSt native method. The R package “rrvgo” was used with default options to concatenate the GO biological process term (Sayols, 2023).

### CRISPR/Cas9 genome deletion

For promoter deletion, we used the web tool CrispRGold and the CRISPR-Cas9 guide RNA from IDT to design two guide RNAs flanking the Epromoter regions. For each Epromoter, two primers were designed flanking the target region that allows us to identify the wild-type and the mutant alleles. We used the Alt-R CRISPR-Cas9 System from IDT (Integrated DNA Technology) where Ribonucleoprotein (RNP) complexes were assembled in vitro. The transfection was made with 5 × 10^5^ cells, 1 µl of each RNP complex, and 2 µl of Alt-R® Cas9 Electroporation Enhancer (IDT, 075915) using the Neon™ Transfection System (Thermo Fisher Scientific; 10 µl tips; pulse voltage 1450 V, pulse width 10, pulse number 3) and cultured in 1.2 mL into the K562 and THP-1 cells. For the deletion of the Epromoter *Cxcl1* in NIH3T3 cells, we used CRISPRIMAX lipofectamine (Invitrogen, CMAX00001) following a protocol from IDT (Alt-R CRISPR-Cas9 System: Cationic lipid delivery of CRISPR ribonucleoprotein complexes into mammalian cells). Three days after transfection, cells were cultured in 5 × 96-well plates at limited dilution (0.3 cells/100 μL/well). After 2–4 weeks, the clones were screened for homologous deletion using the kit Phire Tissue Direct PCR Master Mix (Thermo Fisher Scientific, F170L). Clones with homologous deletion were those showing a mutant band of the expected size and no wild-type band. The clones were then validated by Sanger sequencing. Primers and gRNAs are listed in Table EV3B,C.

### CRISPR/Cas9 genome insertion

For the insertion of the *NUCB1* Epromoter in K562, we used the web tool CRISPR-Cas9 guide RNA from IDT to design one guide RNA. Four primers were designed flanking and inside the inserted region allowing us to identify the wild-type and the mutant alleles. We created the knock-in insert by combining two homology arms flanking the *NUCB1* Epromoter and then used the Guide-it Long ssDNA Production System v2 (Takara, 632666) to produce a long single-stranded DNA. Transfection was performed using the Alt-R CRISPR-Cas9 System (IDT). The transfection was made with 5 × 10^5^ cells, 1 µL of RNP complex, 1 µg of ssDNA insert, 2 µl of Alt-R® Cas9 Electroporation Enhancer (IDT, 075915), and 5 µl of Alt-R™ HDR Enhancer V2 (IDT, 10007910) using the Neon™ Transfection System (Thermo Fisher Scientific; 10 µl tips; pulse voltage 1450 V, pulse width 10, pulse number 3) and cultured in 1.2 mL into the Δ3*NUCB1* K562. Three days after transfection, cells were cultured in 5 × 96-well plates at limited dilution (0.3 cells/100 μL/well). After 2–4 weeks, the clones were screened for homologous deletion using the kit Phire Tissue Direct PCR Master Mix (Thermo Fisher Scientific, F170L). Clones with homologous insertion were those showing a mutant band of the expected size and no wild-type band. Clones with heterozygous insertion were those showing a mutant band and a wild-type band of the expected size. The clones were then validated by targeted Long-read sequencing. Primers and gRNAs are listed in Table EV3B,C.

### CRISPRi targeting of the *OASL* promoter

CRISPRi-competent THP1 cells were generated by transducing cells with a dCas9–KRAB–MeCP2 lentiviral vector (Addgene #122205). Lentiviruses were produced in HEK293T cells co-transfected with the VSVG packaging plasmid via calcium phosphate precipitation. THP1 cells (0.3 × 10⁶ cells/mL) were infected twice in the presence of blasticidin selection. Single cells were seeded into 96-well plates and subsequently infected with a Crop-seq-derived lentivirus (Addgene #86708) encoding an sgRNA targeting the human CD81 promoter (Table EV3B), followed by 7 days of puromycin selection. The pool and individual clones were assessed for CD81 surface expression by cytometry. A clone exhibiting efficient CD81 knockdown was chosen for further CRISPRi experiments. Two sgRNAs against the *OASL* promoter were designed using the CRISPOR tool41, synthesized, and cloned into the Crop-seq-guide-puro vector (Genecust, Luxembourg) via the BsmBI site. A no-target sgRNA was also cloned as a control. Lentiviral vectors were amplified in Endura E. coli cells (Lucigen, 60241-2), and lentiviral particles were produced as described above. For each sgRNA, 2 × 10⁴ THP1 cells expressing dCas9–KRAB–MeCP2 were independently transduced with 2 mL of complete RPMI medium supplemented with 10% FBS and 1× Penicillin–Streptomycin–Glutamine (Thermo Fisher, 10378016). Ten days post-infection, cells were stimulated by replacing media with 2 mL of fresh media containing 100 ng/mL of IFNγ for 8 and 24 h, upon which the extraction of RNA was performed. The list of sgRNAs is provided in Table EV3B.

### Gene expression analysis

RNA was extracted using the RNeasy Plus Mini Kit (Qiagen, 74106) according to the manufacturer’s instructions with DNase treatment. 1 to 2.5 µg of total RNA was reverse transcribed using the SuperScript™ VILO™ (Thermo Fisher Scientific, 1755250). qPCR reactions were made using the SYBR green Master Mix (Thermo Fisher Scientific, 4367660) and the measurement was made using the Applied Biosystems QuantStudio 6 Flex Real-Time PCR System. 25 ng of cDNA was used for the qPCR. The relative expression was analyzed using the relative standard curve method. The *GAPDH* gene was used for normalizing samples in humans and the *Tbp* gene was used for normalizing samples in mice. Each point on the figures corresponds to an independent RNA and cDNA preparation. The mean values were calculated between the relative expression values of the biological replicates. The values were then normalized either by the unstimulated WT or by the unstimulated cells depending on the experiment. To assess genomic DNA degradation, genomic primers targeting the *PAX5* promoter sequence were used. Primers used are listed in Table EV3D.

### 3D chromatin organization of the *CD274* locus

Hi-C data in THP-1 + PMA cells were retrieved from Phanstiel et al (2017). TADs called from Hi-C experiments (HindIII) THP-1 cells were taken from Lin et al (2022). DNA interactions centered on the *CD274* promoter with the associated CHiCAGO score were taken from published Promoter Capture Hi-C (Javierre et al, 2016). All 3D chromatin interaction data were visualized using the New WashU Epigenome Browser (epigenomegateway.wustl.edu) (Li et al, 2022).

### TF disruption analysis

RNA-seq data before and after serum stimulation (0.3% FCS and 15% FCS, respectively) and with SRF inhibition were obtained from Esnault et al (2014). SRF inhibition was achieved using the specific pathway inhibitors Latrunculin B (LatB) and U0126. From the serum-induced genes, the pipeline identified the Epromoters-regulated clusters from which the genes were classified depending on their promoters (Epromoter or co-induced). For each gene, the induction fold change was calculated by comparing read counts from serum-stimulated (15% FCS) and serum-starved (0.3% FCS) conditions, both with and without SRF inhibition.

### Statistical analysis

Statistical details of experiments, including the statistical tests used and the exact value of samples can be found in the figures and figure legends. All statistical tests were performed using RStudio (2022.02.0 + 443), under version R (4.1.3). No blinding procedures were implemented in this study.

## Supplementary information

Table EV1

Table EV2

Table EV3

Peer Review File

Dataset EV1

Source data Fig. 1

Source data Fig. 2

Source data Fig. 3

Source data Fig. 4

Source data Fig. 5

Source data Fig. 6

Expanded View Figures

## Data Availability

This study includes no newly generated data deposited in external repositories. All publicly available datasets are listed in Table EV1A. Other resources are listed in Table EV1B. Processed data for all datasets is provided in Zenodo (10.5281/zenodo.14211588). Custom scripts used in this study are available at GitHub (https://github.com/Spicuglia-Lab/Epromoter-like-cluster-pipeline). A web-based tool for the identification of Epromoter-regulated clusters is available here: https://epromoters.tagc.univ-amu.fr. The source data of this paper are collected in the following database record: biostudies:S-SCDT-10_1038-S44318-025-00670-3.
