# Supplementary material for: Epromoters bind key stress-related transcription factors to regulate clusters of stress response genes
Source: EMBO J. 2026 Jan 3;45(3):901–29. doi: 10.1038/s44318-025-00670-3 (PMC12864986; doi:10.1038/s44318-025-00670-3)
Supplement: Supplementary file 1 — Table EV1 [file 44318_2025_670_MOESM1_ESM.docx]

Table EV1A: Dataset resources

| Dataset | Stress or stimulatory conditions | cell line or tissue | Transcription factor(s) | Accession number | References |
| --- | --- | --- | --- | --- | --- |
| Biddie | Dexamethasone,  Dexamethasone  +Tetracycline | murine mammary epithelial cell line | cJun and GR_AFos | GSE29983 | (Biddie *et al*, 2011) |
| Brown | TNFa | HUVEC | p65 | GSE54000 | (Brown *et al*, 2014) |
| Camps | Hypoxia | MCF-7 | HIF1a and HIF2a | GSE47533; GSE28352 | (Camps *et al*, 2014) |
| Cardamone | FCCP (uncoupling_agent) | 3T3-L1 | GPS2 | GSE80994 | (Cardamone *et al*, 2018) |
| Ebisuya | FGF-stimulated | NIH3T3 | SRF | GSE11576, GSE4739 | (Ebisuya *et al*, 2008) |
| Esnault | Serum Response | NIH3T3 | SRF | GSE45888 | (Esnault *et al*, 2014) |
| Ferrari | Serum Starvation | T47D | BDP1 | GSE120162 | (Ferrari *et al*, 2020) |
| Franco | E2+TNFa in | MCF-7 | FoxA1, ER and p65 | GSE59530 | (Franco *et al*, 2015) |
| Gualdrini | Serum Starvation + TPA | MEF | SRF | GSE75667 | (Gualdrini *et al*, 2016) |
| Hancock | Serum Starvation | HepG2 | Irb | GSE107336 | (Hancock *et al*, 2019) |
| Hogan | Il1b, TNFa | HAEC | p65 | GSE89970 | (Hogan *et al*, 2017) |
| Jin | TNFa | IMR90 | p65 | GSE43070 | (Jin *et al*, 2013) |
| Jubb | Dexamethasone | hMDM and mBMDM | GR | GSE61881 | (Jubb *et al*, 2016) |
| Jurida | IL-1a | KB | P65 | GSE64224 | (Jurida *et al*, 2015) |
| Kusnadi | TNFa | Monocytes | SREBP | GSE129229 | (Kusnadi *et al*, 2019) |
| Langlais | IFNg | BMDM | IRF1, IRF8 and PU1 | GSE77886 | (Langlais *et al*, 2016) |
| Lo | TNFa | NIH3T3 | p65 | GSE35724 | (Lo *et al*, 2013) |
| Lyu | Heat-Shock | hESCs | HSF1 | GSE105028 | (Lyu *et al*, 2018) |
| Mahat | Heat-Shock | MEF | HSF1.CS and HSF1.MM | GSE71708 | (Mahat *et al*, 2016) |
| Mancino | LPS | BMDM | IRF1, IRF8, PU1, STAT1 and STAT2 | GSE56123 | (Mancino *et al*, 2015) |
| Park | IFNg+TNFa, TNFa | Monocytes | IRF1 and p65 | GSE100383 | (Park *et al*, 2017) |
| Phanstiel | PMA (Macrophage differentiation) | THP-1 | MAF1 | GSE96800 | (Phanstiel *et al*, 2017) |
| Piccolo | IFNg | BMDM | CebpB, IRF1, JunB and STAT1 | GSE84520 | (Piccolo *et al*, 2017) |
| Porter | DSB | MCF-7 | P53 | GSE101738; GSE101737 | (Porter *et al*, 2017) |
| Purbey | IFNg | MC-38 | IRF1 and STAT1 | GSE246923 | (Purbey *et al*, 2024) |
| RamosPittol | TNFa | NIH3T3 p65 KO | p65 | GSE97468 | (Ramos Pittol *et al*, 2018) |
| Santiago | IFNa | K562 | IRF1, IRF9, STAT1 and STAT2 | GSE159462 | (Santiago-Algarra *et al*, 2021) |
| Schmidt | TNFa | SGBS adipocyte D10 | p65 | GSE60462; GSE64233 | (Schmidt *et al*, 2015) |
| Vierbuchen | Serum Response | MEF | Fos, Fosl2 and JunD | GSE83295 | (Vierbuchen *et al*, 2017) |
| Vihervaara | Heat-Shock | K562 | HSF1 and HSF2 | GSE43579; GSE89230 | (Vihervaara *et al*, 2017) |
| GSE158529 | Hypoxia (HIF-3a overexpression) | HeLa | HIF-3a | GSE158529; GSE158530 | unpublished |
| GSE278100 | Gemcitabine | KPC mouse models of PDAC | FOXA1 | GSE278100 | unpublished |

Table EV1B: other resources

| Publication | Sample | Link | Accession number | References |
| --- | --- | --- | --- | --- |
| Roadmap epigenomics, 2010 | RNA-seq in hESC | https://www-ncbi-nlm-nih-gov.proxy.insermbiblio.inist.fr/geo/query/acc.cgi?acc=GSE17312 | GSE17312 | (Bernstein *et al*, 2010) |
| Rao et al., 2014 | K562 domains list (TADs) | https://www.ncbi.nlm.nih.gov/geo/query/acc.cgi?acc=GSE63525 | GSM1551620 | (Rao *et al*, 2014) |
| Javierre et al., 2016 | Promoter Capture Hi-C interactions | https://www.cell.com/cms/10.1016/j.cell.2016.09.037/attachment/5bc79f6f-1b69-4192-8cb8-4247cc2e0f39/mmc4.zip | Data S1 | (Javierre *et al*, 2016) |
| Phanstiel et al., 2017 | Hi-C on THP-1+PMA | <https://www.encodeproject.org/experiments/ENCSR669GJD/> | ENCSR669GJD | (Phanstiel *et al.*, 2017) |
| Lin et al., 2018 | Hi-C TADS on THP-1 | https://www.ncbi.nlm.nih.gov/geo/query/acc.cgi?acc=GSE89663 | GSM2386380 | (Lin *et al*, 2022) |
| Kang et al ., 2017 | RNA-seq from human macrophages stimulated with IFN-γ | https://www-ncbi-nlm-nih-gov.proxy.insermbiblio.inist.fr/geo/query/acc.cgi?acc=GSE98368 | GSM2592822 | (Kang *et al*, 2017) |
| Madsen et al. 2020 | RNA-seq from human menchymal cells differentiated into adipocytes or osteoblasts after 14 days | https://www.ncbi.nlm.nih.gov/geo/download/?acc=GSE113253&format=file&file=GSE113253%5FGeneExpr%5FAT%2Etxt%2Egz | GSE113253 | (Madsen *et al*, 2020) |

**Supplementary references**

Bernstein BE, Stamatoyannopoulos JA, Costello JF, Ren B, Milosavljevic A, Meissner A, Kellis M, Marra MA, Beaudet AL, Ecker JR *et al* (2010) The NIH Roadmap Epigenomics Mapping Consortium. *Nat Biotechnol* 28: 1045-1048

Biddie SC, John S, Sabo PJ, Thurman RE, Johnson TA, Schiltz RL, Miranda TB, Sung M-H, Trump S, Lightman SL *et al* (2011) Transcription Factor AP1 Potentiates Chromatin Accessibility and Glucocorticoid Receptor Binding. *Molecular Cell* 43: 145-155

Brown JD, Lin CY, Duan Q, Griffin G, Federation A, Paranal RM, Bair S, Newton G, Lichtman A, Kung A *et al* (2014) NF-κB directs dynamic super enhancer formation in inflammation and atherogenesis. *Mol Cell* 56: 219-231

Camps C, Saini HK, Mole DR, Choudhry H, Reczko M, Guerra-Assunção JA, Tian YM, Buffa FM, Harris AL, Hatzigeorgiou AG *et al* (2014) Integrated analysis of microRNA and mRNA expression and association with HIF binding reveals the complexity of microRNA expression regulation under hypoxia. *Mol Cancer* 13: 28

Cardamone MD, Tanasa B, Cederquist CT, Huang J, Mahdaviani K, Li W, Rosenfeld MG, Liesa M, Perissi V (2018) Mitochondrial Retrograde Signaling in Mammals Is Mediated by the Transcriptional Cofactor GPS2 via Direct Mitochondria-to-Nucleus Translocation. *Mol Cell* 69: 757-772.e757

Ebisuya M, Yamamoto T, Nakajima M, Nishida E (2008) Ripples from neighbouring transcription. *Nat Cell Biol* 10: 1106-1113

Esnault C, Stewart A, Gualdrini F, East P, Horswell S, Matthews N, Treisman R (2014) Rho-actin signaling to the MRTF coactivators dominates the immediate transcriptional response to serum in fibroblasts. *Genes Dev* 28: 943-958

Ferrari R, de Llobet Cucalon LI, Di Vona C, Le Dilly F, Vidal E, Lioutas A, Oliete JQ, Jochem L, Cutts E, Dieci G *et al* (2020) TFIIIC Binding to Alu Elements Controls Gene Expression via Chromatin Looping and Histone Acetylation. *Mol Cell* 77: 475-487.e411

Franco HL, Nagari A, Kraus WL (2015) TNFalpha signaling exposes latent estrogen receptor binding sites to alter the breast cancer cell transcriptome. *Mol Cell* 58: 21-34

Gualdrini F, Esnault C, Horswell S, Stewart A, Matthews N, Treisman R (2016) SRF Co-factors Control the Balance between Cell Proliferation and Contractility. *Mol Cell* 64: 1048-1061

Hancock ML, Meyer RC, Mistry M, Khetani RS, Wagschal A, Shin T, Ho Sui SJ, Näär AM, Flanagan JG (2019) Insulin Receptor Associates with Promoters Genome-wide and Regulates Gene Expression. *Cell* 177: 722-736.e722

Hogan NT, Whalen MB, Stolze LK, Hadeli NK, Lam MT, Springstead JR, Glass CK, Romanoski CE (2017) Transcriptional networks specifying homeostatic and inflammatory programs of gene expression in human aortic endothelial cells. *Elife* 6

Javierre BM, Burren OS, Wilder SP, Kreuzhuber R, Hill SM, Sewitz S, Cairns J, Wingett SW, Varnai C, Thiecke MJ *et al* (2016) Lineage-Specific Genome Architecture Links Enhancers and Non-coding Disease Variants to Target Gene Promoters. *Cell* 167: 1369-1384 e1319

Jin F, Li Y, Dixon JR, Selvaraj S, Ye Z, Lee AY, Yen CA, Schmitt AD, Espinoza CA, Ren B (2013) A high-resolution map of the three-dimensional chromatin interactome in human cells. *Nature* 503: 290-294

Jubb AW, Young RS, Hume DA, Bickmore WA (2016) Enhancer Turnover Is Associated with a Divergent Transcriptional Response to Glucocorticoid in Mouse and Human Macrophages. *J Immunol* 196: 813-822

Jurida L, Soelch J, Bartkuhn M, Handschick K, Muller H, Newel D, Weber A, Dittrich-Breiholz O, Schneider H, Bhuju S *et al* (2015) The Activation of IL-1-Induced Enhancers Depends on TAK1 Kinase Activity and NF-kappaB p65. *Cell Rep* 10: 726-739

Kang K, Park SH, Chen J, Qiao Y, Giannopoulou E, Berg K, Hanidu A, Li J, Nabozny G, Park-Min KH *et al* (2017) Interferon-γ Represses M2 Gene Expression in Human Macrophages by Disassembling Enhancers Bound by the Transcription Factor MAF. *Immunity* 47: 235-250.e234

Kusnadi A, Park SH, Yuan R, Pannellini T, Giannopoulou E, Oliver D, Lu T, Park-Min KH, Ivashkiv LB (2019) The Cytokine TNF Promotes Transcription Factor SREBP Activity and Binding to Inflammatory Genes to Activate Macrophages and Limit Tissue Repair. *Immunity* 51: 241-257.e249

Langlais D, Barreiro LB, Gros P (2016) The macrophage IRF8/IRF1 regulome is required for protection against infections and is associated with chronic inflammation. *J Exp Med* 213: 585-603

Lin D, Xu W, Hong P, Wu C, Zhang Z, Zhang S, Xing L, Yang B, Zhou W, Xiao Q *et al* (2022) Decoding the spatial chromatin organization and dynamic epigenetic landscapes of macrophage cells during differentiation and immune activation. *Nature communications* 13: 5857

Lo KA, Labadorf A, Kennedy NJ, Han MS, Yap YS, Matthews B, Xin X, Sun L, Davis RJ, Lodish HF *et al* (2013) Analysis of in vitro insulin-resistance models and their physiological relevance to in vivo diet-induced adipose insulin resistance. *Cell Rep* 5: 259-270

Lyu X, Rowley MJ, Corces VG (2018) Architectural Proteins and Pluripotency Factors Cooperate to Orchestrate the Transcriptional Response of hESCs to Temperature Stress. *Molecular Cell* 71: 940-955.e947

Madsen JGS, Madsen MS, Rauch A, Traynor S, Van Hauwaert EL, Haakonsson AK, Javierre BM, Hyldahl M, Fraser P, Mandrup S (2020) Highly interconnected enhancer communities control lineage-determining genes in human mesenchymal stem cells. *Nat Genet* 52: 1227-1238

Mahat DB, Kwak H, Booth GT, Jonkers IH, Danko CG, Patel RK, Waters CT, Munson K, Core LJ, Lis JT (2016) Base-pair-resolution genome-wide mapping of active RNA polymerases using precision nuclear run-on (PRO-seq). *Nature protocols* 11: 1455-1476

Mancino A, Termanini A, Barozzi I, Ghisletti S, Ostuni R, Prosperini E, Ozato K, Natoli G (2015) A dual cis-regulatory code links IRF8 to constitutive and inducible gene expression in macrophages. *Genes Dev* 29: 394-408

Park SH, Kang K, Giannopoulou E, Qiao Y, Kim G, Park-Min KH, Ivashkiv LB (2017) Type I interferons and the cytokine TNF cooperatively reprogram the macrophage epigenome to promote inflammatory activation. *Nat Immunol* 18: 1104-1116

Phanstiel DH, Van Bortle K, Spacek D, Hess GT, Shamim MS, Machol I, Love MI, Aiden EL, Bassik MC, Snyder MP (2017) Static and Dynamic DNA Loops form AP-1-Bound Activation Hubs during Macrophage Development. *Mol Cell* 67: 1037-1048.e1036

Piccolo V, Curina A, Genua M, Ghisletti S, Simonatto M, Sabo A, Amati B, Ostuni R, Natoli G (2017) Opposing macrophage polarization programs show extensive epigenomic and transcriptional cross-talk. *Nat Immunol* 18: 530-540

Porter JR, Fisher BE, Baranello L, Liu JC, Kambach DM, Nie Z, Koh WS, Luo J, Stommel JM, Levens D *et al* (2017) Global Inhibition with Specific Activation: How p53 and MYC Redistribute the Transcriptome in the DNA Double-Strand Break Response. *Mol Cell* 67: 1013-1025.e1019

Purbey PK, Seo J, Paul MK, Iwamoto KS, Daly AE, Feng AC, Champhekar AS, Langerman J, Campbell KM, Schaue D *et al* (2024) Opposing tumor-cell-intrinsic and -extrinsic roles of the IRF1 transcription factor in antitumor immunity. *Cell Rep* 43: 114289

Ramos Pittol JM, Oruba A, Mittler G, Saccani S, van Essen D (2018) Zbtb7a is a transducer for the control of promoter accessibility by NF-kappa B and multiple other transcription factors. *PLOS Biology* 16: e2004526

Rao SSP, Huntley MH, Durand NC, Stamenova EK, Bochkov ID, Robinson JT, Sanborn A, Machol I, Omer AD, Lander ES *et al* (2014) A three-dimensional map of the human genome at kilobase resolution reveals principles of chromatin looping. *Cell* 159: 1665-1680

Santiago-Algarra D, Souaid C, Singh H, Dao LTM, Hussain S, Medina-Rivera A, Ramirez-Navarro L, Castro-Mondragon JA, Sadouni N, Charbonnier G *et al* (2021) Epromoters function as a hub to recruit key transcription factors required for the inflammatory response. *Nature communications* 12: 6660

Schmidt SF, Larsen BD, Loft A, Nielsen R, Madsen JG, Mandrup S (2015) Acute TNF-induced repression of cell identity genes is mediated by NFkappaB-directed redistribution of cofactors from super-enhancers. *Genome Res* 25: 1281-1294

Vierbuchen T, Ling E, Cowley CJ, Couch CH, Wang X, Harmin DA, Roberts CWM, Greenberg ME (2017) AP-1 Transcription Factors and the BAF Complex Mediate Signal-Dependent Enhancer Selection. *Mol Cell* 68: 1067-1082.e1012

Vihervaara A, Mahat DB, Guertin MJ, Chu T, Danko CG, Lis JT, Sistonen L (2017) Transcriptional response to stress is pre-wired by promoter and enhancer architecture. *Nature communications* 8: 255
