# Supplementary material for: Epromoters bind key stress-related transcription factors to regulate clusters of stress response genes
Source: EMBO J. 2026 Jan 3;45(3):901–29. doi: 10.1038/s44318-025-00670-3 (PMC12864986; doi:10.1038/s44318-025-00670-3)
Supplement: Supplementary file 2 — Table EV2 [file 44318_2025_670_MOESM2_ESM.docx]

Table EV2: Summary of stress-induced gene clusters

| Datasets | Organism | Cell types | Stimulation | Transcription factors | Conditions | Total clusters | Epromoter-regulated clusters | Epromoter-regulated clusters Frequency (%) |
| --- | --- | --- | --- | --- | --- | --- | --- | --- |
| Biddie | mm10 | Murine mammary epithelial cell line, 3134 | Dexamethasone | cJun | Hormone | 5 | 1 | 20.0 |
| Biiddie | mm10 | Murine mammary epithelial cell line, 3135 | Dexamethasone  +Tetracycline | GR_AFos | Hormone | 11 | 1 | 9.1 |
| Brown | hg19 | HUVEC | TNFa | p65 | Inflammatory response | 15 | 5 | 33.3 |
| Camps | hg18 | MCF-7 | Hypoxia | HIF1a | Respiratory stress | 11 | 0 | 0.0 |
| Camps | hg18 | MCF-7 | Hypoxia | HIF2a | Respiratory stress | 11 | 0 | 0.0 |
| Cardamone | mm10 | 3T3-L1 | FCCP (uncoupling_agent) | GPS2 | Respiratory stress | 26 | 0 | 0.0 |
| Ebisuya | mm9 | NIH3T3 | FGF-stimulated | SRF | FGF | 17 | 3 | 17.6 |
| Esnault | mm9 | NIH3T3 | Serum Response | SRF | Serum | 56 | 20 | 35.7 |
| Ferrari | hg38 | T47D | Serum Starvation | BDP1 | Serum | 109 | 0 | 0.0 |
| Franco | hg38 | MCF-7 | E2+TNFa | FoxA1 | Hormone | 58 | 5 | 8.6 |
| Franco | hg38 | MCF-7 | E2+TNFa | ER | Hormone | 58 | 22 | 37.9 |
| Franco | hg38 | MCF-7 | E2+TNFa | p65 | Hormone | 58 | 3 | 5.2 |
| Gualdrini | mm9 | MEF | Serum Starvation + TPA | SRF | Serum | 170 | 11 | 6.5 |
| Hancock | hg19 | HepG2 | Insulin | Irb | Hormone | 194 | 74 | 38.1 |
| Hogan | hg38 | HAEC | Il1b | p65 | Inflammatory response | 53 | 24 | 45.3 |
| Hogan | hg38 | HAEC | TNFa | p65 | Inflammatory response | 38 | 19 | 50.0 |
| Jin | hg38 | IMR90 | TNFa | p65 | Inflammatory response | 13 | 2 | 15.4 |
| Jubb | mm9 | hMDM | Dexamethasone | GR.hMDM | Hormone | 8 | 0 | 0.0 |
| Jubb | hg19 | mBMDM | Dexamethasone | GR.mBMDM | Hormone | 8 | 0 | 0.0 |
| Jurida | hg19 | KB | Il-1a | p65 | Inflammatory response | 13 | 1 | 7.7 |
| Kusnadi | hg19 | Monocytes | TNFa | SREBP | Inflammatory response | 117 | 50 | 42.7 |
| Langlais | mm9 | BMDM | IFNg | IRF1 | Inflammatory response | 67 | 29 | 43.3 |
| Langlais | mm9 | BMDM | IFNg | IRF8 | Inflammatory response | 67 | 22 | 32.8 |
| Langlais | mm9 | BMDM | IFNg | PU1 | Inflammatory response | 67 | 11 | 16.4 |
| Lo | mm9 | NIH3T3 | TNFa | p65 | Inflammatory response | 143 | 9 | 6.3 |
| Lyu | hg38 | hESCs | Heat_Shock | HSF1 | Heat-Shock | 78 | 12 | 15.4 |
| Mahat | mm10 | MEF | Heat_Shock | HSF1.CS | Heat-Shock | 63 | 6 | 9.5 |
| Mahat | mm10 | MEF | Heat_Shock | HSF1.MM | Heat-Shock | 63 | 11 | 17.5 |
| Mancino | mm10 | BMDM | LPS | IRF1 | Inflammatory response | 19 | 8 | 42.1 |
| Mancino | mm9 | BMDM | LPS | IRF8 | Inflammatory response | 19 | 10 | 52.6 |
| Mancino | mm9 | BMDM | LPS | PU1 | Inflammatory response | 19 | 8 | 42.1 |
| Mancino | mm9 | BMDM | LPS | STAT1 | Inflammatory response | 19 | 7 | 36.8 |
| Mancino | mm9 | BMDM | LPS | STAT2 | Inflammatory response | 19 | 7 | 36.8 |
| Park | hg38 | Monocytes | IFNg+TNFa | IRF1 | Inflammatory response | 28 | 2 | 7.1 |
| Park | hg38 | Monocytes | TNFa | p65 | Inflammatory response | 93 | 14 | 15.1 |
| Phanstiel | hg19 | THP-1 | PMA  (Macrophage differentiation) | MAF1 | Macrophage differentiation | 735 | 247 | 33.6 |
| Piccolo | mm10 | BMDM | IFNg | CebpB | Inflammatory response | 7 | 5 | 71.4 |
| Piccolo | mm10 | BMDM | IFNg | IRF1 | Inflammatory response | 7 | 4 | 57.1 |
| Piccolo | mm10 | BMDM | IFNg | JunB | Inflammatory response | 7 | 4 | 57.1 |
| Piccolo | mm10 | BMDM | IFNg | STAT1 | Inflammatory response | 7 | 4 | 57.1 |
| Porter | hg19 | MCF-7 | DSB | p53 | DSB | 18 | 0 | 0.0 |
| Purbey | mm9 | MC-38 | IFNg | IRF1 | Inflammatory response | 15 | 8 | 53.3 |
| Purbey | mm9 | MC-38 | IFNg | STAT1 | Inflammatory response | 15 | 6 | 40 |
| RamosPittol | mm9 | NIH3T3 | KO p65 + TNFa | p65 | Inflammatory response | 11 | 4 | 36.4 |
| Santiago | hg19 | K562 | IFNa | IRF1 | Inflammatory response | 37 | 17 | 45.9 |
| Santiago | hg19 | K562 | IFNa | IRF9 | Inflammatory response | 37 | 15 | 40.5 |
| Santiago | hg19 | K562 | IFNa | STAT1 | Inflammatory response | 37 | 14 | 37.8 |
| Santiago | hg19 | K562 | IFNa | STAT2 | Inflammatory response | 37 | 15 | 40.5 |
| Schmidt | hg19 | SGBS adipocyte D10 | TNFa | p65 | Inflammatory response | 424 | 124 | 29.2 |
| Vierbuchen | mm10 | MEF | Serum Response | Fos | Serum | 97 | 46 | 47.4 |
| Vierbuchen | mm10 | MEF | Serum Response | Fosl2 | Serum | 97 | 16 | 16.5 |
| Vierbuchen | mm10 | MEF | Serum Response | JunD | Serum | 97 | 32 | 33.0 |
| Vihervaara | hg19 | K562 | Heat_Shock | HSF1 | Heat-Shock | 75 | 17 | 22.7 |
| Vihervaara | hg19 | K562 | Heat_Shock | HSF2 | Heat-Shock | 75 | 13 | 17.3 |
| GSE158529 | hg38 | HeLA | Hypoxia  (HIF-3a overexpression) | HIF-3a | Respiratory stress | 20 | 4 | 20 |
| GSE278100 | mm10 | KPC mouse models of PDAC | Gemcitabine | FOXA1 | Genotoxic drug | 14 | 5 | 35.7 |
