# Supplementary material for: Epromoters bind key stress-related transcription factors to regulate clusters of stress response genes
Source: EMBO J. 2026 Jan 3;45(3):901–29. doi: 10.1038/s44318-025-00670-3 (PMC12864986; doi:10.1038/s44318-025-00670-3)
Supplement: Supplementary file 3 — Table EV3 [file 44318_2025_670_MOESM3_ESM.docx]

Table EV3A: List of Luciferase assays cloned regions

| Name | Genome | Coordinates | Size | Cloning site |
| --- | --- | --- | --- | --- |
| pGL4_DHDH | hg19 | chr19:49,436,706-49,437,206 | 501 | BglII/HindIII |
| pGL4_HSPA1A | hg19 | chr6:31,782,891-31,783,404 | 513 | BglII / HindIII |
| pGL4_NUCB1_DHDH | hg19 | chr19:49,403,188-49,403,688 and chr19:49,436,706-49,437,206 | 501 | BglII / HindIII and BamHI / SalI |
| pGL4_sv40 Luc2CP |  | from the pGl3-sv40 plasmid (E176, Promega) | 197 | BglII / HindIII |
| pGL4_sv40 RLCP |  | from the pGl3-sv40 plasmid (E176, Promega) | 197 | BglII / HindIII |
| pGL4_sv40_AHSA2P | hg19 | chr2:61,404,303-61,404,803 | 501 | BamHI / SalI |
| pGL4_sv40_AP4B1-DCLRE18 | hg19 | chr1:114,447,365-114,447,865 | 501 | BamHI / SalI |
| pGL4_sv40_BST2 | hg19 | chr19:17,516,186-17,516,686 | 501 | BamHI / SalI |
| pGL4_sv40_CRYAB-HSPB2 | hg19 | chr11:111,781,991-111,782,491 | 501 | BamHI / SalI |
| pGL4_sv40_DHDH | hg19 | chr19:49,436,706-49,437,206 | 501 | BamHI / SalI |
| pGL4_sv40_DUSP1 | hg19 | chr5:172,199,425-172,199,925 | 501 | BamHI / SalI |
| pGL4_sv40_EPB42 | hg19 | chr15:43,513,183 - 43,513,683 | 501 | BamHI / SalI |
| pGL4_sv40_ERGIC1 | hg19 | chr5:172,260,973-172,261,473 | 501 | BamHI / SalI |
| pGL4_sv40_HSP90AB1 | hg19 | chr6:44,213,931-44,214,431 | 501 | BamHI / SalI |
| pGL4_sv40_HSPB1 | hg19 | chr7:75,931,582-75,932,082 | 501 | BamHI / SalI |
| pGL4_sv40_HSPD1-HSPE1 | hg19 | chr2:198,364,570-198,365,070 | 501 | BamHI / SalI |
| pGL4_sv40_MBOAT7 | hg19 | chr19:54,692,006-54,692,506 | 501 | BamHI / SalI |
| pGL4_sv40_MOB4 | hg19 | chr2:198,380,045-198,380,545 | 501 | BamHI / SalI |
| pGL4_sv40_NUCB1 | hg19 | chr19:49,403,188-49,403,688 | 501 | BamHI / SalI |
| pGL4_sv40_PGGHG | hg19 | chr11:287,350-287,850 | 501 | BamHI / SalI |
| pGL4_sv40_PMVK | hg19 | chr1:154,909,200-154,909,700 | 501 | BamHI / SalI |
| pGL4_sv40_PPP1R15A | hg19 | chr19:49,375,399-49,375,899 | 501 | BamHI / SalI |
| pGL4_sv40_PTGES3 | hg19 | chr12:57,081,923-57,082,423 | 501 | BamHI / SalI |
| pGL4_sv40_RIC8A | hg19 | chr11:207,249-207,749 | 501 | BamHI / SalI |
| pGL4_sv40_SCL35B2 | hg19 | chr6:44,225,033-44,225,533 | 501 | BamHI / SalI |
| pGL4_sv40_SNAP23 | hg19 | chr15:42,787,065-42,787,565 | 501 | BamHI / SalI |
| pGL4_sv40_STAT2 | hg19 | chr12:56,753,808-56,754,308 | 501 | BamHI / SalI |
| pGL4_sv40_STIP1 | hg19 | chr11:63,953,248-63,953,748 | 501 | BamHI / SalI |
| pGL4_sv40_TCP1-MPRL18 | hg19 | chr6:160,210,646-160,211,146 | 501 | BamHI / SalI |
| pGL4_sv40_TNNI3 | hg19 | chr19:55,669,991-55,670,491 | 501 | BamHI / SalI |
| pGL4_sv40_UBC | hg19 | chr12:125,399,749-125,400,249 | 501 | BamHI / SalI |
| pGL4_sv40_YWHAG | hg19 | chr7:75,988,092-75,988,592 | 501 | BamHI / SalI |

Table EV3B: CRISPR-Cas9 guide RNA sequences

| Name | Sequence (5'-3') | Expected deleted region |
| --- | --- | --- |
| NUCB1 gRNA1 | TAAAGAGACGGATGTCAAGC | 742 bp |
| NUCB1 gRNA2 | GAAAACGCCCTCTGCGGTGA |  |
| Cxcl1_gRNA1 | ACGCCACCTAGGATTCCACT | 571 bp |
| Cxcl1_gRNA2 | ATTGGCGATAGGCGCCCCTA |  |
| PDL1 gRNA1 | TATTGAGATAGCCCTTGCATTGG | 1891 bp |
| PDL1 gRNA2 | AACCTTTATACCCAACAGTGAGG |  |
| CD81 sgRNA | GCCTGGCAGGATGCGCGGTG |  |
| CTL sgRNA | GGGAACGACTATGACCGCCA |  |
| OASL sgRNA1 | AGAGTTCTCCTTTATGACGT |  |
| OASL sgRNA2 | CTGCAGCCACTGAGCCACGA |  |
|  |  | Expected inserted region |
| NUCB1_ins_gRNA1 | CTCAGGAAACTAATAGCGTG | 584 bp |

Table EV3C: Genome editing control primers

| Name | Sequence (5'-3') | Expected WT size | Expected Mutant size |
| --- | --- | --- | --- |
| NUCB1-del-F | TTAGTGGAGGCGAGGTTTCT | 1120 bp | 378 bp |
| NUCB1-del-R | CTTCCCTTCCTCCGACACAG |  |  |
| Cxcl1-del-F | GTTGGCAAAAGCAAACCACC | 944 bp | 373 bp |
| Cxcl1-del-R | CTCGCGACCATTCTTGAGTG |  |  |
| PDL1-del-F | GGCCCAAACCCTATTGCAAT | 2341 bp | 450 bp |
| PDL1-del-R | TGAAGACCACACTCTAACTGCT |  |  |
| NUCB1-ins-ext-F | GGGTAGTGCTAACATGTGCG | 1300 bp | 1877 bp |
| NUCB1-ins-ext-R | TTCACGAGAAAGCCACCCC |  |  |
| NUCB1-ins-int-F | AAATAACTTCCGCGGGCATC | - | 712 bp (with ext primer) |
| NUCB1-ins-int-R | GATGCCCGCGGAAGTTATTT | - | 1185 bp (with ext primer) |

Table EV3D: qPCR primers

| Name | Sequence (5'-3') |
| --- | --- |
| NUCB1_qpcr_FW | GGACCCTCAGAACCAGCATA |
| NUCB1_qpcr_Rev | TCTCGTAGCGCTTGAACTCT |
| DHDH_qpcr_FW | GACTGCAATTTTGACAACGGG |
| DHDH_qpcr_Rev | GACAGGGGAATCACAGGACT |
| TULP2_qpcr_FW | TAGGTCCCCAGCTTCCAAAG |
| TULP2_qpcr_Rev | GCTTCTGTCGCTGCTTCTTT |
| KI_acti_prom_qpcr_FW | ACCCTTCCGTTGTCTGCATA |
| KI_acti_prom_qpcr_Rev | CAGGATCTTTGGGGTGTTGC |
| PPP1R15A_qpcr_FW | GCCCAGAAACCCCTACTCAT |
| PPP1R15A_qpcr_Rev | CCCAGACAGCCAGGAAATG |
| PLEKHA4_qpcr_FW | TCCCCTGGAGTCAACTTTCC |
| PLEKHA4_qpcr_Rev | CTATCTCCTCCTGCAGCCTC |
| GYS1_qpcr_FW | AGTTCCTCTCCTCCACAAGC |
| GYS1_qpcr_Rev | GGATTCCCATAACCGTGCAC |
| HSPA1A_qpcr_FW | TGCGACAGTCCACTACCTTT |
| HSPA1A_qpcr_Rev | AACACTGGATCCGCGAGAA |
| CD274_qpcr_FW | GGAAAACCATACAGCTGAATTGG |
| CD274_qpcr_Rev | TGGCTCCCAGAATTACCAA GT |
| JAK2_qpcr_FW | CCATTCCCTTGGGAAATCTG |
| JAK2_qpcr_Rev | AGGTGTGATACCACAAGCT |
| GAPDH_qpcr_FW | CCCACTCCTCCACCTTTGAC |
| GAPDH_qpcr_Rev | CCACCACCCTGTTGCTGTAG |
| Cxcl1_qpcr_FW | CTCAAGAATGGTCGCGAGG |
| Cxcl1_qpcr_Rev | CTTCTTTCTCCGTTACTTGGGG |
| Cxcl2_qpcr_FW | AGTCATAGCCACTCTCAAGGG |
| Cxcl2_qpcr_Rev | GTCAGTTAGCCTTGCCTTTGT |
| Tbp_qpcr_FW | TATCACTCCTGCCACACCAG |
| Tbp_qpcr_Rev | ACAGCCAAGATTCACGGTAGA |
| OASL qpcr FW | GAAACATCGGCCAACTAAGCT |
| OASL qpcr Rev | GTCAAGTGGATGTCTCGTGC |
| P2RX7 qpcr FW | AGGAAGAAGTGCGAGTCCAT |
| P2RX7 qpcr Rev | CCCTAGTAGCTGCTGGTTCA |
