# Supplementary material for: Epromoters bind key stress-related transcription factors to regulate clusters of stress response genes
Source: EMBO J. 2026 Jan 3;45(3):901–29. doi: 10.1038/s44318-025-00670-3 (PMC12864986; doi:10.1038/s44318-025-00670-3)
Supplement: Supplementary file 12 — Expanded View Figures [file 44318_2025_670_MOESM12_ESM.pdf]

Expanded View Figures

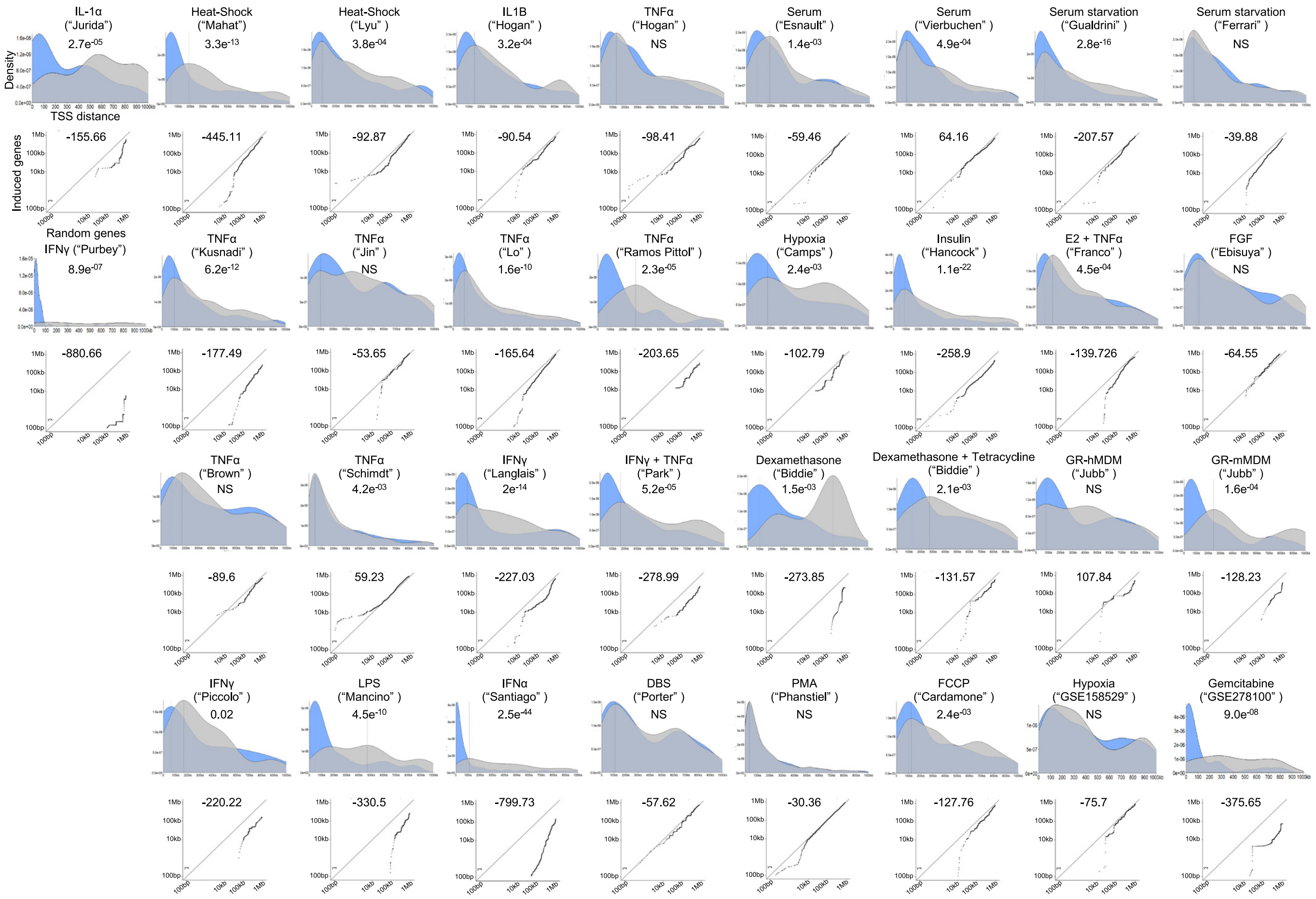

Figure EV1. Genomic distance between stress responses induced genes for all stress-related datasets, related to Fig. 1.

Q16 **(A)** The top panels display the distance distribution of the induced genes (blue) compared to the same numbers of random genes (gray) by dataset. The bottom panels display the deviation score calculated between induced gene distance and random gene distance for each dataset. The *P* values for the distance distribution were calculated by Kolmogorov–Smirnov (KS) test.

© The Author(s)

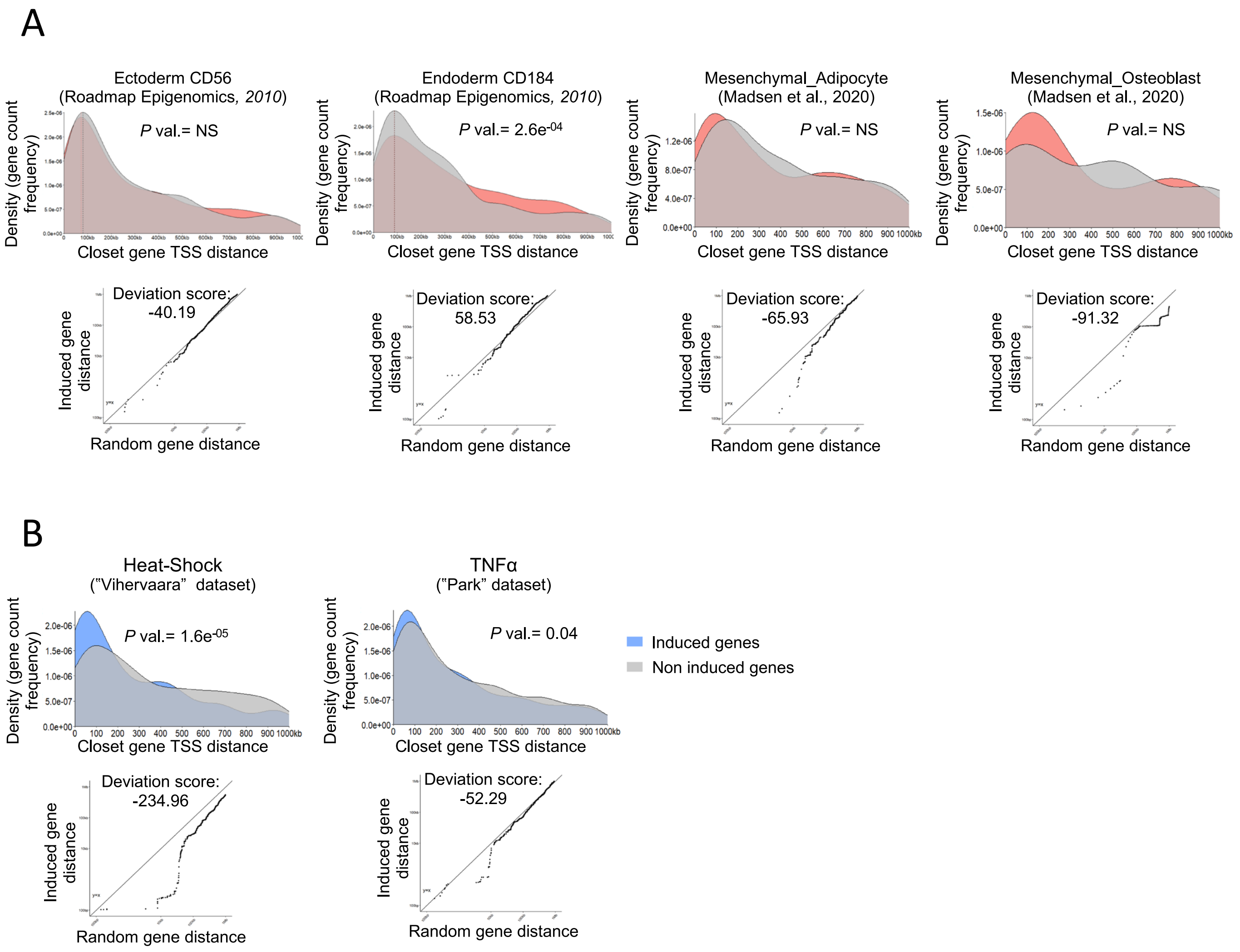

Figure EV2. Genomic distance between induced genes for control datasets, related to Fig. 1.

(**A**) The top panels display the distance distribution of the induced genes (salmon) compared to the same numbers of random genes (gray) for each dataset of differentiation. The bottom panels display the deviation score calculated between the induced and random gene distances for each dataset. *P* values were calculated by Kolmogorov–Smirnov (KS) test. (**B**) The top panels display the distance distribution of the induced genes (blue) compared to the same numbers of non-induced genes for the same dataset (gray). The bottom panels display the deviation score calculated between induced gene distance and non-induced gene distance for each dataset. *P* values were calculated by Kolmogorov–Smirnov (KS) test.

 © The Author(s)

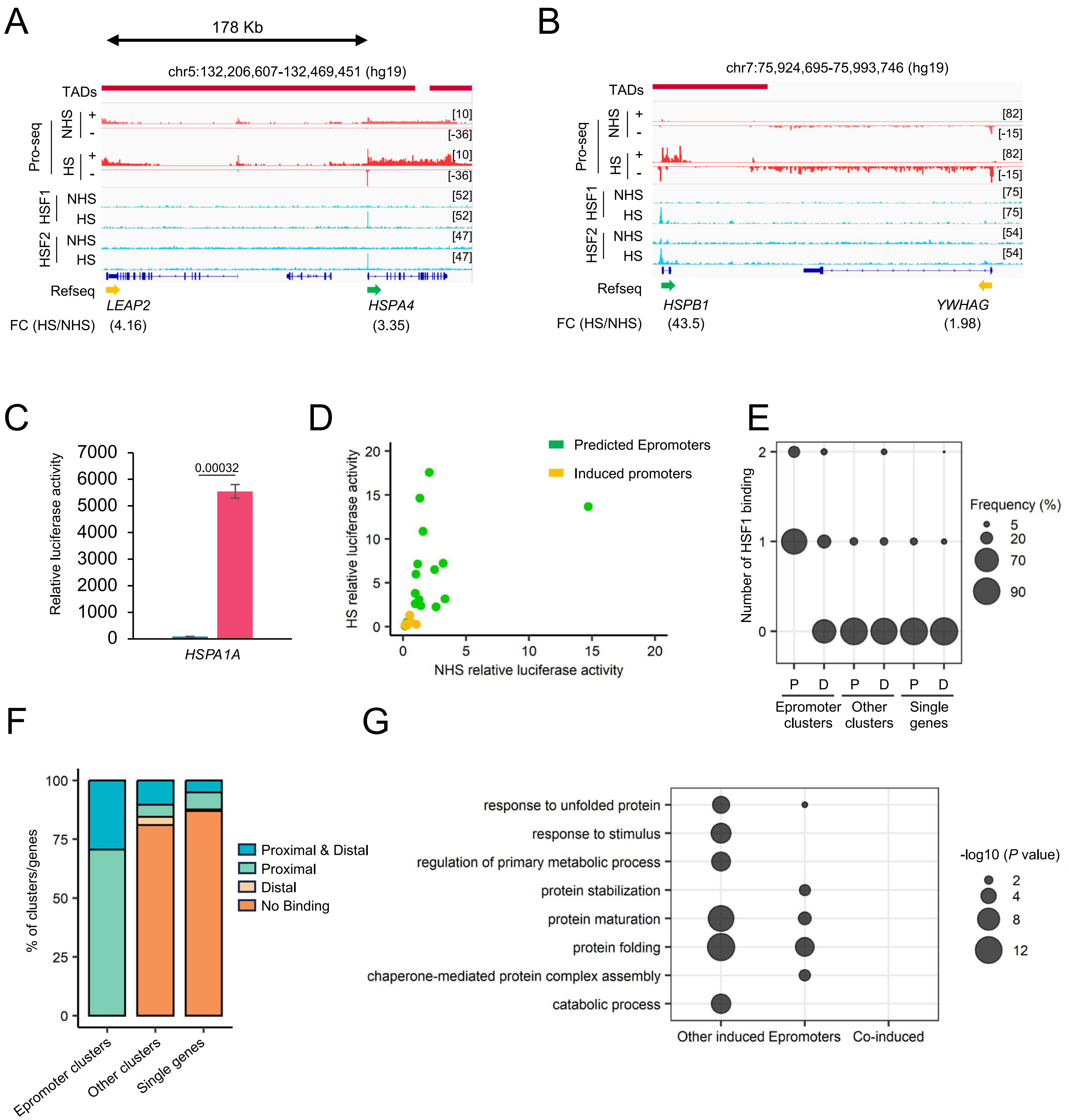
A
178 Kb
chr5:132,206,607-132,469,451 (hg19)
TADs
Pro-seq
NHS
HS
HSF1
HSF2
Refseq
LEAP2
HSPA4
FC (HS/NHS)
(4.16)
(3.35)
B
chr7:75,924,695-75,993,746 (hg19)
HSPB1
YWHAG
(43.5)
(1.98)
C
Relative luciferase activity
0.00032
HSPA1A
D
Predicted Epromoters
Induced promoters
HS relative luciferase activity
NHS relative luciferase activity
E
Number of HSF1 binding
Frequency (%)
Epromoter clusters
Other clusters
Single genes
F
% of clusters/genes
Proximal & Distal
Proximal
Distal
No Binding
Epromoter clusters
Other clusters
Single genes
G
response to unfolded protein
response to stimulus
regulation of primary metabolic process
protein stabilization
protein maturation
protein folding
chaperone-mediated protein complex assembly
catabolic process
Other induced Epromoters
Co-induced
-log10 (P value)

© The Author(s)

Figure EV3. Heat shock response model from the "Vihervaara" dataset, related to Fig. 2.

(**A**) Example of the *HSPA4* Epromoter-regulated cluster identified specifically by the pipeline using the TAD clustering method in the HS stress response. The genomic tracks show the PRO-seq signal (in red) and HSF1 and HSF2 ChIP-seq signal (in blue) before or after HS from the "Vihervaara" dataset. The topological associated domains (TAD) from K562 are at the top. The fold-change of induction is indicated below the name and orientation of the genes (Epromoter: green, co-induced genes: yellow). (**B**) Example of the *HSPB1* Epromoter-regulated cluster identified specifically by the pipeline using the distance clustering method in the HS stress response. The genomic tracks show the PRO-seq signal (in red) and HSF1 and HSF2 ChIP-seq signal (in blue) before or after HS from the "Vihervaara" dataset. The topological associated domains (TAD) from K562 are at the top. The fold-change of induction is indicated below the name and orientation of the genes (Epromoter: green, co-induced genes: yellow). (**C**) Luciferase assay to quantify the promoter activity of the control *HSPA1A* promoter before (blue) and after (red) HS in the K562 cells. The results were normalized on the Renilla activity. All data represent mean values ± SD of three biological replicates. *P* value was calculated by a paired one-sided Student's t-test. (**D**) Scatterplot of the luciferase signal to quantify the enhancer activity of predicted Epromoter before (x-axis) and after (y-axis) HS. The predicted Epromoter (green) and the induced promoters (yellow) are highlighted. (**E**) Number of distal and proximal HSF1 binding associated with HS-induced genes for the indicated categories. (**F**) Percentage of clusters or single HS-induced genes associated with HSF1 binding at either proximal, distal or both genomic regions. (**G**) Significantly enriched GO biological processes for the HS-induced genes in K562, depending on their associated promoter. *P* value was calculated by an hypergeometric test using g:Profiler.

 © The Author(s)

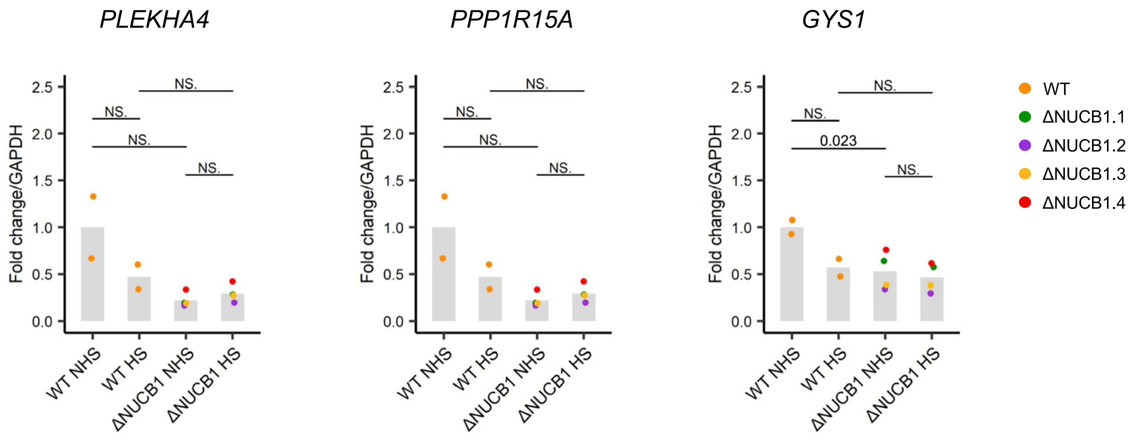

Figure EV4. Gene expression of neighbor genes after deletion of the *NUCB1* Epromoter, related to Fig. 4.

qPCR analysis of *PLEKHA4*, *PPP1R15A*, and *GYS1* expression in wild-type and 4 ΔNUCB1 mutants in K562 cells before and after HS. Values represent the relative expression of the samples normalized to the housekeeping gene *GAPDH* and compared to the unstressed wild-type cells. The value for each biological replicate is shown. *P* values were calculated by a two-sided Student's t-test.

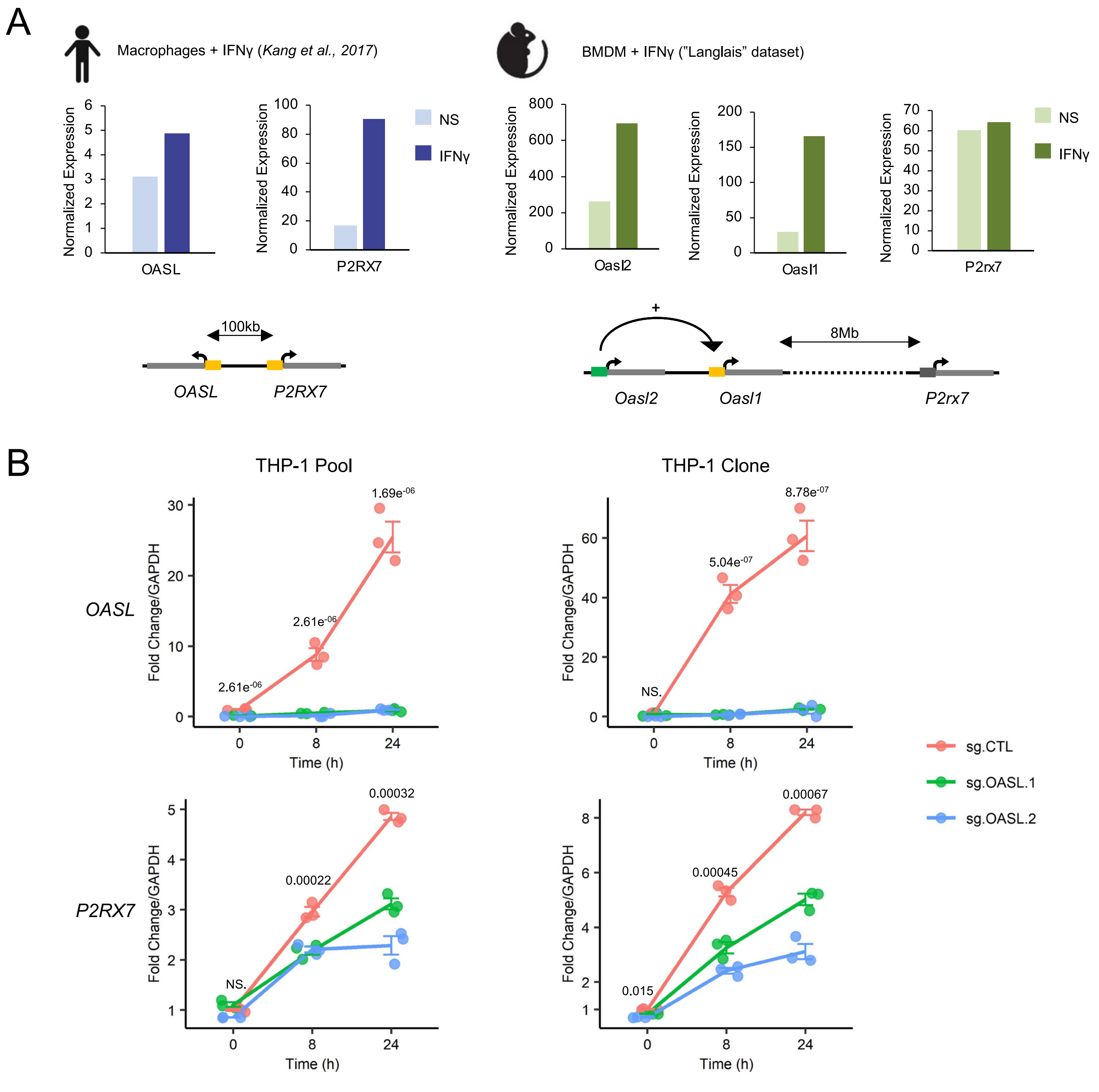

Figure EV5. Regulation of *OASL/P2RX7* in mouse and human macrophages, related to Figs. 3 and 5.

(**A**) (top panel) Mean normalized counts for *P2RX7/P2rx7* and *OASL/Oasl1/Oasl2* in unstimulated and IFNγ-stimulated human (blue) or mouse (green) macrophages. Data were retrieved from the "Langlais" dataset and from (63). (bottom panel) Schematic representation of the locus in the human (left) and mouse (right). (**B**) qPCR analysis of *OASL* and *P2RX7* expression in dCas9-KRAB-MeCP2 THP-1, as a pool or as a clonal cell line, infected with either a negative control (sg.CTL) or two different single guide RNAs against *OASL* (sg.OASL.1 and sg.OASL.2). Values represent the relative expression of the samples normalized to the housekeeping gene *GAPDH*. All data represent mean values ± SD of three technical replicates. *P*-values for group differences (sg.CTL vs. sg.OASL) at each time point were calculated by one-way ANOVA with Benjamini–Hochberg correction.

Q13

© The Author(s)

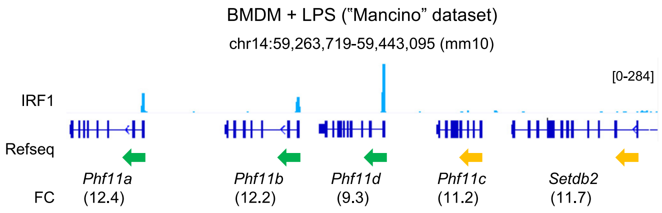

Figure EV6. Screenshot of the *Phf11* cluster by LPS stimulation from the "Mancino" dataset, related to the discussion.

The genomic tracks show the ChIP-seq signal of IRF1 after stimulation. The fold-change of induction is indicated below the name and orientation of the genes (Epromoter: green, co-induced genes: yellow). Note that all four *Phf11* and *Setdb2* genes are induced, but all except *Phf11c* and *Setdb2* bind IRF1.
