# Supplementary material for: Epromoters bind key stress-related transcription factors to regulate clusters of stress response genes
Source: EMBO J. 2026 Jan 3;45(3):901–29. doi: 10.1038/s44318-025-00670-3 (PMC12864986; doi:10.1038/s44318-025-00670-3)
Supplement: Supplementary file 4 — Peer Review File [file 44318_2025_670_MOESM4_ESM.pdf]

THE EMBO JOURNAL

Epromoters bind key stress-related transcription factors to regulate clusters of stress response genes

Juliette Malfait, Jing Wan, Himanshu Singh, Charbel Souaid, Gaëlle Farah, Junhua Su, Magali Torres, Iris Manosalva, Nathalie Sakakini, Cyril Esnault, Sandrine Sarrazin, Michael Sieweke, and Salvatore Spicuglia

Corresponding author(s): Salvatore Spicuglia (salvatore.spicuglia@inserm.fr)

Review Timeline:

Submission Date:	1st Apr 25
Editorial Decision:	2nd Jun 25
Revision Received:	27th Aug 25
Editorial Decision:	23rd Oct 25
Revision Received:	29th Oct 25
Accepted:	25th Nov 25

Editor: Cornelius Schneider

Transaction Report:

(Note: With the exception of the correction of typographical or spelling errors that could be a source of ambiguity, letters and reports are not edited. Depending on transfer agreements, referee reports obtained elsewhere may or may not be included in this compilation. Referee reports are anonymous unless the Referee chooses to sign their reports.)

1st Editorial Decision 2nd Jun 2025

Dear Dr. Spicuglia,

Thank you for submitting your manuscript for consideration by the EMBO Journal. It has now been seen by two referees whose comments are shown below.

Both referees think that the detailed identification and mapping of epromoters is interesting and relevant. However, both also think that the analysis is still premature and we agree with the referees that the inclusion of more recent dataset and the consideration of 3D genome organization would benefit the manuscript significantly. We also agree with referee #1 that it is important that readers can easily recapitulate the pipeline. I find the points raised by the referees helpful and productive. I would therefore like to invite you to submit a revised version of the manuscript, addressing all the comments by the reviewers. I should add that it is EMBO Journal policy to allow only a single round of revision, and acceptance of your manuscript will therefore depend on the completeness of your responses in this revised version. I am happy to discuss any additional questions via e-mail or video conferencing.

When preparing your letter of response to the referees' comments, please bear in mind that this will form part of the Review Process File, and will therefore be available online to the community. For more details on our Transparent Editorial Process, please visit our website: https://www.embopress.org/page/journal/14602075/authorguide#transparentprocess

We generally allow three months as standard revision time. As a matter of policy, competing manuscripts published during this period will not negatively impact on our assessment of the conceptual advance presented by your study. However, we request that you contact the editor as soon as possible upon publication of any related work, to discuss how to proceed. Should you foresee a problem in meeting this three-month deadline, please let us know in advance and we may be able to grant an extension.

Thank you for the opportunity to consider your work for publication. I look forward to your revision.

Yours sincerely,

Cornelius Schneider, PhD
Editor
The EMBO Journal
c.schneider@embojournal.org

Instructions for preparing your revised manuscript:

Please make sure you upload a letter of response to the referees' comments together with the revised manuscript.

Please also check that the title and abstract of the manuscript are brief, yet explicit, even to non-specialists.

When assembling figures, please refer to our figure preparation guideline in order to ensure proper formatting and readability in print as well as on screen:
https://bit.ly/EMBOPressFigurePreparationGuideline
See also guidelines for figure legends: https://www.embopress.org/page/journal/14602075/authorguide#figureformat

At EMBO Press we ask authors to provide source data for the main manuscript figures. You will receive a separate email with instructions for providing source data with your revised manuscript, including how to upload and organize the files.

IMPORTANT: When you send the revision we will require
- a point-by-point response to the referees' comments, with a detailed description of the changes made (as a word file).
- a word file of the manuscript text.
- individual production quality figure files (one file per figure)
- a complete author checklist, which you can download from our author guidelines (https://www.embopress.org/page/journal/14602075/authorguide).
- Expanded View files (replacing Supplementary Information)
Please see out instructions to authors
https://www.embopress.org/page/journal/14602075/authorguide#expandedview
- a Reagents and Tools Table as part of the Methods section, which can be downloaded from our author guidelines (https://www.embopress.org/page/journal/14602075/authorguide#structuredmethods)

Please remember: Digital image enhancement is acceptable practice, as long as it accurately represents the original data and conforms to community standards. If a figure has been subjected to significant electronic manipulation, this must be noted in the figure legend or in the 'Materials and Methods' section. The editors reserve the right to request original versions of figures and the original images that were used to assemble the figure.

Further information is available in our Guide For Authors: https://www.embopress.org/page/journal/14602075/authorguide

We realize that it is difficult to revise to a specific deadline. In the interest of protecting the conceptual advance provided by the work, we recommend a revision within 3 months (31st Aug 2025). Please discuss the revision progress ahead of this time with the editor if you require more time to complete the revisions. Use the link below to submit your revision:

https://emboj.msubmit.net/cgi-bin/main.plex

Referee #1:

Summary and opinion

Malfait J et al. developed a pipeline to identify Epromoters and their potential target genes across various stress conditions. The authors applied this pipeline to publicly available transcriptomic and ChIP-seq data from 51 studies and identified clusters of genes regulated by Epromoters across 18 distinct stress conditions. Based on the in-silico analysis, the authors propose that stress-induced genes are organized into clusters, and that in each cluster a promoter with enhancer activity (Epromoter) is bound by a key inducible transcription factor to regulate the expression of genes in the same cluster, thereby obtaining a fast and coordinated response to stress stimuli. On the bench, Malfait J et al. performed reporter assays and CRISPR-Cas9 genomic deletions to determine Epromoter activity and to demonstrate the regulatory influence of Epromoters on neighboring genes within their respective clusters. Additionally, the authors repositioned one the Epromoters within its locus to further confirm its intrinsic enhancer and promoter functions, underscoring the dual role of Epromoters in gene regulation. Based on all their results, the authors conclude that Epromoters have a key role in the induction of co-regulated genes during the response of mammalian cells to different stress stimuli.

Major comments:

1. The lab from Salvatore Spicuglia has performed pioneer work characterizing regulatory elements with promoter and enhancer activity (Epromoters) and published several landmark articles on this topic (Wan J et al., Nucleic Acids Res. 2024; Santiago-Algarra D et al., Nat Commun. 2021; Dao LTM et al., Nat Genet. 2017). In the manuscript EMBOJ-2025-120952, Malfait J et al. extended the knowledge about Epromoters by analyzing publicly available next-generation sequencing (NGS) data from 51 different studies. They identified clusters of genes induced by 18 distinct stress stimuli through Epromoters. However, some of the studies included in their analysis are relatively old, and relatively recent studies based on new stress models were not included. Thus, I suggest the authors to include also new stress models based on more recent publications.

2. Malfait J et al. developed a pipeline to identify Epromoters and their potential target genes by applying it to NGS data. I used the pipeline to analyze some of the NGS data sets reported in the manuscript and was able to partially reproduce the results presented by the authors. I believe that many other colleagues working in the field of transcriptional regulation would benefit by implementing the pipeline in their respective projects if the author could make it more accessible, for example by making a web application to easily use the pipeline and test different parameters. In addition, the article by Malfait J et al. and the pipeline will gain visibility that may be translated into more citations if this web application is linked to the manuscript. Thus, I suggest to the authors to generate such a web application to easily access and use the pipeline and to link the web page to the manuscript.

3. The results obtained from the CRISPR-Cas9 mediated genomic deletion of the NUCB1 Epromoter in K562 cells, and the reinsertion of the NUCB1 Epromoter 3´ of the DHDH gene are impressive. The authors should explain their rationale for selecting the locus for the reinsertion of the NUCB1 Epromoter 3´ of the DHDH gene. In addition, the authors should include in the figures 4E and 4F the expression in wild-type K562 cells.

4. Malfait J et al. propose in this manuscript a hypothesis for Epromoter hijacking during evolution, using the OASL/P2RX7 locus as example (Figure S4). This hypothesis is very appealing and interesting. I would like to suggest that the authors validate this hypothesis. For example, the authors could use the CRISPR-Cas9 technology in a human cell line for this purpose.

5. The authors should provide in a Source Data file (e.g. in an Excel file) the information that they used for the elaboration of the different plots presented in the main and supplementary figures, including single values, means, medians, error bars, p-values, test used of their calculations, etc.

Minor comments:

6. In the legend of the figure 2G is written:

"(G) Luciferase assays to quantify the enhancer activity of predicted Epromoter (on the right) and induced promoters clustered with Epromoters (on the left)"

Please correct the legend. In the corresponding figure panel, the predicted Epromters are on the left and the induced promoters are on the right.

Overall, the manuscript by Malfait J et al. constitutes a significant contribution to the fields of transcription regulation, chromatin structure and 3D genome organization. The design of the bioinformatic analysis is comprehensive, and the methods implemented are cutting-edge. EMBO J will be the right platform to make this contribution accessible to the scientific community. However, I recommend a minor revision of the manuscript before publication at EMBO J. I hope that my comments support the authors to improve the manuscript to reach the standard of manuscripts recently published at EMBO J.

Referee #2:

The manuscript by Malfait and colleagues addresses the significance of Epromoters (originally also a seminal discovery by the Spicuglia team) in the regulation of stress-responsive genes in human and mouse cells. To this end, the authors revisited a large number of publicly available datasets on gene expression and TF binding from a multitude of cell types and performed analyses to propose that such stress response genes are more likely that other gene sets to be clustered and under the control of TF hubs forming on Epromoters. Overall, this work is an extended validation of earlier work (properly cited by the authors) showing this postulation to be true for IFN-responsive genes. Of course, such extended validation for key stress responses (e.g., hypoxia, insulin, TNF, serum) is welcome and is well-executed here. At the same time though, it was not unknown that genes in these pathways tend to be organized in linear clusters (and even to be coordinated in 3D nuclear space when active). Thus, the major contribution of this manuscript lies in the potential characterization of Epromoters in this context, which still needs more strengthening regarding its mechanistic attributes.
Below, I elaborate on a number of points (in the order of appearance in the text) that I hope the authors will find useful when revising their manuscript:
- The control group of randomly selected genes could be substantially improved (and likely be more fair) if the authors selected genes belonging to another pathway or co-expressed housekeeping genes in the cell type studied. It is also unclear to me (perhaps I missed it in the Methods section) whether these randomly selected genes also had to be active and to which extent (preferably to the mean of the induced genes in question).
- Similarly, one might want to use somewhat "faster" responses as control than the broad meso-, ecto-, and endoderm differentiation processes. For example, retinoic acid or some differentiation path that relies on a single master TF, like the C/EBP-driven transdifferentiation system by Thomas Graf's lab might be better candidates.
- To this end, one should note that, for mammalian regulatory space, distribution summits of 55-65 kbp in stress-response versus ~120 kbp random genes are not of majestic difference as they would all fall within TAD/subTAD structures on average.
- I understand the criteria set for predicting Epromoter-regulated gene clusters, but what remains a bit vague still is (1) whether there are also enhancers interspersed in these clusters and how the authors treat these elements, and (2) how the criterion of "a cluster [was deemed] potentially regulated by an Epromoter if the number of induced genes was higher than the number of genes displaying TF binding at their promoters" is mechanistically thought of, since for the proinflammatory response, for example, regulation often finds NFkappaB bound to both the enhancers and the promoters of responsive genes (see Kolovos et al, Genome Res 2016) or even to a given "master" enhancer seemingly controlling many downstream genes in a cluster (e.g., the CXCL cluster in Weiterer et al, EMBO J 2020).
- Along the same lines, it would be nice if the authors tried to align their data on the CXCL locus to those by Weiterer et al (2020) where the same data is available, but the stimulus is IL1a and the regulation is mainly attributed to a "master" enhancer upstream the gene cluster.
- Unless I am mistaken, the density of TF motifs in Epromoters does not co-consider chromatin accessibility and this means that many of the motifs interrogated could very well be hidden, especially in the case of TFs like NF-kappaB that has hundreds of thousands of motifs genome-wide. I am pretty sure that ATAC-seq (or DNase I-seq) data will exist for 1-2 exemplary responses, and it would be very useful if the authors repeated their enrichment analyses by integrating this functionally relevant information.
- The OASL-P2RX7 example is very nice, but makes me wonder conceptually about the model: is P2RX7 indeed functionally co-opted in the IFNgamma response in human compared to mice? Is the activation of this gene indeed a functional part of the response? Or is it just that it get upregulated due to "activity spillover" in the OASL-dominated domain upon IFN stimulation? Or put more generally, maybe Epromoters rich in motifs for a given stress-responsive TF are meant to activate other similar promoters (i.e., that have similar, if a bit less, TF composition) and most activation of genes not binding that TF is a side-effect of their high activation? Could the authors put numbers on this prediction and see if they will confirm or rule it out?
- On two more minor notes: First, I would have expected more use of 3D genomics (Hi-C, Micro-C) data in those cases where they are available in visualizing also the 3D regulatory aspect of these clusters. Second, some clarification for Fig S5 would be

nice, since the browser screenshot shown SREBP binding, but TNF-responsive genes are almost ubiquitously regulated via NF-kappaB binding; am I missing something? What does p65 look like in this locus and does it align to the Epromoter model?

I typically disclose my identity to the authors: A. Papantonis

Point-by-point response to Reviewers' Comments:

We thank both referees for their positive and constructive comments, which we have tried to address in their entirety. Among the main changes, we have performed experimental validation of P2RX7 by the OASL Epromoter, added more recent datasets, performed additional controls for the distance distribution between induced genes, explored the distal and proximal binding of HSF1 in the heat-shock response and developed a web-based tool to identify potential Epromoter-regulated clusters. Together with a more detailed description of the results and discussion, we believe that our study has substantially improved and we hope that the referees will find our revised manuscript suitable for publication. The amendments introduced in response to their feedback are highlighted in red in the revised manuscript file.

Referee #1:

Summary and opinion

Malfait J et al. developed a pipeline to identify Epromoters and their potential target genes across various stress conditions. The authors applied this pipeline to publicly available transcriptomic and ChIP-seq data from 51 studies and identified clusters of genes regulated by Epromoters across 18 distinct stress conditions. Based on the in-silico analysis, the authors propose that stress-induced genes are organized into clusters, and that in each cluster a promoter with enhancer activity (Epromoter) is bound by a key inducible transcription factor to regulate the expression of genes in the same cluster, thereby obtaining a fast and coordinated response to stress stimuli. On the bench, Malfait J et al. performed reporter assays and CRISPR-Cas9 genomic deletions to determine Epromoter activity and to demonstrate the regulatory influence of Epromoters on neighboring genes within their respective clusters. Additionally, the authors repositioned one the Epromoters within its locus to further confirm its intrinsic enhancer and promoter functions, underscoring the dual role of Epromoters in gene regulation. Based on all their results, the authors conclude that Epromoters have a key role in the induction of co-regulated genes during the response of mammalian cells to different stress stimuli.

Major comments:

1. The lab from Salvatore Spicuglia has performed pioneer work characterizing regulatory elements with promoter and enhancer activity (Epromoters) and published several landmark articles on this topic (Wan J et al., Nucleic Acids Res. 2024; Santiago-Algarra D et al., Nat Commun. 2021; Dao LTM et al., Nat Genet. 2017). In the

manuscript EMBOJ-2025-120952, Malfait J et al. extended the knowledge about Epromoters by analyzing publicly available next-generation sequencing (NGS) data from 51 different studies. They identified clusters of genes induced by 18 distinct stress stimuli through Epromoters. However, some of the studies included in their analysis are relatively old, and relatively recent studies based on new stress models were not included. Thus, I suggest the authors to include also new stress models based on more recent publications.

Answer R1#1:

For the initial submission, we searched in the literature for datasets that were published before 2022. Following the reviewer's suggestion, we searched for new datasets either from published studies or present in the GEO repository. We found four consistent studies, including Purbey et al. 2024 (2 datasets), GSE158529 and GSE278100 (see Table EV1). We also included the study from Jurida et al. Cell Report 2015, as suggested by Reviewer #2. These five datasets were fully analyzed and the results provided in the revised Figures 1 and 3, Figure EV1, as well as Dataset EV1, and Tables EV1-EV2. Overall, we were able to identify Epromoter's clusters in all the new datasets. One example of an identified cluster is provided in Figure 3D (locus *Nfk2b*).

2. Malfait J et al. developed a pipeline to identify Epromoters and their potential target genes by applying it to NGS data. I used the pipeline to analyze some of the NGS data sets reported in the manuscript and was able to partially reproduce the results presented by the authors. I believe that many other colleagues working in the field of transcriptional regulation would benefit by implementing the pipeline in their respective projects if the author could make it more accessible, for example, by making a web application to easily use the pipeline and test different parameters. In addition, the article by Malfait J et al. and the pipeline will gain visibility that may be translated into more citations if this web application is linked to the manuscript. Thus, I suggest to the authors to generate such a web application to easily access and use the pipeline and to link the web page to the manuscript.

Answer R1#2:

We thank the reviewer for this interesting suggestion. We have implemented our pipeline to identify Epromoter-regulated clusters in a user-friendly web-based tool. The web tool is openly accessible at https://epromoters.tagc.univ-amu.fr. It only requires a list of induced genes and a bed file that contains the binding sites for a transcription factor. Together with the available scripts in the GITHUB repository, this web tool will ensure that anybody can easily recapitulate the pipeline.

3. The results obtained from the CRISPR-Cas9 mediated genomic deletion of the NUCB1 Epromoter in K562 cells, and the reinsertion of the NUCB1 Epromoter 3´ of the DHDH gene are impressive. The authors should explain their rationale for selecting the locus for the reinsertion of the NUCB1 Epromoter 3´ of the DHDH gene. In addition, the authors should include in the figures 4E and 4F the expression in wild-type K562 cells.

Answer R1#3:

We selected the site of the insertion downstream of DHDH to rule out any influence from transcripts coming from the NUCB1 Epromoter. Moreover, the site of the insertion was located in a genome region deprived of active histone marks (H3K4me3 and H3K27ac) in K562 but inside the same TAD as NUCB1 and DHDH to ensure the Epromoter remains within the same chromatin context. This is now better explained in the main text (lines 341-345). As requested by the reviewer, we have included the expression in wild-type cells in Figures 4E and 4F.

4. Malfait J et al. propose in this manuscript a hypothesis for Epromoter hijacking during evolution, using the OASL/P2RX7 locus as an example (Figure EV4). This hypothesis is very appealing and interesting. I would like to suggest that the authors validate this hypothesis. For example, the authors could use the CRISPR-Cas9 technology in a human cell line for this purpose.

Answer R1#4:

We thank the reviewer for this interesting suggestion. We first validated that both *OASL* and *P2RX7* could be induced by IFNγ in THP1 cells (a model of human monocytes). As shown in the new Figure EV4B, we achieved consistent induction of both genes at 8h and 24h of IFNγ treatment in ThP1 cells. To assess the role of *OASL* promoter in the induction of *P2RX7* we used a CRISPR inactivation system. We generated THP1 cells stably expressing the dCas9-KRAB-MeCP2 cassette, either as a pool or a clonal cell line (see Materials and Methods section). Lentiviral expression of 2 different sgRNA targeting the *OASL* promoter completely abolished *OASL* expression as compared to a control sgRNA (Figure EV4B). Strikingly, the two *OASL* sgRNAs also significantly reduced the induction of *P2RX7* after IFNγ treatment, suggesting that the *OASL* Epromoter contributes to the induction of *P2RX7* in human cells. These results thus support the co-optation of P2RX7 into the inflammatory response by hijacking the OASL Epromoter in humans. The text describing these new results is presented in lines 430-438.

5. The authors should provide in a Source Data file (e.g. in an Excel file) the information that they used for the elaboration of the different plots presented in the main and supplementary figures, including single values, means, medians, error bars, p-values, test used of their calculations, etc.

Answer R1#5:

We have followed the instructions provided by EMBO press to generate a comprehensive Source Data file.

Minor comments:

6. In the legend of the figure 2G is written:
"(G) Luciferase assays to quantify the enhancer activity of predicted Epromoter (on the right) and induced promoters clustered with Epromoters (on the left)"
Please correct the legend. In the corresponding figure panel, the predicted Epromoters are on the left and the induced promoters are on the right.

Answer R1#6:

This is now corrected

Overall, the manuscript by Malfait J et al. constitutes a significant contribution to the fields of transcription regulation, chromatin structure and 3D genome organization. The design of the bioinformatic analysis is comprehensive, and the methods implemented are cutting-edge. EMBO J will be the right platform to make this contribution accessible to the scientific community. However, I recommend a minor revision of the manuscript before publication at EMBO J. I hope that my comments support the authors to improve the manuscript to reach the standard of manuscripts recently published at EMBO J.

Referee #2:

The manuscript by Malfait and colleagues addresses the significance of Epromoters (originally also a seminal discovery by the Spicuglia team) in the regulation of stress-responsive genes in human and mouse cells. To this end, the authors revisited a large number of publicly available datasets on gene expression and TF binding from a

multitude of cell types and performed analyses to propose that such stress response genes are more likely that other gene sets to be clustered and under the control of TF hubs forming on Epromoters. Overall, this work is an extended validation of earlier work (properly cited by the authors) showing this postulation to be true for IFN-responsive genes. Of course, such extended validation for key stress responses (e.g., hypoxia, insulin, TNF, serum) is welcome and is well-executed here. At the same time though, it was not unknown that genes in these pathways tend to be organized in linear clusters (and even to be coordinated in 3D nuclear space when active). Thus, the major contribution of this manuscript lies in the potential characterization of Epromoters in this context, which still needs more strengthening regarding its mechanistic attributes.

Below, I elaborate on a number of points (in the order of appearance in the text) that I hope the authors will find useful when revising their manuscript:

- The control group of randomly selected genes could be substantially improved (and likely be more fair) if the authors selected genes belonging to another pathway or co-expressed housekeeping genes in the cell type studied. It is also unclear to me (perhaps I missed it in the Methods section) whether these randomly selected genes also had to be active and to which extent (preferably to the mean of the induced genes in question).

Answer R2#1:

As correctly mentioned by the reviewer, it has already been observed that stress response genes are preferentially organized in gene clusters and we acknowledged in the manuscript some of these findings (Refs 5 to 10). In our present study, we took advantage of our comprehensive dataset of stress response models to address this question in a more systematic way, which, to our knowledge, has not been done before. As a comparison, we used randomly selected genes (without taking into account their expression levels) as we wanted to determine whether stress response genes are located in closer genomic distances than randomly expected. This said, we also agree with the reviewer that comparison with other control groups will also be important. As suggested by the reviewer, we compared the distance distribution between the set of induced genes and a set of similarly expressed genes but not induced by the stress conditions. We focused on the two stress conditions presented in the main Figure 1B (heat shock "Vihervaara" dataset and TNFa "Park" dataset). As shown in the new Figure EV1C, we observed a significantly closer distance distribution for the set of induced genes in both cases. These new results are described in the results sections (lines 175-176).

- Similarly, one might want to use somewhat "faster" responses as control than the broad meso-, ecto-, and endoderm differentiation processes. For example, retinoic acid or some differentiation path that relies on a single master TF, like the C/EBP-driven transdifferentiation system by Thomas Graf's lab might be better candidates.

Answer R2#2:

We also agreed that it might be interesting to also look at more specific differentiation systems. However, instead of analyzing the B-cell to macrophage transdifferentiating system, we explored a dataset from Susan Mandrup's lab providing transcriptome for *in vitro* differentiation of immortalized human mesenchymal stromal cells into either adipocytes or osteoblasts (Madsen et al. Nat. Genetics 2020). As shown in the new figures 1C-E and S1B, genes induced by the differentiation into either osteoblast or adipocytes displayed a similar distance distribution to random genes. These new results are described in the results sections (lines 147-161).

- To this end, one should note that, for mammalian regulatory space, distribution summits of 55-65 kbp in stress-response versus ~120 kbp random genes are not of majestic difference as they would all fall within TAD/subTAD structures on average.

Answer R2#3:

The distribution summit was provided as quantifiable values, but the statistical comparisons (K-S tests and deviation scores) were calculated with the full list of genes, providing a more precise assessment of the differences in the distribution distances.

- I understand the criteria set for predicting Epromoter-regulated gene clusters, but what remains a bit vague still is (1) whether there are also enhancers interspersed in these clusters and how the authors treat these elements, and (2) how the criterion of "a cluster [was deemed] potentially regulated by an Epromoter if the number of induced genes was higher than the number of genes displaying TF binding at their promoters" is mechanistically thought of, since for the proinflammatory response, for example, regulation often finds NFkappaB bound to both the enhancers and the promoters of responsive genes (see Kolovos et al, Genome Res 2016) or even to a given "master" enhancer seemingly controlling many downstream genes in a cluster (e.g., the CXCL cluster in Weiterer et al, EMBO J 2020).

Answer R2#4:

Regarding the first part of the question: In our previous study related to the IFNa response, we analyzed the distance of ISGF3-regulatory elements (=regions bound by STAT1/2 and IRF9 transcription factors) with respect to the promoters of induced genes (Figure 3 of Santiago et al. Nat Com 2021). We obtained two interesting findings. On the one hand, for IFNa-induced genes, there was a significant enrichment of finding the closest ISGF3-regulatory element in the promoter of a neighbor gene. On the other hand, we found that induced genes outside clusters were preferentially associated with an ISGF3 complex located in an intergenic region (i.e., distal enhancers), while induced genes within clusters were preferentially associated with an ISGF3 complex located in a promoter region. Of course, this is not a black and white situation, but rather suggests that genes in clusters are likely to be regulated by Epromoter-like regulatory elements. These results are discussed in our current manuscript in the context of our new findings (lines 553-564). To further explore this question, we quantify the number of proximal and distal HSF1 binding within the set of clusters induced by Heat Shock ("Vihervaara" dataset; new Figure EV2E). Next, we defined for each cluster whether there was a proximal or distal HSF1 binding, or both (new Figure EV2F). Interestingly, we found that 71% of Epromoter's clusters were associated with only proximal HSF1 binding, while the remaining 29% were associated with both proximal and distal HSF1 binding. Therefore, while some clusters might be regulated by both Epromoter and distal enhancer elements, the majority of Epromoter-regulated clusters induced by HS appeared to be regulated by proximal regulatory elements. These results are described in the results and discussion sections (lines 261-267; 553-564)

Regarding the second part of the question: we fully agree with the reviewer that different types of regulation might exist and that this depends on the involved TFs and the stimulation/stress conditions. We have now added a paragraph in the discussion section to state this limitation of our study and highlight the NfkappaB example (lines 558-564).

- Along the same lines, it would be nice if the authors tried to align their data on the CXCL locus to those by Weiterer et al (2020) where the same data is available, but the stimulus is IL1a and the regulation is mainly attributed to a "master" enhancer upstream the gene cluster.

Answer R2#5:

Our results show that the *Cxcl1* promoter partially contributes to *Cxcl2* induction in mouse 3T3 cells stimulated by TNFα, but it is likely that other cis-regulatory elements contribute to its regulation. However, as pointed out by the reviewer, we think that this regulation might be specific to the mouse, as we did not identify the *CXCL1-CXCL2* locus as a potential Epromoter-regulated cluster in human models of TNFα stimulation

(Dataset EV1). This might be due, in part, to differences in locus configuration between the two species (i.e., while *Cxcl2* is a direct neighbour of *Cxcl1* in the mouse, these genes are separated by several other genes in humans). Moreover, p65 binds the *CXCL2* promoter in human epithelial KB cells stimulated with IL-1α (Jurida et al. Cell Reports 2016), and is partially regulated by distal enhancers (Weiterer et al. EMBO J 2020). These differences between human and mouse are now highlighted in the manuscript (lines 383-388).

- Unless I am mistaken, the density of TF motifs in Epromoters does not co-consider chromatin accessibility and this means that many of the motifs interrogated could very well be hidden, especially in the case of TFs like NF-kappaB that has hundreds of thousands of motifs genome-wide. I am pretty sure that ATAC-seq (or DNase I-seq) data will exist for 1-2 exemplary responses, and it would be very useful if the authors repeated their enrichment analyses by integrating this functionally relevant information.

Answer R2#6:

As mentioned by the reviewer, the analyses to quantify the density of TF motifs for the heat-shock response did not consider chromatin accessibility. Indeed, our goal here was to assess whether the number of binding sites at promoters might be an intrinsic feature to explain why some promoters display enhancer activity. This is now clarified in the manuscript (line 247-248).

- The OASL-P2RX7 example is very nice, but makes me wonder conceptually about the model: is P2RX7 indeed functionally co-opted in the IFNgamma response in human compared to mice? Is the activation of this gene indeed a functional part of the response? Or is it just that it get upregulated due to "activity spillover" in the OASL-dominated domain upon IFN stimulation? Or put more generally, maybe Epromoters rich in motifs for a given stress-responsive TF are meant to activate other similar promoters (i.e., that have similar, if a bit less, TF composition) and most activation of genes not binding that TF is a side-effect of their high activation? Could the authors put numbers on this prediction and see if they will confirm or rule it out?

Answer R2#7:

We agree with the reviewer that whether associated genes are functionally relevant for the stress response is a very interesting question. While this is out of the scope of the present study, we have extensively discussed the implications of our findings for the understanding of the evolution of regulatory elements and stress-response clusters (see lines 613-639). Concerning the motif analyses, we have found that HS-Epromoters

harbor a significantly higher number of HSF binding sites while associated gene promoters are depleted of HSF motifs (Figure 2I), supporting a model whereby the co-induced promoters are dependent on the binding of HSF1/HSF2 at Epromoters within the same cluster. Moreover, we also suited to experimentally assess the role of *OASL* promoter in regulating *P2RX7* response to IFNγ stimulation in humans (also requested by Reviewer #1, see answer R1#4). By performing CRISPR-based inactivation of the *OASL* promoter in human THP1 cells, we observed a significant reduction in *P2RX7* induction after IFNγ stimulation (Figure EV4B), suggesting that the *OASL* Epromoter contribute to the IFNγ response of *P2RX7* in human cells.

- On two more minor notes: First, I would have expected more use of 3D genomics (Hi-C, Micro-C) data in those cases where they are available in visualizing also the 3D regulatory aspect of these clusters. Second, some clarification for Fig S5 would be nice, since the browser screenshot shown SREBP binding, but TNF-responsive genes are almost ubiquitously regulated via NF-kappaB binding; am I missing something? What does p65 look like in this locus and does it align to the Epromoter model?

Answer R2#8:

Regarding 3D genomics. We have used 3D genomics in the context of the HS response to compare the identification of clusters based either on TAD borders or distance between the genes (Figure 2). We also used available 3D genomic data to assess *PDL1-JAK2* interactions (Figure 5). Although we fully agree with the reviewer that the study of the 3D interactions mediated by Epromoter clusters is an important issue, we believe this is out of the scope of our current study and will surely be the focus of future work in our lab. We have, however, mentioned this limitation and extensively discussed our study in the context of 3D genome organization and transcriptional condensates in the Discussion section (lines 496-507; 587-612).

Regarding the question about Figure EV5. The goal of this additional figure was to illustrate the observation that for some clusters only a limited number of promoters do not bind the key TF. We agree with the reviewer that binding of SREBP is not the best example in this context and we have replaced this locus by another cluster from LPS-stimulated BMDM (cluster *Phf11*; see new Figure EV5). GBP genes are indeed bound by p65 after different inflammatory stimulus (see Dataset EV1) and predicted to be regulated by Epromoters (see example below from the “Hogan” dataset).

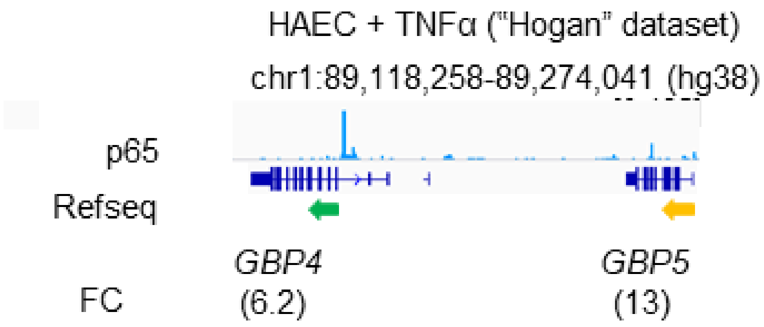

I typically disclose my identity to the authors: A. Papantonis

Dear Dr. Spicuglia,

Thank you for submitting a revised version of your manuscript. Your study has now been seen by the original referee #2, who finds that the previous concerns have been addressed and now recommends publication of the manuscript. We could not reach the original referee #1. Given that the report of this referee during the first round of review was already positive we decided to proceed with the manuscript with the additional feedback provided by referee #2.
There remain only a few mainly editorial points that have to be addressed before I can extend formal acceptance of the manuscript:

- On the abstract page of the manuscript, please include 4-5 general keyword terms to enhance searchability.

- Please rename the "Data and materials availability" section to "Data Availability"

- Please rename the Conflict of Interest section into "Disclosure and Competing Interests Statement", in accordance with our updated Guide to Authors (https://www.embopress.org/competing-interests)

- As we are switching from a free-text author contribution statement towards a more formal statement based on Contributor Role Taxonomy (CRediT) terms, please remove the present Author Contribution section and instead specify each author's contribution(s) directly in the Author Information page of our submission system during upload of the final manuscript. See https://casrai.org/credit/ for more information.

- Please double-check to make sure to all relevant funding information in the manuscript is also entered into our submission system: the following appear to be missing as separate funders - Institut National de la Santé et de la Recherche Médicale (INSERM) and Aix-Marseille University; funding info has to be congruent so the authors should double check their entries and add the two funders via More Funders option (Comments box should not be used)

- Please upload the EV figures as separate production quality Figure files as they are currently provided in one PDF; Please also reformat Figure EV1 to be displayed on a single page.

- Please adjust the in-text callouts for individual figures and figure panels: e.g. Table EV4 appears to be missing

- Please provide suggestions for a short 'blurb' text prefacing and summing up the conceptual aspect of the study in two sentences (max. 250 characters), followed by 3-5 one-sentence 'bullet points' with brief factual statements of key results of the paper; they will form the basis of an editor-written 'Synopsis' accompanying the online version of the article. Please also provide an altered synopsis image, making sure that the aspect ratio conforms to our website's format - it should be exactly 550 pixels wide and between 300-600 pixels high.

- Please remove the REAGENT AND TOOLS TABLE form the manuscript file and upload as a separate file.

Our data editors also added the following requests regarding the figure legends:

- Please note that the exact p values are not provided in the legends of figures 2G, 4B, D, E, F; 5D, I; EV2 C, EV3, EV4B

- Please indicate the statistical test used for data analysis in the legend of figure EV2 G

- Please note that the box plots need to be defined in terms of minima, maxima, centre, bounds of box and whiskers, and percentile in the legend of figure 6E

- Please note that information related to n is missing in the legends of figures 1C, 6E

Please let me know if you have any questions regarding any of these points. You can use the link below to upload the revised files.
Thank you again for giving us the chance to consider your manuscript for The EMBO Journal. I look forward to receiving the final version.
With best regards,
Cornelius Schneider

Cornelius Schneider, PhD

Editor | The EMBO Journal
c.schneider@embojournal.org

Please refer to our figure preparation guideline in order to ensure proper formatting and readability in print as well as on screen:

https://bit.ly/EMBOPressFigurePreparationGuideline

See also figure legend guidelines:

https://www.embopress.org/page/journal/14602075/authorguide#figureformat

Use the link below to submit your revision:

https://emboj.msubmit.net/cgi-bin/main.plex

Referee #2:

I find that this revised version of the manuscript by Malfait and colleagues addresses all the points raised during the initial revision round, and I thank the authors for their detailed answers and changes to the manuscript. I am happy to endorse publication of this work in its current form.

Point by point response to the Editor

- On the abstract page of the manuscript, please include 4-5 general keyword terms to enhance searchability.
Done

- Please rename the "Data and materials availability" section to "Data Availability"
Done

- Please rename the Conflict of Interest section into "Disclosure and Competing Interests Statement", in accordance with our updated Guide to Authors (https://www.embopress.org/competing-interests)
Done

- As we are switching from a free-text author contribution statement towards a more formal statement based on Contributor Role Taxonomy (CRediT) terms, please remove the present Author Contribution section and instead specify each author's contribution(s) directly in the Author Information page of our submission system during upload of the final manuscript. See https://casrai.org/credit/ for more information.

Done

- Please double-check to make sure to all relevant funding information in the manuscript is also entered into our submission system: the following appear to be missing as separate funders - Institut National de la Santé et de la Recherche Médicale (INSERM) and Aix-Marseille University; funding info has to be congruent so the authors should double check their entries and add the two funders via More Funders option (Comments box should not be used)

Done

- Please upload the EV figures as separate production quality Figure files as they are currently provided in one PDF; Please also reformat Figure EV1 to be displayed on a single page.

We now provide the individual EV figures. Figure EV1 has been split into two EV figures. All EV numbers have been updated.

- Please adjust the in-text callouts for individual figures and figure panels: e.g. Table EV4 appears to be missing

We have now checked all callouts of the tables and figures. There was no Table EV4 and this mistake has been corrected.

- Please provide suggestions for a short 'blurb' text prefacing and summing up the conceptual aspect of the study in two sentences (max. 250 characters), followed by 3-5 one-sentence 'bullet points' with brief factual statements of key results of the paper; they will form the basis of an editor-written 'Synopsis' accompanying the online version of the article. Please also provide an

altered synopsis image, making sure that the aspect ratio conforms to our website's format - it should be exactly 550 pixels wide and between 300-600 pixels high.

Here are the suggestions:
'blurb' text:
Stress responses rewire transcription through specialized regulatory elements. This study reveals that Epromoters - dual elements with promoter-enhancer functions - orchestrate coordinated transcriptional responses of clustered genes to diverse stress signals.

'bullet points':
- Co-induced stress-response genes are often located in close genomic proximity.
- Only one promoter per cluster typically recruits key stress-activated transcription factors.
- These “hub” promoters show enhancer-like activity, consistent with Epromoter function.
- Epromoter-regulated clusters occur across different stress and inflammatory models.
- Epromoters enable coordinated regulation of gene clusters independently of genomic context.

Synopsis figure:

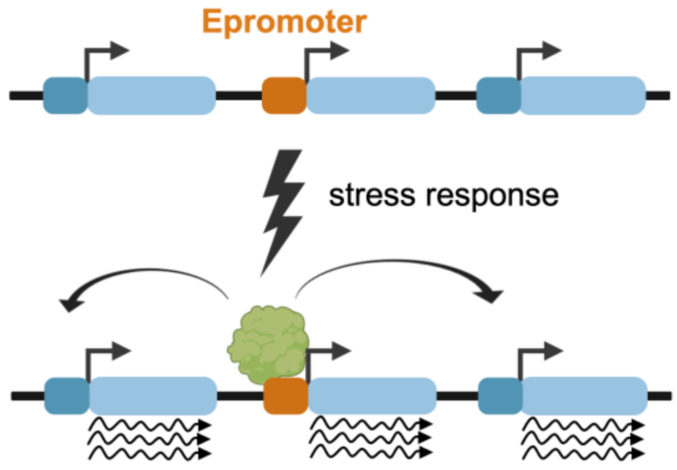

- Please remove the REAGENT AND TOOLS TABLE form the manuscript file and upload as a separate file.

Done

Our data editors also added the following requests regarding the figure legends:

- Please note that the exact p values are not provided in the legends of figures 2G, 4B, D, E, F; 5D, I; EV2 C, EV3, EV4B
Done

- Please indicate the statistical test used for data analysis in the legend of figure EV2 G.
Done (now EV3G)

- Please note that the box plots need to be defined in terms of minima, maxima, centre, bounds of box and whiskers, and percentile in the legend of figure 6E
Done

- Please note that information related to n is missing in the legends of figures 1C, 6E
Done

2nd Revision - Editorial Decision 25th Nov 2025

Dear Dr. Spicuglia,

I am pleased to inform you that your manuscript has been accepted for publication in the EMBO Journal.

Your manuscript will be processed for publication by EMBO Press. It will be copy edited and you will receive page proofs prior to publication. Please note that you will be contacted by Springer Nature Author Services to complete licensing and payment information.

You may qualify for financial assistance for your publication charges - either via a Springer Nature fully open access agreement or an EMBO initiative. Check your eligibility: https://www.embopress.org/page/journal/14602075/authorguide#chargesguide

Should you be planning a Press Release on your article, please get in contact with embo_production@springernature.com as early as possible in order to coordinate publication and release dates.

If you have any questions, please do not hesitate to contact the Editorial Office. Thank you for your contribution to The EMBO Journal.

Yours sincerely,

Cornelius Schneider, PhD
Editor
The EMBO Journal
c.schneider@embojournal.org

*****
Please note that it is The EMBO Journal policy for the transcript of the editorial process (containing referee reports and your response letters) to be published as an online supplement to each paper. If you should prefer removal of any referee-only figures included in the point-by-point response(s), e.g. because they may still be used for future publication or because they have been reproduced from published work by others, please do let us know immediately via response email.
More information is available here: https://www.embopress.org/transparent-process#Review_Process
*****

** Click here to be directed to your login page: https://emboj.msubmit.net

EMBO Press Author Checklist

Corresponding Author Name: Salvatore Spicuglia	
Journal Submitted to: The EMBO Journal	
Manuscript Number: EMBOJ-2025-120952	

USEFUL LINKS FOR COMPLETING THIS FORM
The EMBO Journal - Author Guidelines
EMBO Reports - Author Guidelines
Molecular Systems Biology - Author Guidelines
EMBO Molecular Medicine - Author Guidelines

Reporting Checklist for Life Science Articles (updated January
This checklist is adapted from Materials Design Analysis Reporting (MDAR) Checklist for Authors. MDAR establishes a minimum set of requirements in transparent reporting in the life sciences (see Statement of Task: 10.31222/osf.io/9sm4x). Please follow the journal's guidelines in preparing your
Please note that a copy of this checklist will be published alongside your article.

Abridged guidelines for figures
1. Data
The data shown in figures should satisfy the following conditions:
- the data were obtained and processed according to the field's best practice and are presented to reflect the results of the experiments in an accurate and unbiased manner.
- ideally, figure panels should include only measurements that are directly comparable to each other and obtained with the same assay.
- plots include clearly labeled error bars for independent experiments and sample sizes. Unless justified, error bars should not be shown for technical
- if n<5, the individual data points from each experiment should be plotted. Any statistical test employed should be justified.
- Source Data should be included to report the data underlying figures according to the guidelines set out in the authorship guidelines on Data

2. Captions
Each figure caption should contain the following information, for each panel where they are relevant:
- a specification of the experimental system investigated (eg cell line, species name).
- the assay(s) and method(s) used to carry out the reported observations and measurements.
- an explicit mention of the biological and chemical entity(ies) that are being measured.
- an explicit mention of the biological and chemical entity(ies) that are altered/varied/perturbed in a controlled manner.
- the exact sample size (n) for each experimental group/condition, given as a number, not a range;
- a description of the sample collection allowing the reader to understand whether the samples represent technical or biological replicates (including how many animals, litters, cultures, etc.).
- a statement of how many times the experiment shown was independently replicated in the laboratory.
- definitions of statistical methods and measures:
  - common tests, such as t-test (please specify whether paired vs. unpaired), simple χ2 tests, Wilcoxon and Mann-Whitney tests, can be unambiguously identified by name only, but more complex techniques should be described in the methods section;
  - are tests one-sided or two-sided?
  - are there adjustments for multiple comparisons?
  - exact statistical test results, e.g., P values = x but not P values < x;
  - definition of 'center values' as median or average;
  - definition of error bars as s.d. or s.e.m.

Please complete ALL of the questions below.
Select "Not Applicable" only when the requested information is not relevant for your study.

Materials

**Newly Created Materials**	**Information included in the manuscript?**	**In which section is the information available?** (Reagents and Tools Table, Materials and Methods, Figures, Data Availability Section)
New materials and reagents need to be available; do any restrictions apply?	Yes	Reagents and Tools Table, Materials and Methods

**Antibodies**	**Information included in the manuscript?**	**In which section is the information available?** (Reagents and Tools Table, Materials and Methods, Figures, Data Availability Section)
For **antibodies** provide the following information:<br>- Commercial antibodies: RRID (if possible) or supplier name, catalogue number and or/clone number<br>- Non-commercial: RRID or citation	Not Applicable	

**DNA and RNA sequences**	**Information included in the manuscript?**	**In which section is the information available?** (Reagents and Tools Table, Materials and Methods, Figures, Data Availability Section)
**Short novel DNA or RNA including primers, probes**: provide the sequences.	Yes	Reagents and Tools Table, Table EV3

**Cell materials**	**Information included in the manuscript?**	**In which section is the information available?** (Reagents and Tools Table, Materials and Methods, Figures, Data Availability Section)
**Cell lines:** Provide species information, strain. Provide accession number in repository **OR** supplier name, catalog number, clone number, and/**OR** RRID.	Yes	Materials and Methods
**Primary cultures:** Provide species, strain, sex of origin, genetic modification status.	Not Applicable	
Report if the cell lines were recently **authenticated** (e.g., by STR profiling) and tested for mycoplasma contamination.	Yes	Materials and Methods

**Experimental animals**	**Information included in the manuscript?**	**In which section is the information available?** (Reagents and Tools Table, Materials and Methods, Figures, Data Availability Section)
**Laboratory animals or Model organisms:** Provide species, strain, sex, age, genetic modification status. Provide accession number in repository **OR** supplier name, catalog number, clone number, **OR** RRID.	Not Applicable	
**Animal observed in or captured from the field:** Provide species, sex, and age where possible.	Not Applicable	
Please detail **housing and husbandry conditions**.	Not Applicable	

**Plants and microbes**	**Information included in the manuscript?**	**In which section is the information available?** (Reagents and Tools Table, Materials and Methods, Figures, Data Availability Section)
**Plants:** provide species and strain, ecotype and cultivar where relevant, unique accession number if available, and source (including location for collected wild specimens).	Not Applicable	
**Microbes:** provide species and strain, unique accession number if available, and source.	Yes	Materials and Methods

**Human research participants**	**Information included in the manuscript?**	**In which section is the information available?** (Reagents and Tools Table, Materials and Methods, Figures, Data Availability Section)
If collected and within the bounds of privacy constraints report on age, sex and gender or ethnicity for all study participants.	Not Applicable	

**Core facilities**	**Information included in the manuscript?**	**In which section is the information available?** (Reagents and Tools Table, Materials and Methods, Figures, Data Availability Section)
If your work benefited from core facilities, was their service mentioned in the acknowledgments section?	Yes	Acknowledgements

Design

Study protocol	Information included in the manuscript?	In which section is the information available? (Reagents and Tools Table, Materials and Methods, Figures, Data Availability Section)
If study protocol has been **pre-registered, provide DOI in the manuscript**. For clinical trials, provide the trial registration number **OR** cite DOI.	Not Applicable	
Report the **clinical trial registration number** (at ClinicalTrials.gov or equivalent), where applicable.	Not Applicable	

Laboratory protocol	Information included in the manuscript?	In which section is the information available? (Reagents and Tools Table, Materials and Methods, Figures, Data Availability Section)
Provide DOI OR other citation details if **external detailed step-by-step protocols** are available.	Not Applicable	

Experimental study design and statistics	Information included in the manuscript?	In which section is the information available? (Reagents and Tools Table, Materials and Methods, Figures, Data Availability Section)
Include a statement about **sample size** estimate even if no statistical methods were used.	Yes	Materials and Methods
Were any steps taken to minimize the effects of subjective bias when allocating animals/samples to treatment (e.g. **randomization procedure**)? If yes, have they been described?	Not Applicable	
Include a statement about **blinding** even if no blinding was done.	Yes	Materials and Methods
Describe **inclusion/exclusion criteria** if samples or animals were excluded from the analysis. Were the criteria pre-established?<br><br>If sample or data points were omitted from analysis, report if this was due to attrition or intentional exclusion and provide justification.	Not Applicable	
For every figure, are **statistical tests** justified as appropriate? Do the data meet the assumptions of the tests (e.g., normal distribution)? Describe any methods used to assess it. Is there an estimate of variation within each group of data? Is the variance similar between the groups that are being statistically compared?	Yes	Materials and Methods, Figure legends

Sample definition and in-laboratory replication	Information included in the manuscript?	In which section is the information available? (Reagents and Tools Table, Materials and Methods, Figures, Data Availability Section)
In the figure legends: state number of times the experiment was **replicated** in laboratory.	Yes	Figures, Figure legends, Materials and Methods
In the figure legends: define whether data describe **technical or biological replicates**.	Yes	Figure legends

Ethics

Ethics	Information included in the manuscript?	In which section is the information available? (Reagents and Tools Table, Materials and Methods, Figures, Data Availability Section)
Studies involving **human participants**: State details of **authority granting ethics approval** (IRB or equivalent committee(s), provide reference number for approval.	Not Applicable	
Studies involving **human participants**: Include a statement confirming that **informed consent** was obtained from all subjects and that the experiments conformed to the principles set out in the WMA Declaration of Helsinki and the Department of Health and Human Services Belmont Report.	Not Applicable	
Studies involving **human participants:** For publication of **patient photos**, include a statement confirming that consent to publish was obtained.	Not Applicable	
Studies involving experimental **animals**: State details of **authority granting ethics approval** (IRB or equivalent committee(s), provide reference number for approval. Include a statement of compliance with ethical regulations.	Not Applicable	
Studies involving **specimen and field samples:** State if relevant **permits** obtained, provide details of authority approving study; if none were required, explain why.	Not Applicable	

Dual Use Research of Concern (DURC)	Information included in the manuscript?	In which section is the information available? (Reagents and Tools Table, Materials and Methods, Figures, Data Availability Section)
Could your study fall under dual use research restrictions? Please check biosecurity documents and list of **select agents and toxins** (CDC): https://www.selectagents.gov/sat/list.htm	Not Applicable	
If you used a select agent, is the security level of the lab appropriate and reported in the manuscript?	Not Applicable	
If a study is subject to dual use research of concern regulations, is the name of the **authority granting approval and reference number** for the regulatory approval provided in the manuscript?	Not Applicable	

Reporting

The MDAR framework recommends adoption of discipline-specific guidelines, established and endorsed through community initiatives. Journals have their own policy about requiring specific guidelines and recommendations to complement MDAR.

Adherence to community standards	Information included in the manuscript?	In which section is the information available? (Reagents and Tools Table, Materials and Methods, Figures, Data Availability Section)
State if relevant guidelines or checklists (e.g., **ICMJE, MIBBI, ARRIVE, PRISMA**) have been followed or provided.	Not Applicable	
For **tumor marker prognostic studies,** we recommend that you follow the **REMARK** reporting guidelines (see link list at top right). See author guidelines, under 'Reporting Guidelines'. Please confirm you have followed these guidelines.	Not Applicable	
For **phase II and III randomized controlled trials**, please refer to the **CONSORT** flow diagram (see link list at top right) and submit the CONSORT checklist (see link list at top right) with your submission. See author guidelines, under 'Reporting Guidelines'. Please confirm you have submitted this list.	Not Applicable	

Data Availability

Data availability	Information included in the manuscript?	In which section is the information available? (Reagents and Tools Table, Materials and Methods, Figures, Data Availability Section)
Have **primary datasets** been deposited according to the journal's guidelines (see 'Data Deposition' section) and the respective accession numbers provided in the Data Availability Section?	Not Applicable	
Were **human clinical and genomic datasets** deposited in a public access-controlled repository in accordance to ethical obligations to the patients and to the applicable consent agreement?	Not Applicable	
Are **computational models** that are central and integral to a study available without restrictions in a machine-readable form? Were the relevant accession numbers or links provided?	Yes	Data and materials availability
If publicly available data were reused, provide the respective **data citations in the reference list**.	Yes	References list
